# Identification of Orbital Pumping from Spin Pumping and Rectification Effects

**DOI:** 10.1021/acs.nanolett.5c02641

**Published:** 2025-08-26

**Authors:** Nils Keller, Arnab Bose, Nozomi Soya, Elias Hauth, Fabian Kammerbauer, Rahul Gupta, Hiroki Hayashi, Hisanobu Kashiki, Gerhard Jakob, Sachin Krishnia, Kazuya Ando, Mathias Kläui

**Affiliations:** † Institute of Physics, 9182 Johannes Gutenberg University Mainz, Staudingerweg 7, 55128 Mainz, Germany; ‡ Department of Applied Physics and Physico-Informatics, 12869 Keio University, Yokohama 223-8522, Japan; ¶ Department of Electrical Engineering, 30077 Indian Institute of Technology Kanpur, Kanpur 208016, India; ◧ Keio Institute of Pure and Applied Science (KiPAS), Keio University, Yokohama 223-8522, Japan; ∥ Center for Spintronics Research Network (CSRN), Keio University, Yokohama 223-8522, Japan; ⊥ Graduate School of Excellence Materials Science in Mainz, 55099 Mainz, Germany; # Department of Physics, Center for Quantum Spintronics, Norwegian University of Science and Technology, 7491 Trondheim, Norway

**Keywords:** Orbital Pumping, Orbitronics, Orbital Torque, Magnetization Dynamics

## Abstract

The recently predicted mechanism of orbital pumping can enable the generation of pure orbital current from a precessing ferromagnet (FM) without the need for electrical current injection. This orbital current can be efficiently injected into an adjacent nonmagnetic material (NM) without being hampered by electrical conductivity mismatch. However, experimentally identifying this novel effect presents significant challenges due to the substantial background contributions from spin pumping and spin rectification effects (SREs). In this work, we disentangle the effects of orbital pumping from spin pumping in bilayer structures composed of Nb/Ni and Nb/Fe_60_Co_20_B_20_ by observing a sign reversal of the measured voltage. This reversal arises from the competing signs of the spin and orbital Hall effects in the Nb. We establish methods to differentiate the pumping signal from SREs by analyzing the distinct angular dependence of the measured voltage and its spatial dependence relative to the radio frequency excitation source.

## Graphical abstract

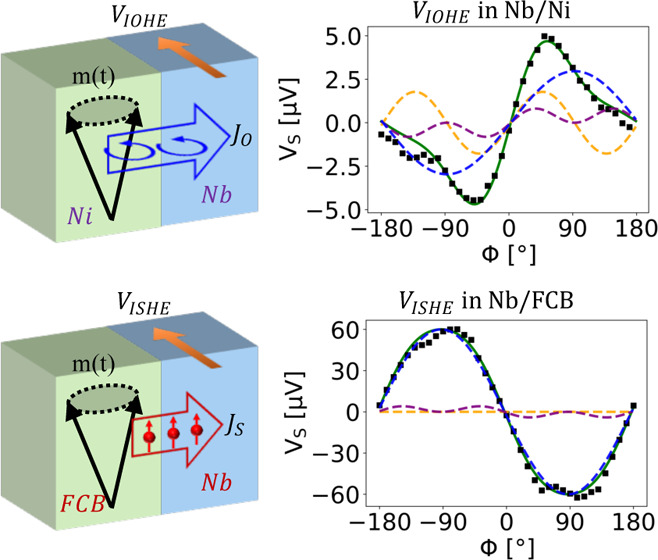

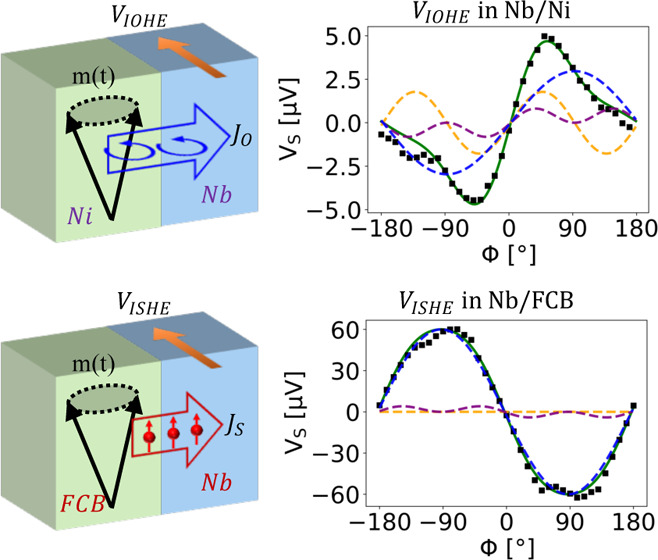

Spin and orbital angular momenta are two fundamental properties of electrons, interconnected through spin–orbit coupling (SOC). In spintronics, the SOC is essential in the emergence of various intriguing physical phenomena^1^ such as stabilization of chiral magnetic skyrmions^2^ and spin current generation mechanisms.^3^ The study of spin currents (*J*_S_), including those generated via the spin Hall effect (SHE)^4–7^ and the Rashba-Edelstein effect (REE),^8,9^ has been a central focus in spintronics, especially due to its potential applications in nonvolatile magnetic random-access memory (MRAM).^10,11^

The generation of orbital currents (*J*_O_) has recently attracted considerable interest, as they offer promising avenues for advancing energy-efficient MRAM technology.^12–14^ Theoretical studies indicate that orbital current is a fundamental entity that can, for example, generate spin currents through the SOC of materials.^13,15,16^ A major advantage of orbital currents is their potential to be orders of magnitude larger than spin currents across a wide range of materials,^17^ as they are not inherently limited by the relativistic SOC. Consequently, orbital currents may enable additional functionalities that overcome the inherent limitations of spin currents, particularly in terms of scalability and efficiency, making them highly promising for next-generation memory and logic applications.^12,13^

Thus far, the emerging field of orbitronics has mainly focused on the generation of *J*_O_ through the orbital Hall effect (OHE)^18–26^ and the orbital Rashba-Edelstein effect (OREE).^27–31^ Recently, theorists have predicted the effect of ”orbital pumping”, where a precessing magnet can emit a significant orbital current without requiring an associated electric current (Figure 1a).^32–34^ This effect is analogous to the previously demonstrated spin pumping effect, where a precessing magnet emits pure spin current (Figure 1b).^35–40^

**Figure 1:**
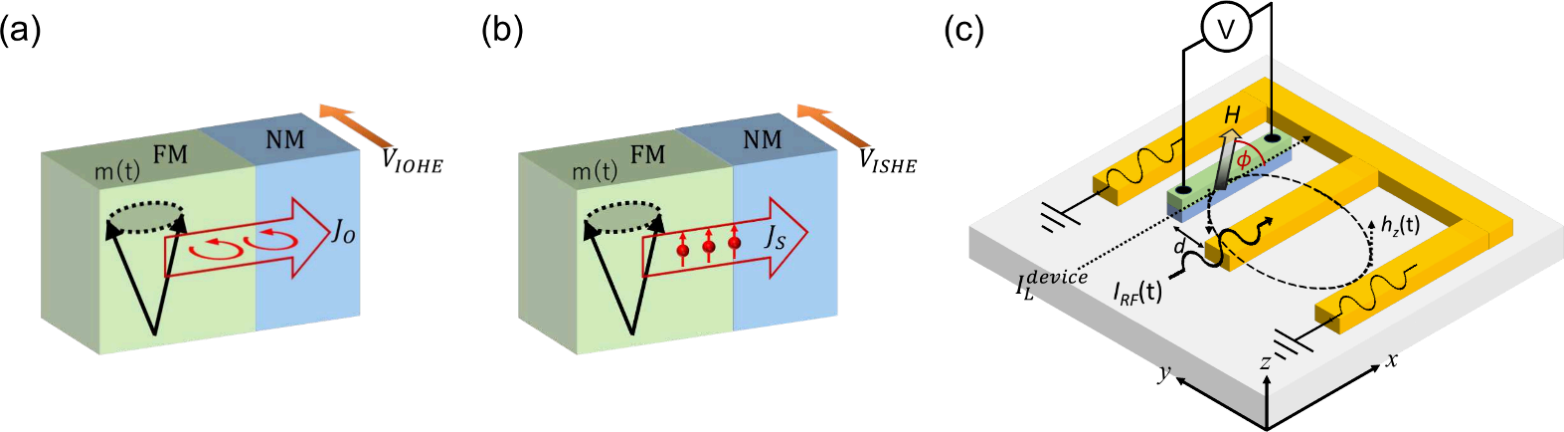
Schematic representation of (a) orbital pumping and (b) spin pumping from a precessing magnet into an adjacent NM metal. The orbital and spin currents generated by the precession of the magnet induce a transverse voltage via the IOHE and ISHE. (c) Schematic illustration of the device and experimental setup. The RF current (*I*_RF_) flowing through the waveguide generates a time-varying RF Oersted field *h*_*z*_ on the NM/FM device. A fraction of the applied RF current is induced in the device $\left(I_{L}^{device}\right)$ flowing along the longitudinal *x*-direction. Additionally, a static magnetic field *H* is applied in the plane of the device with an angle ϕ with respect to the *x*-axis. The angle convention is chosen such that rotating in the mathematical positive direction increases the value of the angle. The voltage resulting from the orbital and/or spin pumping is measured along the sample wire.

Both spin and orbital pumping provide methods to generate spin and orbital currents without the challenges posed by electrical conductivity mismatch and enable easy and clear detection in comparatively simple structures.^41^ As illustrated in Figure 1a,b, the emitted orbital and spin currents are converted into a transverse voltage in the adjacent nonmagnet via reciprocal effects known as the inverse orbital Hall effect (IOHE), the inverse orbital Rashba-Edelstein effect^30,42–44^ and the inverse spin Hall effect (ISHE) and inverse Rashba-Edelstein effect,^45,46^ respectively. However, in real systems, these processes (IOHE, and ISHE) can occur simultaneously, along with various spin-rectification effects (SREs) arising from the interplay between time-varying magnetoresistance and the applied oscillating field. This mixing of several voltage components makes it highly challenging to isolate the orbital pumping signal from the often present background contributions of both SREs and spin pumping, and so methods are needed to identify orbital pumping unambiguously.

In this work, we demonstrate that we can obtain a clear distinction of orbital pumping by carefully selecting materials with opposing signs of the OHE and SHE and by performing rigorous angular-dependent measurements of the pumped voltage signal. This is achieved using devices specifically designed to produce a more uniform radio frequency (RF) field while minimizing other parasitic effects (Figure 1c) as discussed in subsequent sections.

We have prepared four primary series of samples using a Singulus Rotaris tool via magnetron sputtering on undoped Si/SiO_2_ substrates: (1) Sub/Ta(1)/Nb(4)/Ni­(3, 6, 10, 15)/cap, (2) Sub/Ta(1)/Pt(4)/Ni­(3, 6, 10, 15)/cap, (3) Sub/Ta(1)/Nb(4)/Fe_60_Co_20_B_20_(8)/cap, and (4) Sub/Ta(1)/Pt(4)/Fe_60_Co_20_B_20_(8)/cap. Here, the numbers in parentheses are the nominal thicknesses in nm, and the cap is MgO(1.5)/Ta(1.5) to protect the samples from oxidation. We fabricate the devices shown in Figure 1c through successive cycles of photolithography, Ar^+^ milling, RF magnetron sputtering, and lift-off techniques. More information on sample preparation and fabrication is provided in the Supporting Information (SI1).

A schematic diagram of the device is shown in Figure 1c. It consists of a coplanar waveguide with a narrow and long nonmagnetic (NM)/ ferromagnetic (FM) wire placed in the slots of the waveguide with a length of 200 μm and a width of 8 μm. The waveguide and the NM/FM device, together with the contacts, are electrically isolated by inserting an SiO_2_ layer. The central concept is to pass an RF current through the waveguide, producing an RF magnetic field that drives the magnet into resonance. As a result, the magnet emits both orbital and spin currents into the adjacent NM layer, such as Nb, Pt, or Ru, which are then converted into a direct current (DC) voltage due to the IOHE and ISHE (Figure 1a,b).

We chose Nb and Pt as they exhibit opposite signs for the spin Hall angle (SHA), yet have the same sign for the orbital Hall angle (OHA).^22^ The ferromagnetic materials Ni and FeCoB are chosen as Ni exhibits a much stronger orbital-to-spin conversion efficiencies compared to FeCoB, attributed to its stronger spin–orbit coupling.^20,22^ By Onsager reciprocity, Ni is consequently expected to generate a more pronounced orbital current via orbital pumping than FeCoB.^32–34^ The unique characteristic of Nb, exhibiting opposite signs for its SHE and OHE, combined with the markedly higher orbital-to-spin conversion efficiency in Ni compared to FeCoB, forms the basis for our experimental approach. This strategy allows us to clearly demonstrate the anticipated dependence of the pumping signal on the ferromagnetic material in Nb. For comparison, the pumping signal into Ru, another candidate known for exhibiting strong OHE and weak SHE^12,22^ is also analyzed in the Sub/Ta(1)/Ru(4)/Ni(10)/cap stack (see Supporting Information SI4).

The experiment is performed by sweeping an external magnetic field (*H*) at an angle ϕ relative to the *x*-axis, and measuring the voltage in the NM/FM wire, as shown in Figure 1c. This voltage is fitted using symmetric (*V*_S_) and antisymmetric (*V*_A_) Lorentzian functions.^38,39,45^ In the absence of other SREs, *V*_S_ corresponds to the spin/orbital pumping signal, which can be unambiguously identified via sign reversal in our measurements due to the competing sign of SHA and OHA in Nb.^22^

While the spin (and orbital) pumping effects are expected to generate only *V*_S_, previous experiments have provided significant evidence for a nonzero value of *V*_A_.^38,39,45^ This observation is often attributed to SREs, primarily arising from the induced RF current within the device (Figure 1c), which couples with the oscillating magnetoresistance as the FM oscillates at ferromagnetic resonance. Consequently, it is critical to determine whether SREs also generate *V*_S_, as this would complicate the analysis of spin and orbital pumping, potentially leading to incorrect or ambiguous interpretations. One possible origin of *V*_S_ is the conventional spin-torque ferromagnetic resonance (ST-FMR),^47^ caused by the induced RF current flowing through the device. This mechanism, which can produce a significant *V*_S_, has largely been overlooked in previous studies, particularly when the device is placed on top of the waveguide.^37–39,44,45^

To address this issue, we have fabricated the NM/FM device within the slot of a waveguide (green blue bar in Figure 1c). This configuration allows us to distinguish the pumping signal from other SREs, which is not feasible when the device is positioned on top of the waveguide, as commonly practiced in earlier studies.^48^ In our geometry, *V*_S_ exhibits the following angular dependence as a function of the in-plane magnetic field (*H*) applied at an angle, ϕ relative to the long axis of the device.

1 $$V_{S}\left(\phi \right)\approx V_{S}^{\mathrm{pump}}\sin\phi +V_{S,\mathrm{AMR}}^{\mathrm{ST}‐\mathrm{FMR}}\cos\phi \,\sin2\phi +V_{S,\mathrm{AMR}}^{\mathrm{NL}}\sin2\phi$$

In this work, we primarily focus on the coefficient $V_{S}^{\mathrm{pump}}$ in different samples, which represents the strength of the pumping signal. $V_{S,\mathrm{AMR}}^{\mathrm{ST}‐\mathrm{FMR}}$ accounts for contributions from conventional ST-FMR, arising from the induced RF current and the anisotropic magnetoresistance (AMR) effect as discussed before. $V_{S,\mathrm{AMR}}^{\mathrm{NL}}$ originates from nonlocal ST-FMR, where the induced RF current couples with out-of-plane magnetization oscillations driven by the Oersted field of the waveguide. In addition to the components included in Equation 1, there are other possible contributions from SREs to *V*_S_, which are negligible in our samples. We discuss this in the subsequent sections in more detail.

Figure 2 presents the central results of this work. Figures 2a–d show the typical voltage spectrum (black squares) in our pumping experiment, fitted with a superposition of symmetric (*V*_S_) (blue) and antisymmetric Lorentzian functions (*V*_A_) (red). The fit is displayed in green. Additionally, the fit includes a constant and linear term (all details are provided in the Supporting Information (SI2)). The sign of *V*_S_ in Pt/Ni (Figure 2c) and Pt/FeCoB (Figure 2d) reflects the widely studied spin pumping effect in Pt.^37–39,45^ Most interestingly, we observe a sign reversal in *V*_S_ for Nb/Ni (Figure 2a) compared to Nb/FeCoB (Figure 2b). This strong dependence on the FM cannot be explained by the conventional spin pumping effect. We find that the sign of *V*_S_ in Nb/FeCoB (Figure 2b) is opposite to that in Pt (Figure 2c,d), suggesting that the sign of ISHE in Nb is opposite to that in Pt, consistent with theoretical predictions^17^ and our previous work.^22^ In contrast, the observed same sign of *V*_S_ in Nb/Ni (Figure 2a) and Pt/(Ni or FeCoB) (Figures 2c,d) strongly suggests that the orbital pumping effect dominates in the Nb/Ni samples where the injected orbital current is converted into an electrical voltage via the IOHE. Note that significantly larger voltage signals are observed in FeCoB-based films compared with those in Ni-based films. We attribute this to the lower intrinsic damping of FeCoB, which results in a larger precessional cone angle of the magnetic moments.

**Figure 2:**
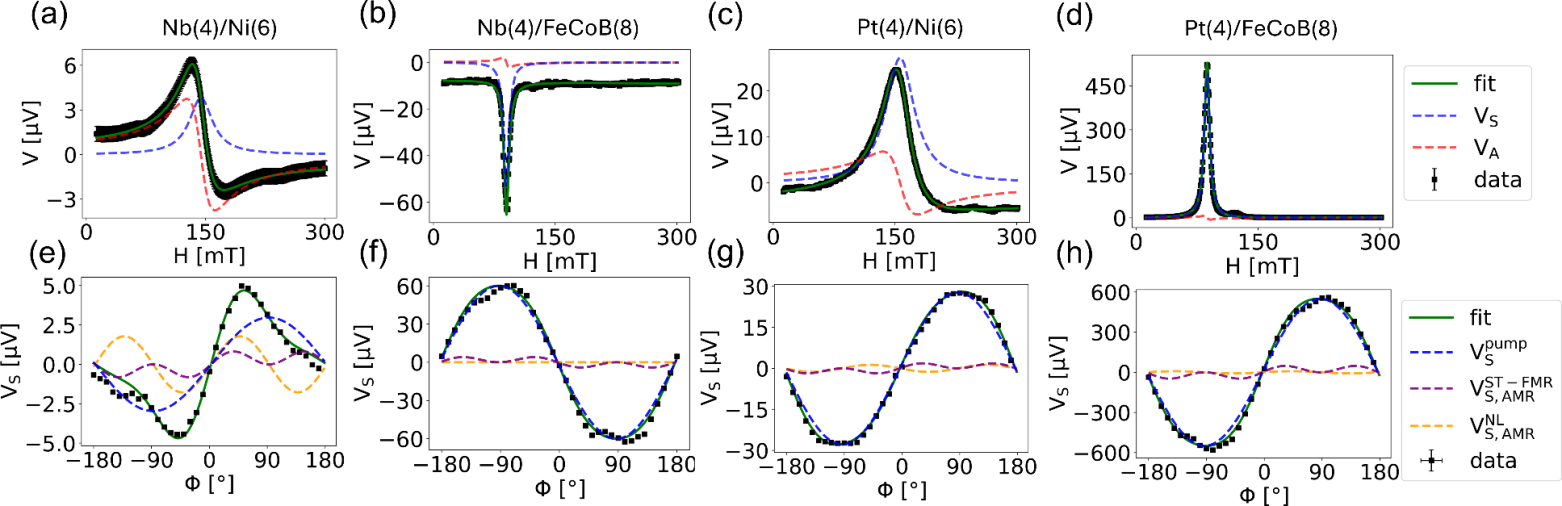
Magnetic field sweep measurements for (a) Nb(4)/Ni(6), (b) Nb(4)/FeCoB(8), (c) Pt(4)/Ni(6), and (d) Pt(4)/FeCoB(8) for a gap width of 6 μm and an angle of 80°. The data are fitted with a superposition of symmetric (blue) and antisymmetric (red) Lorentzians. (e–h) The extracted values for *V*_S_ are plotted as a function of the angle of the swept magnetic field. The data are fitted according to Equation 1.

Therefore, our results reveal a strong dependence of the sign of *V*_S_ on the ferromagnetic material in Nb/FM bilayers, with Nb/FeCoB displaying a sign consistent with conventional spin pumping, while Nb/Ni exhibits a sign reversal that points to the dominance of orbital pumping via the IOHE.

Next, we analyze the influence of other SREs on our measurements by studying the angular dependence of *V*_S_, as shown in Figures 2e–h. We observe that *V*_S_ can be reasonably well fitted with Equation 1, indicating a prominent contribution from spin and orbital pumping (sin ϕ term) along with nonzero values of other SREs across all samples. These findings confirm that the sign of the SHA is negative for Nb and it is positive for Pt (compare Figures 2f,g,h). Further, the angular dependence reveals that the pumping signal (sin ϕ term in *V*_S_) in Nb/Ni is positive (Figure 2e), indeed predominantly driven by orbital pumping prevailing over spin pumping.

Our work demonstrates that SREs are more pronounced in films incorporating Ni than in those incorporating FeCoB (compare Figure 2e with Figure 2f), as Ni exhibits the highest AMR among other transition metal magnets (Co, Fe). This enhanced AMR leads to more significant SREs from the undesired spin and orbital currents, and various current-induced magnetic fields (more details are provided in the Supporting Information SI3).

The applied RF current in the waveguide can induce both longitudinal $\left(I_{L}^{device}\right)$ and transverse $\left(I_{T}^{device}\right)$ currents through the device due to the nonzero electrical conductivity of the substrate at RF frequencies. $I_{L}^{device}$ produces $V_{S,\mathrm{AMR}}^{\mathrm{ST}‐\mathrm{FMR}}\cos\phi \,\sin2\phi$ and $V_{S,\mathrm{AMR}}^{NL}\sin2\phi$ due to SREs as discussed above. $I_{T}^{device}$ can additionally produce the angular dependence of *V*_S,PHE_ cos 2ϕ via planar Hall effect (PHE) rectification. Note that the angular dependence of these components is distinct from the spin/orbital pumping signals $\left(V_{S}^{\mathrm{pump}}\sin\phi \right)$ . The anomalous Hall effect (AHE)-induced rectification appears as an angle-independent DC offset. We confirm it is negligible in all samples and therefore omit it from our fitting. We further confirm that $I_{T}^{device}$ has a negligible effect compared with $I_{L}^{device}$ , as shown by comparing the magnitudes of all the components of *V*_S_ and *V*_A_. Hence, *V*_S,PHE_ cos 2ϕ can be neglected in Equation 1 since it arises from $I_{T}^{device}$ . A detailed discussion of the origins of these components, together with a comparison of their magnitudes, is provided in the Supporting Information (SI3).

We also conducted an analogous experiment in Ru/Ni (see Supporting Information SI4) that shows large signals from orbital pumping due to the vanishingly small SHA and the predicted large OHA.^14,22^

Building upon these observations, we investigate the dependence of the pumping effect and SREs on the gap width (*d*) between the device and the waveguide in Nb(4)/Ni(10) samples (Figure 3). Because each gap width is measured on a distinct sample, this variation tests and confirms the robustness of our results. The individual data points are obtained using Equation 1. We find that the pumping signal ( $V_{S}^{pump}$ , blue curve in Figure 3) goes down as *d* increases and is proportional to $\frac{1}{d^{m}}$ with *m* ≈ 1.0 in our work. Since the spin and orbital pumping voltage is proportional to the power of the microwave magnetic field, it is expected to scale with $\frac{1}{d^{2}}$ . However, due to the finite size of the waveguide and the device, a behavior of *m* < 2 is expected. The voltage component arising from $V_{S,AMR}^{ST-FMR}$ also follows a similar trend (purple squares in Figure 3) but exhibits a steeper decrease with *m* ≈ 2.0. Such a steep decrease in $V_{S,AMR}^{ST-FMR}$ is expected as it results from the interplay between the induced RF current and its Oersted field within the device, both of which diminish as the spacing increases. We find a similar behavior in the bilayer system Ru(4)/Ni(10) (see Supporting Information SI5). The gap dependence is thus another efficient way to separate the pumping signal from other SREs. Moreover, the systematic behaviour of both $V_{S}^{pump}$ and $V_{S,AMR}^{ST-FMR}$ , together with $V_{S}^{pump}>0$ across all measured samples, underscores the robustness of our approach and confirms the validity of orbital pumping identification in all Nb/Ni devices.

**Figure 3:**
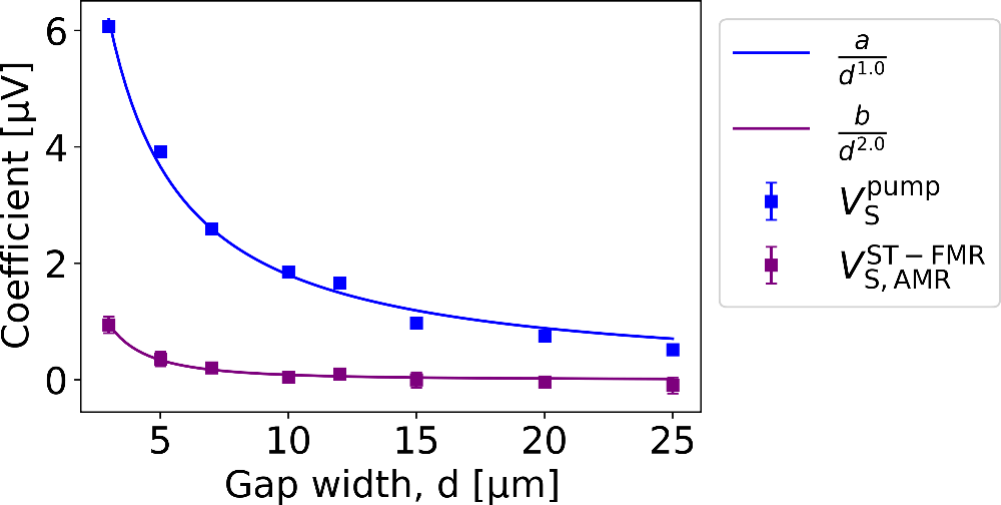
Strengths of the different effects in Nb(4)/Ni(10) bilayers are characterized by the coefficients from Equation 1. The coefficients are plotted as a function of the gap width, *d*. The $V_{S}^{pump}$ data are fitted with $\frac{a}{d^{m}}$ obtaining a value of *m* ≈ 1.0 for the best fit.

To summarize, in this work, we systematically investigate spin and orbital pumping effects in Nb/Ni and Nb/FeCoB bilayers, comparing our findings with those from Pt/Ni and Pt/FeCoB systems. We examine the DC voltage spectrum generated by orbital and spin pumping, along with other rectification effects, under uniform microwave excitation, ensured by positioning the lithographically fabricated bilayer device within the waveguide slot. Our methodology includes analyzing the angular dependence of the measured voltage signal as a function of the in-plane magnetic field, as well as its variation with the separation between the waveguide and the device. This approach enables us to uniquely distinguish the pumping signal from undesired additional rectification effect signal contributions. We confirm the orbital pumping effect by observing the sign reversal of the sin ϕ component of the symmetric Lorentzian voltage signal in Nb/Ni compared to Nb/FeCoB and its spacing dependence, which provides a distinct signature from spin rectification effects. Thus, we not only demonstrate an efficient method to generate orbital current via the ”orbital pumping” mechanism from a precessing magnet but also establish a robust approach to disentangle it from spin pumping and other rectification effects.

## Supplementary Material





## Data Availability

Data are made available from the corresponding author upon reasonable request.

## Notes

Data analysis and discussions of experimental data related to this work; a theoretical discussion of the spin rectification effects present in our samples (PDF)
(ZIP)

## $

Department of Physics, University of Gothenburg, 41296, Gothenburg, Sweden
