# Supplementary material for: Identification of Orbital Pumping from Spin Pumping and Rectification Effects
Source: Nano Lett. 2025 Aug 26;25(36):13462–7. doi: 10.1021/acs.nanolett.5c02641 (PMC12426985; doi:10.1021/acs.nanolett.5c02641)
Supplement: Supplementary file 1 [file nl5c02641_si_001.pdf]

Identification of Orbital Pumping from Spin Pumping and Rectification Effects

Nils Keller,[†,‡,@] Arnab Bose,[†,¶,@] Nozomi Soya,[‡,@] Elias Hauth,[†,‡] Fabian Kammerbauer,[†] Rahul Gupta,[†] Hiroki Hayashi,[‡] Hisanobu Kashiki,[‡] Gerhard Jakob,[†] Sachin Krishnia,[*,†] Kazuya Ando,[*,‡,§,‖] and Mathias Kläui[*,†,⊥,#]

†*Institute of Physics, Johannes Gutenberg University Mainz, Staudingerweg 7, 55128 Mainz, Germany*

‡*Department of Applied Physics and Physico-Informatics, Keio University, Yokohama 223-8522, Japan*

¶*Department of Electrical Engineering, Indian Institute of Technology Kanpur, 208016, India*

§*Keio Institute of Pure and Applied Science (KiPAS), Keio University, Yokohama 223-8522, Japan*

‖*Center for Spintronics Research Network (CSRN), Keio University, Yokohama 223-8522, Japan*

⊥*Graduate School of Excellence Materials Science in Mainz, 55099, Mainz, Germany*

#*Department of Physics, Center for Quantum Spintronics, Norwegian University of Science and Technology, 7491, Trondheim, Norway*

@*These authors contributed equally to this work.*

E-mail: krishnia@uni-mainz.de; ando@appi.keio.ac.jp; klaeui@uni-mainz.de

SI1: Sample Preparation

The samples were prepared using a Singulus Rotaris sputtering tool. The thin films were deposited on undoped Si/SiO_2 substrates with a 300 nm thermal oxide layer in an Argon atmosphere, with a partial pressure ranging from 2.5×10^{-3} mbar to 4.9×10^{-3} mbar, and a base pressure of 5×10^{-8} mbar. The basic sample structure is substrate/non-magnetic (NM) layer/NM/ferromagnetic (FM) layer/cap, where the capping consists of MgO(1.5)/Ta(1.5) to prevent oxidation. The NM materials vary between Ta(1)/Pt(4), Ta(1)/Nb(4), and Ta(1)/Ru(4), with the FM layers being either Ni or $Fe_{60}Co_{20}B_{20}$. The Ta layer facilitates better growth of Pt, Nb, and Ru, while Pt, Nb, and Ru serve as sources of spin and orbital torques, respectively.

We therefore prepared four primary series of samples on undoped Si/SiO_2 substrates using a Singulus Rotaris tool via magnetron sputtering. The sample series are as follows: (1) Sub/Ta(1)/Nb(4)/Ni with nominal thicknesses of 3, 6, 10, and 15 nm; (2) Sub/Ta(1)/Pt(4)/Ni with the same Ni thicknesses; (3) Sub/Ta(1)/Nb(4)/$Fe_{60}Co_{20}B_{20}$(8)/cap; and (4) Sub/Ta(1)/Pt(4)/$Fe_{60}Co_{20}B_{20}$(8)/cap. In these structures, the numbers in parentheses represent the nominal thicknesses in nm, and the capping layer composed of MgO(1.5)/Ta(1.5) is employed to prevent sample oxidation. Additionally, we prepared a fifth stack, Sub/Ta(1)/Ru(4)/Ni(10)/cap, to serve as a reference. The devices illustrated in Figure S1 (a, b) were fabricated through successive cycles of electron beam lithography, Ar^+ milling, radio frequency (RF) magnetron sputtering, and lift-off techniques.

SI2: Characterization Techniques

A radio frequency RF current I_{RF} is applied through a waveguide, generating an out-of-plane RF magnetic field at the position of the device placed in the waveguide slot, as illustrated in Figure S1 (a). In addition, a static in-plane magnetic field is applied to the sample location and swept from -300 mT to 300 mT. The measurements are conducted at an input power

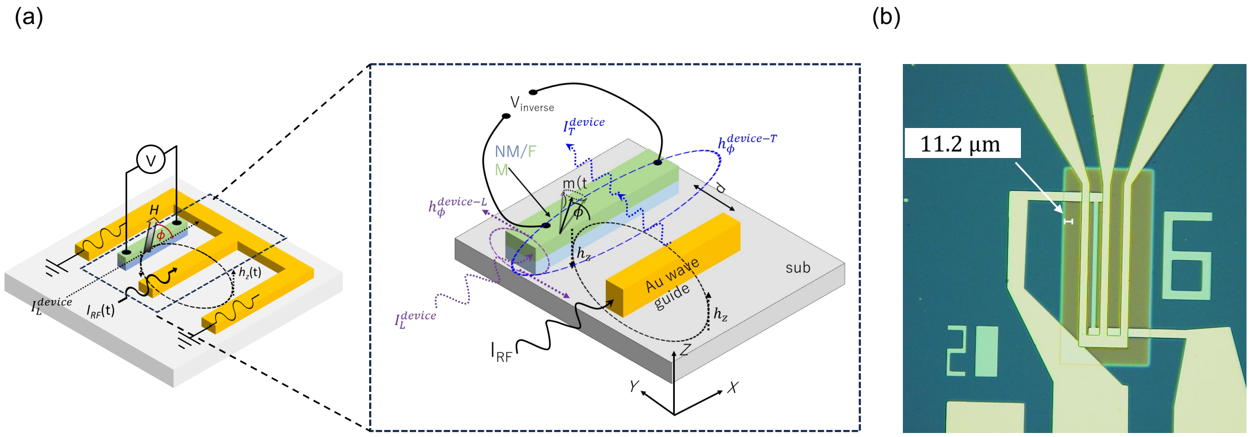

Figure S1: (a) Detailed schematic illustration of the device and experimental setup. The RF current (I_{RF}) flows through the waveguide, generating an RF Oersted field h_z within the NM/FM device. A portion of the applied RF current is induced in the device ($I_{\text{L}}^{\text{device}}$), which flows along the longitudinal x-direction. Additionally, a static magnetic field H is applied in the plane of the device, making an angle ϕ with respect to the x-axis. The time varying currents and fields cause a complicated behaviour of the magnetic moments in the FM layer. The voltage resulting from the spin and/or orbital pumping is measured along the sample wire. (b) Optical image of a fabricated device. The number 6 on the right indicates the distance from the sample wire to the waveguide in µm. The number 20 on the left is an index that uniquely identifies a specific device. A scale has been included for orientation.

of 200 mW and a frequency of $f = 10\,\text{GHz}$ at room temperature.

Due to the various components of the current-induced RF fields, the magnetization vector in the device precesses around its equilibrium axis, aligned along the applied direct current (DC) magnetic field. As the DC magnetic field is swept, a resonance condition is eventually met. Under this resonance, the magnet emits both orbital and spin currents due to orbital and spin pumping mechanisms. These emitted orbital and spin currents, when injected into an adjacent NM layer (e.g., Nb, Pt, or Ru), are converted into a transverse voltage via the inverse orbital Hall effect (IOHE) and inverse spin Hall effect (ISHE) respectively.

The NM layer has a width w, a thickness t_{NM}, and a resistance R_{NM}. The resulting pumping voltage $V_{\text{ISHE}}^{\text{SP}}$ can be expressed as

$$V_{\text{ISHE}}^{\text{SP}}(H_{\text{eff}}, \phi) = \theta_{\text{SH}} \lambda_{\text{SD}} G_{\text{eff}}^{\uparrow\downarrow} f w R_{\text{NM}} \tanh\left(\frac{t_{\text{NM}}}{2\lambda_{\text{SD}}}\right) \times e\alpha_1\beta_1 \sin\phi \frac{\Delta H^2}{(H_{\text{eff}} - H_0)^2 + \Delta H^2}, \quad (1)$$

where θ_{SH} is the effective spin Hall angle (SHA), λ_{SD} is the spin diffusion length, $G_{\mathrm{eff}}^{\uparrow\downarrow}$ is the effective spin mixing conductance, H_0 is the resonance field, e is the elementary charge, ΔH is the half-width of the ferromagnetic resonance (FMR), and α_1 and β_1 are the maximum amplitudes of the in-plane and out-of-plane precession angles of the magnetization.[1] The variable ϕ is the angle between the static effective magnetic field $\boldsymbol{H}_{\mathrm{eff}}$ and the sample bar, as shown in Figure S1. The Lorentzian line shape of the resulting voltage as a function of H_{eff} is described in detail in Ref. [1]

For the orbital pumping contribution, a similar form of the equation is expected, with the spin parameters replaced by their orbital counterparts (effective orbital Hall angle (OHA), orbital diffusion length, and effective orbital mixing conductance). The $\sin\phi$ dependence remains the same. Thus, in general, we detect a superposition of spin and orbital pumping signals.

Additionally, spin-rectification effects (SREs) are superimposed on $V_{\mathrm{ISHE}}^{\mathrm{SP}}$ and $V_{\mathrm{IOHE}}^{\mathrm{OP}}$. These SREs arise from the homodyne mixing of the induced RF current with the oscillating magnetoresistance produced by the precessing magnetization. Detailed origins of the SREs are explained in the following section.

In summary, the measured voltage is a combination of these effects and can be written as

$$V_{\mathrm{measured}}(H,\phi) \approx V_{\mathrm{S}}(\phi)\frac{\Delta H^2}{(H-H_0)^2+\Delta H^2} + V_{\mathrm{A}}(\phi)\frac{(H-H_0)\,\Delta H}{(H-H_0)^2+\Delta H^2} + V_{\mathrm{dc}} + V_{\mathrm{linear}}H. \quad (2)$$

Here, $V_{\mathrm{S}}(\phi)$ and $V_{\mathrm{A}}(\phi)$ have angular dependencies that originate from different effects and therefore differ from the simple $\sin\phi$ dependence observed in spin and orbital pumping. The constant and linear terms, V_{DC} and V_{linear}, account for instrument drift, temperature-dependent resistances, and the common Hall effect.

Finally, the angular dependences of these voltage signals are obtained by sweeping the

external magnetic field at angles ranging from 0° to 170° in steps of 10°.

SI3: Sources of Rectified Voltages in Spin/Orbital Pumping Experiments

In the presence of RF fields, various SREs arise due to the induced RF electric currents in the device. Since the waveguide is not entirely isolated at RF frequencies, a longitudinal electric current $I_{\mathrm{L}}^{\mathrm{device}}$ is induced in the device and leads to several rectification mechanisms. When $I_{\mathrm{L}}^{\mathrm{device}}$ interacts with the time-varying magnetization, anisotropic magnetoresistance (AMR) rectification can be observed. For example, an Oersted field $h_{\phi}^{\text{device-L}}$ associated with $I_{\mathrm{L}}^{\mathrm{device}}$ exerts a field-like torque on the magnetization vector m and generates an in-phase oscillation in δm_{ϕ}. This in-phase oscillation results in a time-dependent AMR that, when mixed with $I_{\mathrm{L}}^{\mathrm{device}}$, produces a characteristic DC voltage varying as $V_{\mathrm{A,AMR}}^{\text{ST-FMR}} \cos\phi \sin 2\phi$. Furthermore, the induced longitudinal spin/orbital current $J_{\mathrm{spin/orbit}}^{\text{device-L}}$ can exert a damping-like torque $(m \times (\sigma \times m))$, giving rise to an out-of-phase oscillation also in δm_{ϕ}. Mixing this out-of-phase oscillation with $I_{\mathrm{L}}^{\mathrm{device}}$ yields another DC rectified voltage, $V_{\mathrm{S,AMR}}^{\text{ST-FMR}} \cos\phi \sin 2\phi$. In addition, an out-of-phase oscillation of δm_{ϕ} caused by the $h_{z}^{\text{wave-guide}}$ field leads to time-varying AMR that, mixed with $I_{\mathrm{L}}^{\mathrm{device}}$, contributes a DC signal $V_{\mathrm{S,AMR}}^{\mathrm{NL}} \sin 2\phi$. Since the derivative of AMR with respect to δm_z vanishes, no direct AMR-based rectification contribution arises from oscillations in the z-component of the magnetization.

A transverse current $I_{\mathrm{T}}^{\mathrm{device}}$ can also be induced by the RF electric field from the waveguide, producing additional rectification effects. The Oersted field $h_{\phi}^{\text{device-T}}$ originating from $I_{\mathrm{T}}^{\mathrm{device}}$ provides a field-like torque that induces in-phase oscillations of δm_{ϕ}. This modifies the planar Hall effect (PHE), and mixing with $I_{\mathrm{T}}^{\mathrm{device}}$ yields a DC voltage $V_{\mathrm{A,PHE}} \sin\phi \cos 2\phi$.

Additionally, an out-of-phase δm_{ϕ} oscillation caused by $h_{z}^{\text{wave-guide}}$ modifies the PHE in a way that, when combined with $I_{\mathrm{T}}^{\mathrm{device}}$, leads to $V_{\mathrm{S,PHE}} \cos 2\phi$. An anomalous Hall effect (AHE)-based rectification from δm_z occurs for $I_{\mathrm{T}}^{\mathrm{device}}$ which is independent of ϕ and therefore

contributes as a constant $V_{\mathrm{S,AHE}}$. In the presence of a phase lag, δ between the induced current and the magnetization precession at resonance, the AMR rectification will result in an antisymmetric signal of the form $V^{\mathrm{PL}}_{\mathrm{A,AMR}} \sin 2\phi$.[1] In total, the angular dependence of $V_{\mathrm{S}}(\phi)$ and $V_{\mathrm{A}}(\phi)$ are therefore the following:

$$V_{\mathrm{S}}(\phi) \approx V_{\mathrm{S}}^{\mathrm{pump}} \sin\phi + V^{\mathrm{ST\text{-}FMR}}_{\mathrm{S,AMR}} \cos\phi \sin 2\phi + V^{\mathrm{NL}}_{\mathrm{S,AMR}} \sin 2\phi + V_{\mathrm{S,PHE}} \cos 2\phi + V_{\mathrm{S,AHE}} \quad (3)$$

$$V_{\mathrm{A}}(\phi) \approx V^{\mathrm{ST-FMR}}_{\mathrm{A,AMR}} \cos\phi \sin 2\phi + V_{\mathrm{A,PHE}} \sin\phi \cos 2\phi + V^{\mathrm{PL}}_{\mathrm{A,AMR}} \sin 2\phi. \quad (4)$$

Our results demonstrate that the contributions from $V_{\mathrm{S,PHE}}$ and $V_{\mathrm{S,AHE}}$ are negligible in our samples. This can be clearly observed by comparing Figure S3 with Figure S4. Specifically, Figure S3 presents the fit using five components as described by Equation (3), with the insets highlighting the negligible magnitudes of $V_{\mathrm{S,PHE}}$ and $V_{\mathrm{S,AHE}}$. Therefore, the angular dependence of V_{S} is simplified and described by the following equation in the main paper:

$$V_{\mathrm{S}}(\phi) \approx V_{\mathrm{S}}^{\mathrm{pump}} \sin\phi + V^{\mathrm{ST\text{-}FMR}}_{\mathrm{S,AMR}} \cos\phi \sin 2\phi + V^{\mathrm{NL}}_{\mathrm{S,AMR}} \sin 2\phi. \quad (5)$$

The vanishingly small contributions from $V_{\mathrm{S,PHE}}$ and $V_{\mathrm{S,AHE}}$ are not exclusive to the samples shown in Figure S3, but are observed consistently across all measured samples.

As shown in Figure S2 (a) and (c), the antisymmetric signal V_{A} is dominated by the $\sin 2\phi$ contribution due to the phase delay, in both Nb(4)/Ni(6) and Pt(4)/Ni(6) samples. Figure S2 (b) shows that $V^{\mathrm{ST\text{-}FMR}}_{\mathrm{A,AMR}}$ is about an order of magnitude stronger than $V_{\mathrm{A,PHE}}$. This suggests that SREs are primarily driven by the longitudinal induced current ($I_{\mathrm{L}}^{\mathrm{device}}$) rather than the transverse induced current ($I_{\mathrm{T}}^{\mathrm{device}}$). This is also consistent with our previous argument that coefficients related to various SREs caused by $I_{\mathrm{T}}^{\mathrm{device}}$, such as $V_{\mathrm{S,PHE}} \cos 2\phi$ are negligible.

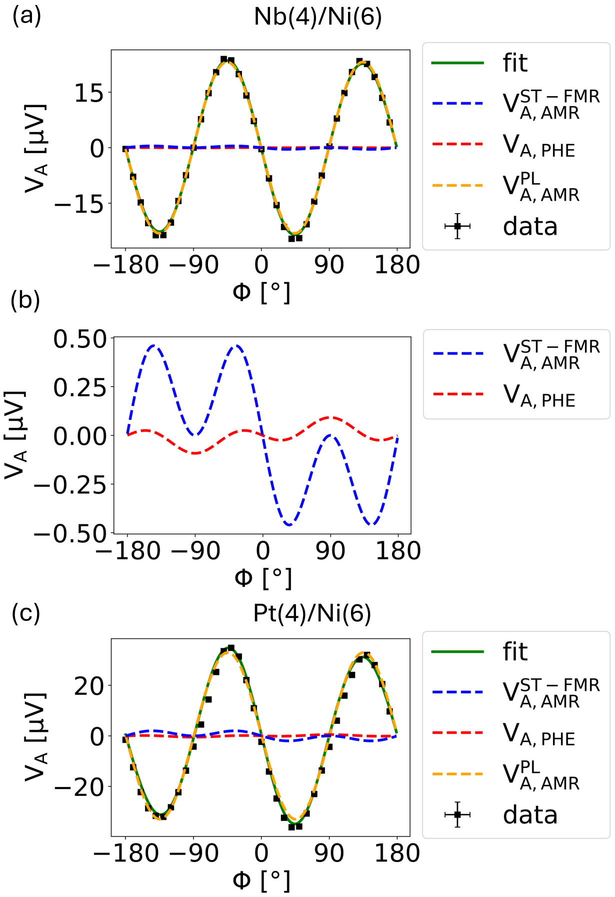

Figure S2: The antisymmetric component V_A in (a) Nb(4)/Ni(6) and (c) Pt(4)/Ni(6), extracted using Equation (2), is plotted as a function of the angle ϕ and fitted according to Equation (4), revealing a strong $\sin 2\phi$ contribution shown in orange. The two small components $V_\mathrm{A,AMR}^\mathrm{ST-FMR}$ and $V_\mathrm{A,PHE}$ from the Nb(4)/Ni(6) sample (a) are again plotted with a different scale in (b) for better visibility. The much smaller $V_\mathrm{A,PHE}$ compared to $V_\mathrm{A,AMR}^\mathrm{ST\text{-}FMR}$ indicates that spin-rectification is overwhelmingly driven by longitudinal currents, with only a minor contribution from transverse currents.

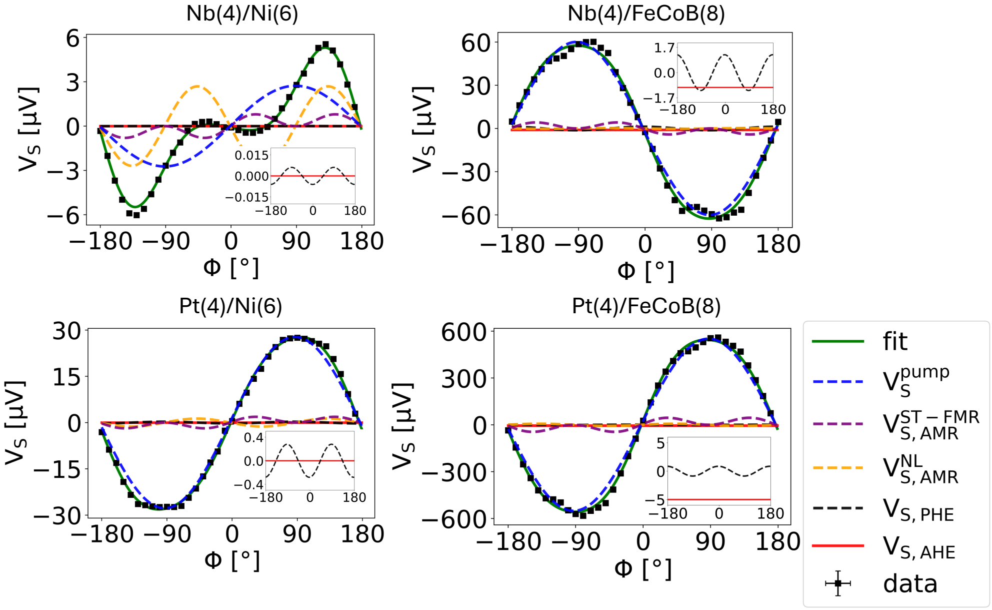

Figure S3: The coefficients of V_S extracted using Equation (2) are plotted as a function of the angle ϕ. The data are fitted using Equation (3), incorporating the five components discussed in SI3. The insets illustrate the negligible contributions of $V_\mathrm{S,PHE}$ (black) and $V_\mathrm{S,AHE}$ (red). Note that the Nb(4)/Ni(6) data are shown for a gap width of 7 µm, whereas the rest of the samples are shown for a gap width of 6 µm.

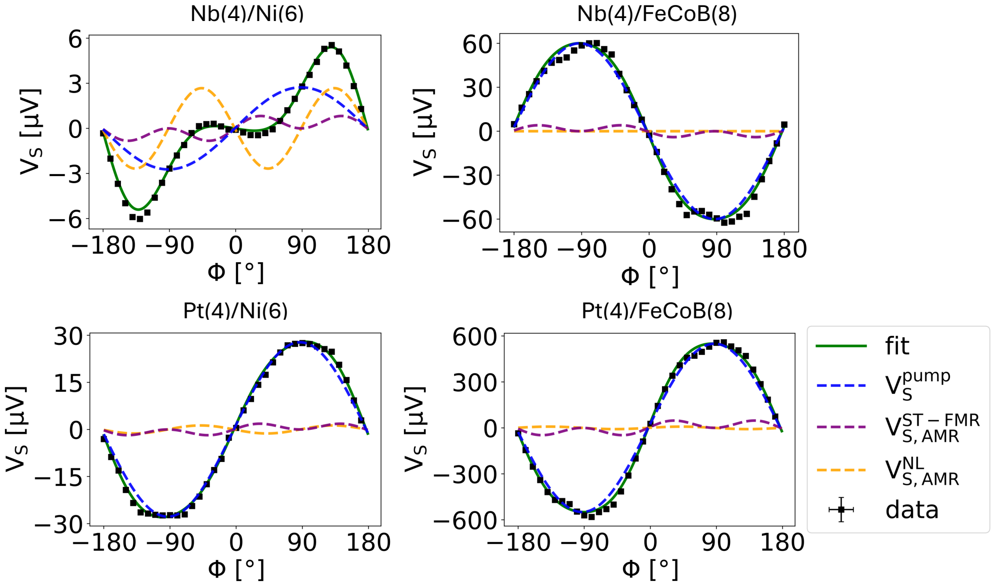

Figure S4: Values of V_S extracted from Equation (2) are plotted as a function of the angle ϕ for gap widths equivalent to those in Figure S3. The data are fitted using the three-component model described by Equation (5). The three components sufficiently capture the data behaviour, which is why they are employed in the main paper to discuss the results.

SI4: Results on Ru/Ni Samples

Even though the Ru/Ni data are not directly relevant to the observed sign change and the detection of orbital pumping, they provide evidence for the robustness of our work. The Lorentzian superposition fit (Equation (2)) is shown in Figure S5 (a). We find that the fit agrees well with our data. The angular dependence of the symmetric signal is presented in Figure S5 (b), which also matches the fit from Equation 5. Due to the vanishingly small SHA in Ruthenium and the predicted large OHA,[2,3] this signal is expected to be dominated by orbital pumping.

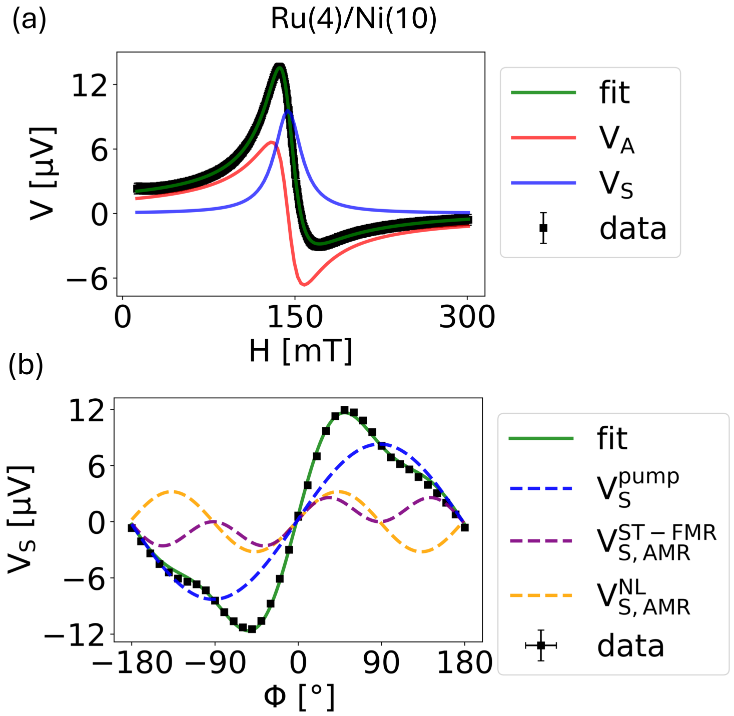

Figure S5: (a) Magnetic field sweep measurement for Ru(4)/Ni(10) for a gap width of 6 μm and an angle of 80°. The data are fitted with a superposition of symmetric (blue) and antisymmetric (red) Lorentzians. (b) The extracted values for V_S are plotted as a function of the angle of the swept magnetic field. The data are fitted according to Equation (5).

SI5: Additional Discussion on Experimental Results

In our experiments, we find that the angular dependence of V_{S} can be accurately described by Equation (5) for varying distances d between the device and the waveguide. As expected, we observe that with increasing spacing, the pumping signal $V_{\mathrm{S}}^{\mathrm{pump}}$ decreases following a $\frac{a}{d^m}$ dependence (Figure S6), where $m \approx 1.0$ for Nb/Ni (blue curve in Figure S6 (a)) and $m \approx 1.2$ for Ru/Ni (blue curve in Figure S6 (b)). A similar trend is observed in $V_{\mathrm{S,AMR}}^{\mathrm{ST\text{-}FMR}}$, which represents the contribution of spin and orbital currents due to the induced longitudinal electric current through the device ($I_{\mathrm{L}}^{\mathrm{device}}$). It is worth noting that $V_{\mathrm{S,AMR}}^{\mathrm{ST\text{-}FMR}}$ decays much faster than the pumping signal, with $m \approx 2.0$ for Nb/Ni and $m \approx 1.9$ for Ru/Ni, since the induced current and therefore the magnetoresistance both decrease with increasing d.

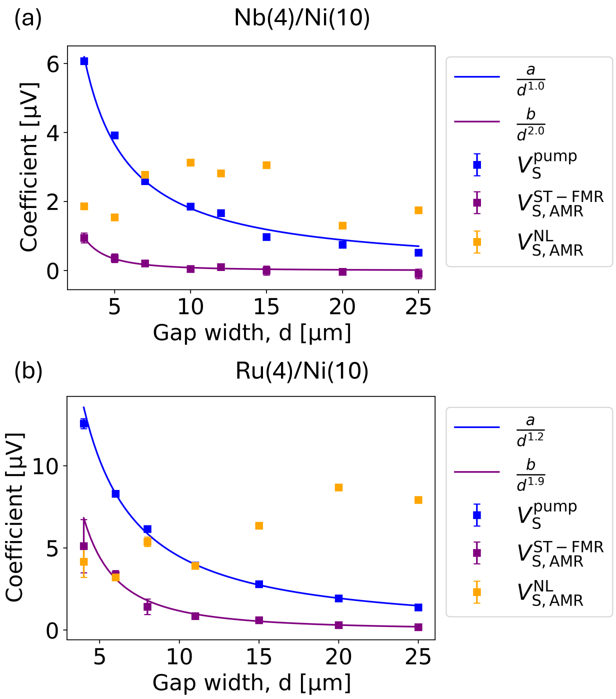

Figure S6: The strengths of the different effects in (a) Nb(4)/Ni(10) and (b) in Ru(4)/Ni(10) bilayers are characterized by the coefficients from Equation (5). The coefficients are plotted as a function of the gap width d. Surprisingly, $V_{\mathrm{S,AMR}}^{\mathrm{NL}} \sin 2\phi$ increases with increasing d for small distances d in both material combinations. $V_{\mathrm{S}}^{\mathrm{pump}}$ and $V_{\mathrm{S,AMR}}^{\mathrm{ST\text{-}FMR}}$ decrease with and expected behaviour of $\frac{a}{d^m}$.

A particularly surprising result is observed in the $V_{\mathrm{S,AMR}}^{\mathrm{NL}}$ term (yellow squares in Figure

S6), which represents the rectified signal from $I_{\mathrm{L}}^{\mathrm{device}}$ and the out-of-plane excitation (δm_z) from the Oersted field of the waveguide. Contrary to expectations, this signal increases with greater spacing. This counterintuitive result is attributed to the enhanced out-of-plane projection of the Oersted field, caused by the improved homogeneity of the RF field distribution as the spacing increases.

Finally, we summarize the three fit prefactors from Equation (5) (Equation 1 in the main paper) for all samples in Table 1.

Table 1: Summary of fit prefactors obtained from Equation (5) in µV. The first block shows the values for the main result of the paper. The second and third blocks show the values for the samples used for the gap dependence. The last block shows the additional data recorded in the Nb(4)/Ni(6) sample for a gap width of 7 µm.

Sample	$V_{\mathrm{S}}^{\mathrm{pump}}$	$V_{\mathrm{S,AMR}}^{\mathrm{NL}}$	$V_{\mathrm{S,AMR}}^{\mathrm{ST\text{-}FMR}}$
Nb(4)/Ni(6) 6 µm	2.97 ± 0.05	1.78 ± 0.15	1.05 ± 0.24
Nb(4)/FeCoB(8) 6 µm	-60.1 ± 1.3	0.01 ± 0.86	-5.3 ± 1.6
Pt(4)/Ni(6) 6 µm	27.78 ± 0.25	-1.27 ± 0.27	2.38 ± 0.54
Pt(4)/FeCoB(8) 6 µm	549.5 ± 8.7	8.1 ± 4.4	61.8 ± 8.3
Nb(4)/Ni(10) 10 µm	1.85 ± 0.05	3.13 ± 0.04	0.04 ± 0.08
Nb(4)/Ni(10) 12 µm	1.66 ± 0.04	2.82 ± 0.04	0.10 ± 0.07
Nb(4)/Ni(10) 15 µm	0.97 ± 0.07	3.05 ± 0.07	0.00 ± 0.13
Nb(4)/Ni(10) 20 µm	0.75 ± 0.01	1.30 ± 0.02	-0.04 ± 0.03
Nb(4)/Ni(10) 25 µm	0.52 ± 0.09	1.75 ± 0.07	-0.10 ± 0.14
Nb(4)/Ni(10) 3 µm	6.07 ± 0.07	1.86 ± 0.08	0.94 ± 0.14
Nb(4)/Ni(10) 5 µm	3.92 ± 0.06	1.54 ± 0.07	0.36 ± 0.12
Nb(4)/Ni(10) 7 µm	2.59 ± 0.05	2.78 ± 0.04	0.21 ± 0.08
Ru(4)/Ni(10) 15 µm	2.79 ± 0.03	6.35 ± 0.07	0.60 ± 0.11
Ru(4)/Ni(10) 11 µm	3.93 ± 0.05	3.90 ± 0.11	0.84 ± 0.18
Ru(4)/Ni(10) 4um	12.56 ± 0.30	4.2 ± 1.0	5.1 ± 1.6
Ru(4)/Ni(10) 20 µm	1.93 ± 0.02	8.68 ± 0.05	0.30 ± 0.08
Ru(4)/Ni(10) 25 µm	1.38 ± 0.03	7.92 ± 0.06	0.17 ± 0.09
Ru(4)/Ni(10) 8 µm	6.15 ± 0.08	5.37 ± 0.28	1.42 ± 0.47
Ru(4)/Ni(10) 6 µm	8.29 ± 0.06	3.21 ± 0.16	3.35 ± 0.27
Nb(4)/Ni(6) 7 µm	2.73 ± 0.02	-2.67 ± 0.08	1.07 ± 0.15

References

(1) Feng, Z.; Hu, J.; Sun, L.; You, B.; Wu, D.; Du, J.; Zhang, W.; Hu, A.; Yang, Y.; Tang, D. M.; Zhang, B. S.; Ding, H. F. Spin Hall angle quantification from spin pumping and microwave photoresistance. *Phys. Rev. B* **2012**, *85*, 214423.

(2) Bose, A.; Kammerbauer, F.; Gupta, R.; Go, D.; Mokrousov, Y.; Jakob, G.; Kläui, M. Detection of long-range orbital-Hall torques. *Phys. Rev. B* **2023**, *107*, 134423.

(3) Gupta, R.; Bouard, C.; Kammerbauer, F.; Ledesma-Martin, J. O.; Bose, A.; Kononenko, I.; Martin, S.; Usé, P.; Jakob, G.; Drouard, M.; others Harnessing orbital Hall effect in spin-orbit torque MRAM. *Nat. Commun.* **2025**, *16*, 130.
