# Supplementary material for: Identification of Orbital Pumping from Spin Pumping and Rectification Effects
Source: Nano Lett. 2025 Aug 26;25(36):13462–7. doi: 10.1021/acs.nanolett.5c02641 (PMC12426985; doi:10.1021/acs.nanolett.5c02641)
Supplement: Supplementary file 2 [file nl5c02641_si_002.zip › SM1withopticalimage.pdf]

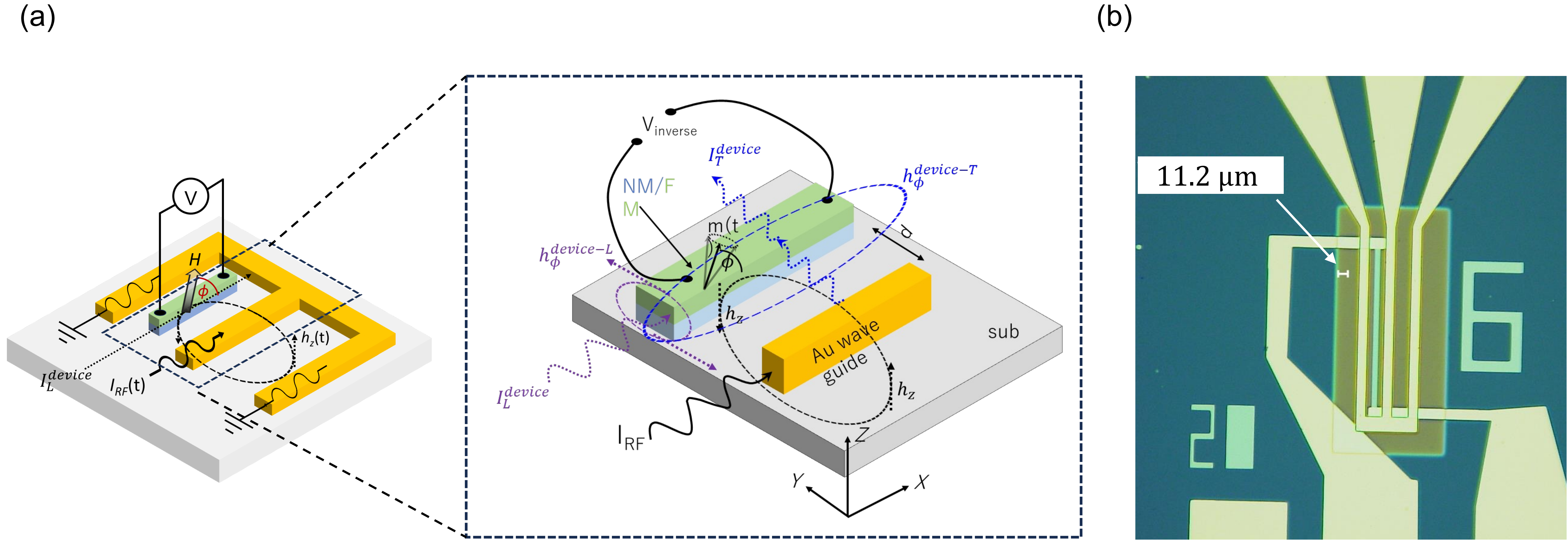

(a)
(b)
V
H
ϕ
I_L^{device}
I_{RF}(t)
h_z(t)
V inverse
I_T^{device}
$h_\phi^{device-T}$
NM/F
M
m(t
ϕ
$h_\phi^{device-L}$
d
h_z
Au wave guide
sub
h_z
I_L^{device}
I_{RF}
Z
Y
X
11.2 μm
21
6
