# Supplementary material for: Identification of Orbital Pumping from Spin Pumping and Rectification Effects
Source: Nano Lett. 2025 Aug 26;25(36):13462–7. doi: 10.1021/acs.nanolett.5c02641 (PMC12426985; doi:10.1021/acs.nanolett.5c02641)
Supplement: Supplementary file 2 [file nl5c02641_si_002.zip › achemso/achemso.pdf]

achemso — Support for submissions to American Chemical Society journals*

Joseph Wright†

Released 2025-04-11

Abstract

The achemso bundle provides a LaTeX class file and BibTeX style file in accordance with the requirements of the American Chemical Society (ACS). The files can be used for any documents, but have been carefully designed and tested to be suitable for submission to ACS journals.

The bundle also includes the natmove package. This package is loaded by achemso, and provides automatic moving of superscript citations after punctuation.

Contents

1 Introduction 1

2 Installation 2

3 Requirements 2

4 The class file 3
- 4.1 Class options 3
- 4.2 Manuscript meta-data . 4
- 4.3 Floats 5
- 4.4 Section headers 5
- 4.5 Special sections 5
- 4.6 Miscellaneous commands 6

5 The package file 6
- 5.1 Package options 6
- 5.2 Bibliography notes . . . 7

6 The BibTeX style files 7

7 The natmove package 8

8 Implementation 8
- 8.1 Early class-only code . . 9
- 8.2 Early package-only code 9
- 8.3 Common code 9
- 8.4 Late class-only code . . 16
- 8.5 Late shared code 40
- 8.6 Late package-only code 41
- 8.7 Moving citations with natbib 41
- 8.8 The configuration files . 44

9 Index 59

10 References 68

1 Introduction

Support for BibTeX bibliography following the requirements of the American Chemical Society (ACS), along with a package to make these easy to have been

*This file describes version v3.13j, last revised 2025-04-11.

†E-mail: joseph.wright@morningstar2.co.uk

available since version one of achemso. The re-write from version 1 to version 2 made a number of improvements to the package, and also added a number of new features. However, neither version one nor version two of the package was targeted directly at use for submissions to ACS journals. This new release of achemso addresses this issue.

The bundle consists of four parts. The first is a LaTeX 2ε class, intended for use in submissions. It is based on the standard article class, but makes various changes to facilitate ease of use. The second part is the LaTeX package. The package contains the parts of the bundle which are appropriate for use with other document classes.[1] Thirdly, two BibTeX style files are included. These are used by both the class and the package, but can be used directly if desired. Finally, an example document is included; this is intended to act a potential template for submission, and illustrates the use of the class file.

2 Installation

The package is supplied in `dtx` format and as a pre-extracted zip file, `achemso.tds.zip`. The later is most convenient for most users: simply unzip this in your local texmf directory and run `texhash` to update the database of file locations. If you want to unpack the `dtx` yourself, running `tex achemso.dtx` will extract the package whereas `latex achemso.dtx` will extract it and also typeset the documentation.

Typesetting the documentation requires a number of packages in addition to those needed to use the package. This is mainly because of the number of demonstration items included in the text. To compile the documentation without error, you will need the packages:

- array
- booktabs
- hypdoc
- listings
- lmodern
- mathpazo
- microtype

3 Requirements

The achemso class requires the following packages:

- caption
- float
- geometry

[1] For example, when writing a thesis.

- natbib
- setspace
- xkeyval

These are normally present in the current major TEX distributions, but are also available from The Comprehensive TeX Archive Network.

4 The class file

The class file has been designed for use in submitting journals to the ACS. It uses all of the modifications described here (those in the package as well as those in the class). The accompanying example manuscript can be used as a template for the correct use of the class file. It is intended to act as a model for submission.

When submitting communications to *J. Am. Chem. Soc.*, the class will automatically lay the document out in the publication style. This allows the author to judge the length of text submitted more accurately. Changing the `manuscript` in the demonstration document to `communication` will illustrate the effect.

4.1 Class options

`journal` (*env.*) The class supports a limited number of options, which are specifically-targeted at submission. The class uses the keyval system for options, in the form `key=value`. The most important option is `journal`. This is an identifier for the target journal: from `https://pubs.acs.org/`, the identifier is the part of the URL after `https://pubs.acs.org/journal/`, *e.g.* for *J. Org. Chem.* it would be `joceah`. If an unknown journal is specified, the package will fall-back on the `journal=jacsat` option.

`manuscript` (*env.*) The second option is the `manuscript` option. This specifies the type of paper in the manuscript. The values here are `article`, `note`, `communication`, `review`, `letter` and `perspective`. The valid values will depend on the value of `journal`. The `manuscript` option determines whether sections and an abstract are valid. The value `suppinfo` is also available for supporting information.

`layout` (*env.*) The achemso class can produce drafts in two layout styles. The standard setting for the `layout` option is `traditional`, which produces a double-spaced single column manuscript. The alternative setting `twocolumn` will use single spacing and print the text in two columns. The second option is obviously more compact. If the journal requires a particular style this option may be ignored.

`email` (*env.*) It may be desirable to omit e-mail addresses from the front page of a manuscript. The printing of e-mail addresses can be disabled using the `email` option, which takes Boolean values only. The default is to print e-mail addresses. Notice that phone and fax numbers are only printed if e-mail addresses are printed.

Other options are provided by the package, but when used with the class these are silently ignored. If you need to override the settings chosen by the class, include the settings *after* the `\documentclass` line using `\setkeys`:

```
\documentclass[journal = jacsat]{achemso}
\setkeys{acs}{articletitle = false}
```

4.2 Manuscript meta-data

\author \affiliation \alsoaffiliation \altaffiliation \email Inspired by REVTEX, the achemso class alters the method for adding author information to the manuscript. Each author should be given as a separate \author command. These should be followed by an \affiliation, which applies to the preceding authors. The \affiliation macro takes an optional argument, for a short version of the affiliation.[2] At least one author should be followed by an \email macro, containing contact details. All authors with an e-mail address are automatically marked with a star. The example manuscript demonstrates the use of all of these macros. Notice that \alsoaffiliation is used when one (or more) authors work at multiple institutions, while \altaffiliation is intended for previous addresses (or other notes). Only \affiliation applies to multiple authors: both \alsoaffiliation and \altaffiliation are set on a per author basis.

```
\author{Author Person}
\author{Second Bloke}
\email{second.bloke@some.place}
\affiliation[University of Sometown]
  {University of Somewhere, Sometown, USA}
\altaffiliation
  {Previous address: Minute University, Nowhereville, USA}
\author{Indus Trialguy}
\email{i.trialguy@sponsor.co}
\affiliation[SponsoCo]
  {Research Department, SponsorCo, BigCity, USA}
\alsoaffiliation{University of Somewhere, Sometown, USA}
```

Repeated entries for \affiliation and \alsoaffiliation will result in only one address being printed in the address list and footnotes. Repeated \altaffiliation entries only produce a single footnote, and can therefore be used for entries such as

```
\author{First Coworker}
\altaffiliation{Contributed equally to this work}
\author{Second Coworker}
\altaffiliation{Contributed equally to this work}
```

if required.

\fax \phone The class will recognise the optional information \fax and \phone, which will be printed along with the lead authors e-mail address. Note that this information is only used for authors who have an e-mail address supplied.

```
\author{Second Bloke}
\email{second.bloke@some.place}
\phone{+xxx (0)yyy zzzzzz}
\fax{+xxx (0)yyy wwwwww}
\affiliation[University of Sometown]
  {University of Somewhere, Sometown, USA}
```

\and \thanks The method used for setting the meta-data means that the normal \and and \thanks macros are not appropriate in the pkgachemso class. Both produce a warning if used.

\title The \title macro is extended to accept an optional argument. This is

[2]This will usually be the university or company name.

intended for a shortened version of the journal title suitable for running headers. Some journals require that authors supply this data: if it is needed then it will be printed in the appropriate place.

```
\title[Short running title]
  {Long title which would not fit in running headers}
```

The meta-data items should be given in the preamble to the LaTeX file, and no `\maketitle` macro is required in the document body. This is all handled by the class file directly. At least one author, affiliation and e-mail address must be specified.

4.3 Floats

`scheme` (*env.*)
`chart` (*env.*)
`graph` (*env.*)

The class defines three new floating environments: `scheme`, `chart` and `graph`. These can be used as expected to include graphical content. The placement of these new floats and the standard `table` and `figure` floats is altered to be "here" if possible. The contents of all floats is automatically horizontally centred on the page.

4.4 Section headers

`\SectionNumbersOff`
`\SectionNumbersOn`

Some journals have no section numbering by default. This can be set up in the appropriate configuration file, but it may be that individual users need to change the decision. The macros `\SectionNumbersOff` and `\SectionNumbersOn` are therefore available: these should be given in the preamble.

`\SectionsOff`
`\SectionsOn`
`\AbstractOff`
`\AbstractOn`

More radically, the entire availability of sections can be turned on and of. This is functionality is available to the user *via* the `\SectionsOn` and `\SectionsOff` macros, which again are preamble-only. Similar functions are available for the abstract: `\AbstractOff` and `\AbstractOn`.

4.5 Special sections

`acknowledgement` (*env.*)
`suppinfo` (*env.*)

The sections for acknowledgements and supporting information have dedicated environments available. These ensure that the section headings are generated, and that the text is sized corrected when using creating a Communication.

```
\begin{acknowledgement}
  The authors thank A.N.~Other.
\end{acknowledgement}

\begin{suppinfo}
  Full characterization data for all new compounds.
\end{suppinfo}
```

`tocentry` (*env.*)

For generating an entry for the graphical table of content, required by some journals, the environment `tocentry` is available. This prints its content in an appropriately sized box on a separate page. In contrast to the rest of the manuscript, this section is intended to be "print ready" in appearance.

```
\begin{tocentry}
  \includegraphics{toc-entry-graphic}
  Some text to explain the graphic.
\end{tocentry}
```

4.6 Miscellaneous commands

\latin

The command \latin is provided by the class to format Latin phrases such as "et al." Most ACS journals do not make these italic, but some (for example *ACS Nano*) do. By providing a command, the formatting is left flexible.

\doi

The bibliography style prints any DOI values as the argument to the command \doi. The default definition will allow printing of special characters but does not create hyperlinks. A more sophisticated version of the command may be set up if hyperref is loaded, for example

```
\begin{tocentry}
  \newcommand{\doi}[1]{\href{http://dx.doi.org/#1}{\nolinkurl{#1}}}
\end{tocentry}
```

5 The package file

The achemso package is independent of the class file, and contains parts of the bundle useful outside of submission to the ACS.

5.1 Package options

As with the class options, the package uses the key–value method for option set up. These are used to control the output of citations and bibliographic data. The same options are used when creating journal configurations for the class: this is a task most users will not need to undertake!

super (*env.*) The super option affects the handling of superscript reference markers. The option switches this behaviour on and off (and takes Boolean values: super=true and super=false are valid).

articletitle (*env.*) The articletitle option is a Boolean, and sets whether the title of a paper referenced appears in the bibliography. The default is articletitle=true.

doi (*env.*) The boolean doi option is provided to allow a DOI (Digital Object Identifier) to be included for bibliography entries even where other identification such as page numbers is available. The standard setting is false: setting it to true will cause DOI numbers to be printed if available.

chaptertitle (*env.*) The boolean chaptertitle option is provided to allow flexibility for the inclusion of chapter titles for book and related entries. The standard setting is false: setting it to true will cause chapter titles to be included.

etalmode (*env.*)
maxauthors (*env.*) Many journals require that long lists of authors are shortened using 'et al.' in the references section. The behaviour of the BibTeX styles in this regard is controlled by two options, etalmode and maxauthors. There are two possible ways to shorten a long list of authors. Some journals require that only the first author is given, followed by 'et al.': for this behaviour, set etalmode=firstonly:

```
Jones, A.N. et al.
```

On the other hand, some journals request that the list of authors is truncated after *n* authors. This is set up by the etalmode=truncate option:

```
Jones, A.N.; Bloggs, F.; Nobacon, D. et al.
```

In both cases, the maximum number of authors permitted before introducing 'et al.' is governed by the maxauthors option. This option recognises the sentinel value 0, which indicates that no shortening should occur at all.

biblabel (*env.*) Redefining the formatting of the numbers used in the bibliography usually requires modifying internal LaTeX macros. The biblabel option makes these changes more accessible: valid values are plain (use the number only), brackets (surround the number in brackets) and period or fullstop (follow the number by a full stop/period).

biochem (*env.*)
biochemistry (*env.*) Most ACS journals use the same bibliography style, with the only variation being the inclusion of article titles. However, a small number of journals use a rather different style; the journal *Biochemistry* is probably the most prominent. The biochemistry or biochem option uses the style of *Biochemistry* for the bibliography, rather than the normal ACS style.

5.2 Bibliography notes

\bibnote
\bibnotemark
\bibnotetext

achemso provides the \bibnote macro. This is intended for addition of notes to the bibliography (references). The macro accepts a single argument, which is transferred to the bibliography by BibTeX. In analogy to \footnote, the macros \bibnotemark and \bibnotetext are available for dividing up the marker for a note from the text.

```
Some text \bibnote{This note text will be in the bibliography}.
```

Some text.[1]

The functionality for bibnotes in achemso is based on that in the notes2bib package. The notes2bib package can be loaded with the achemso package, and no clash will occur. With the class file, notes2bib will be ignored if requested, to prevent issues on submission to the ACS.

6 The BibTeX style files

achemso is supplied with two style files, achemso.bst and biochem.bst. The direct use of these without the achemso package file is not recommended, but is possible. The style files can be loaded in the usual way, with a \bibliographystyle command. The natbib package must be loaded by the LaTeX file concerned, if the achemso package is not in use.

The style files are designed to use the mciteplus package if it is available, but to work even if is not. When mciteplus is present, it is possible to automatically produce references of the form

> (5) (a) Arduengo, A. J., III; Dias, H. V. R.; Harlow, R. L.; Kline, M. *J. Am. Chem. Soc.* **1992**, *114*, 5530–5534; (b) Appelhans, L. N.; Zuccaccia, D.; Kovacevic, A.; Chianese, A. R.; Miecznikowski, J. R.; Macchioni, A.; Clot, E.; Eisenstein, O.; Crabtree, R. H. *J. Am. Chem. Soc.* **2005**, *127*, 16299–16311; (c) Arduengo, A. J., III; Gamper, S. F.; Calabrese, J. C.; Davidson, F. *J. Am. Chem. Soc.* **1994**, *116*, 4391–4394.

as demonstrated in the example document. When mciteplus is not present, this functionality is not available but the style files will work normally.

The BibTeX style files implement the bibliographic style specified by the ACS in *The ACS Style Guide*.[2] By default, article titles are not included in output using the `achemso.bst` file, but are with the `biochem.bst` file.

The style used by the ACS does not differentiate between BibTeX `book`, `inbook`, `collection` and `incollection` entries. As a result, the appearance of these entry types depends on the fields available. Named subdivisions of a book (for example, chapters where each has a named author) should be given in the `title` field, with the title of the book itself in the `booktitle` field. The `chapter` field should be used for a chapter number, and is printed as part of the pagination.

One frequently asked question is why some people see an empty first item in their bibliography when using the achemso package or class. This is usually because they have downloaded the LaTeX files and done a local installation without also updating the BibTeX style. The two must be from the same version of achemso: they are designed to work together.

7 The natmove package

The natmove package does only one job. It brings the ability to move punctuation after citations, using code borrowed from the cite package. Both the achemso class and package load natmove automatically.

```
Some text \cite{Coghill2006} some more text.\\
Some text ending a sentence \cite{Coghill2006}.
```

Some text[2] some more text.
Some text ending a sentence.[2]

This is deactivated for other citation types.

```
Some text \citeyear{Coghill2006}.\\
Some text \citeauthor{Coghill2006}.\\
Some text \citenum{Coghill2006}.
```

Some text 2006.
Some text Coghill and Garson.
Some text 2.

The package does nothing if the `super` option has not been given to natbib. This means that the source can be written without needing to decide where citations will to appear, with the `super` option for natbib controlling the result.

`\natmovechars` One user macro is provided: `\natmovechars`. This contains the characters which are moved before superscript punctuation. The default contents is `,;:.` and can be set using `\renewcommand*`:

Some text[2], more text.[2]

```
\renewcommand*{\natmovechars}{.}
Some text \cite{Coghill2006},
more text \cite{Coghill2006}.
```

8 Implementation

A lot of the work done by the package is also needed by the class. Loading the package and the class makes load-order awkward. Instead, the two parts are done in one place. Sandwiching the common code are two slices of dedicated

material for the class and the package. Some of this is needed "early", before the common material, whilst the rest is "late".

8.1 Early class-only code

So that there is no confusion, the base class is loaded early.

```
⟨*class⟩
\ProvidesClass{achemso}
  [2025-04-11 v3.13j Submission to ACS journals]
\LoadClass[12pt,letter]{article}
```

\acs@warning The code for a warning is created so that it works for the package too.

```
\newcommand*\acs@warning{\ClassWarning{achemso}}
⟨/class⟩
```

8.2 Early package-only code

The package and the class should not both be loaded, as the two use the same internal macro names. On the other hand, if the class is not in use a reminder is printed to use it if possible.

```
⟨*package⟩
\ProvidesPackage{achemso}
  [2025-04-11 v3.13j Support for ACS journals]
\@ifclassloaded{achemso}{%
  \PackageInfo{achemso}{%
    You have already loaded the ‘achemso’ class:\MessageBreak
    loading the package will abort%
  }%
  \endinput
}{%
  \PackageInfo{achemso}{%
    When writing a submission to an ACS journal, please\MessageBreak
    use the achemso document class%
  }%
}
```

\acs@warning The code for a warning is created so that it works for the class too.

```
\newcommand*\acs@warning{\PackageWarning{achemso}}
⟨/package⟩
```

8.3 Common code

\acs@ifundefined A non-expandable test for defined macros: does not add to the hash table.

```
⟨*package | class⟩
\newcommand*\acs@ifundefined[1]{%
  \begingroup\expandafter\expandafter\expandafter\endgroup
  \expandafter\ifx\csname #1\endcsname\relax
    \expandafter\@firstoftwo
  \else
    \expandafter\@secondoftwo
  \fi
}
```

The first stage needed is to read the package options given. Although xkeyval was perhaps not the best choice, changing this now would be rather risky.

```
\RequirePackage{xkeyval}
```

\acs@keyval@bool

A support macro for making Boolean options: the xkeyval version is only available in newer releases.

```
\newcommand*\acs@keyval@bool[2]{%
  \acs@ifundefined{acs@#1#2}{%
    \acs@warning{Unknown option ‘#2’ for key #1}%
  }{%
    \@nameuse{acs@#1#2}%
  }%
}
```

\ifacs@abbreviations
\ifacs@articletitle
\ifacs@biochem
\ifacs@chaptertitle
\ifacs@doi
\ifacs@email
\ifacs@hyperref
\ifacs@keywords
\ifacs@super

These are all very trivial definitions: to avoid issues with older versions of xkeyval each definition is done directly.

```
\newif\ifacs@abbreviations
\newif\ifacs@articletitle
\newif\ifacs@biochem
\newif\ifacs@doi
\newif\ifacs@chaptertitle
\newif\ifacs@email
\newif\ifacs@hyperref
\newif\ifacs@keywords
\newif\ifacs@super
\define@key{acs}{abbreviations}[true]{%
  \acs@keyval@bool{abbreviations}{#1}%
}
\define@key{acs}{articletitle}[true]{%
  \acs@keyval@bool{articletitle}{#1}%
}
\define@key{acs}{biochem}[true]{%
  \acs@keyval@bool{biochem}{#1}%
}
\define@key{acs}{doi}[true]{%
  \acs@keyval@bool{doi}{#1}%
}
\define@key{acs}{chaptertitle}[true]{%
  \acs@keyval@bool{chaptertitle}{#1}%
}
\define@key{acs}{email}[true]{%
  \acs@keyval@bool{email}{#1}%
}
\define@key{acs}{hyperref}[true]{%
  \acs@keyval@bool{hyperref}{#1}%
}
\define@key{acs}{keywords}[true]{%
  \acs@keyval@bool{keywords}{#1}%
}
\define@key{acs}{super}[true]{%
  \acs@keyval@bool{super}{#1}%
}
\define@key{acs}{usetitle}[true]{%
```

```
  \acs@keyval@bool{articletitle}{#1}%
}
```

\acs@journal \acs@layout \acs@manuscript \acs@maxauthors

Trivial again: done without using xkeyval for the same reasons as before.

```
\newcommand*\acs@journal{jacsat}
\newcommand*\acs@layout{traditional}
\newcommand*\acs@manuscript{article}
\newcommand*\acs@maxauthors{15}
\define@key{acs}{journal}{%
  \def\acs@journal{#1}%
}
\define@key{acs}{layout}{%
  \def\acs@layout{#1}%
}
\define@key{acs}{manuscript}{%
  \def\acs@manuscript{#1}%
}
\define@key{acs}{maxauthors}{%
  \def\acs@maxauthors{#1}%
}
```

\ifacs@etal@truncate \acs@etal@firstonly \acs@etal@truncate

The setup for the etalmode option is quite simple: just look for the appropriate macros.

```
\newif\ifacs@etal@truncate
\define@key{acs}{etalmode}{%
  \acs@ifundefined{acs@etal@#1}{%
    \acs@warning{%
      Unknown value ‘#1’ for\MessageBreak  etalmode option%
    }%
  }{%
    \@nameuse{acs@etal@#1}%
  }%
}
\newcommand*\acs@etal@firstonly{\acs@etal@truncatefalse}
\newcommand*\acs@etal@truncate{\acs@etal@truncatetrue}
```

\acs@activate@biblabel

The biochemistry option is an alias for biochem. The biblabel option is a choice, which is implemented using a csname search. The group here prevents hash table pollution, whilst the xkeyval method is avoided as it is more complex than it is worth!

```
\define@key{acs}{biochemistry}{%
  \setkeys{acs}{biochem = #1}%
}
\define@key{acs}{biblabel}{%
  \acs@ifundefined{acs@biblabel@#1}{%
    \acs@warning{%
      Unknown value ‘#1’ for\MessageBreak biblabel option%
    }%
  }{%
    \acs@activate@biblabel{\@nameuse{acs@biblabel@#1}}%
  }%
}
\newcommand*\acs@activate@biblabel{}
```

```
⟨*class⟩
\let\acs@activate@biblabel\AtEndOfClass
\AtEndOfClass{\let\acs@activate@biblabel\@firstofone}
⟨/class⟩
⟨*package⟩
\let\acs@activate@biblabel\AtEndOfPackage
\AtEndOfPackage{\let\acs@activate@biblabel\@firstofone}
⟨/package⟩
```

`\acs@biblabel@brackets` `\acs@biblabel@fullstop` `\acs@biblabel@period` `\acs@biblabel@plain`

The macros to implement the idea above for biblabels.

```
\newcommand*\acs@biblabel@brackets{\def\bibnumfmt##1{(##1)}}
\newcommand*\acs@biblabel@fullstop{\def\bibnumfmt##1{##1.}}
\newcommand*\acs@biblabel@period{\def\bibnumfmt##1{##1.}}
\newcommand*\acs@biblabel@plain{\def\bibnumfmt##1{##1}}
```

Set up some defaults.

```
\setkeys{acs}{
  email = true,
  super = true
}
```

Loading some other packages depends on the options chosen, so they are processed now.

```
\ProcessOptionsX*<acs>
```

`\acs@manuscript@communication` `\acs@manuscript@letter` `\acs@manuscript@note` `\acs@manuscript@review` `\acs@manuscript@suppinfo`

For text comparisons.

```
\newcommand*\acs@manuscript@communication{communication}
\newcommand*\acs@manuscript@letter{letter}
\newcommand*\acs@manuscript@note{note}
\newcommand*\acs@manuscript@review{review}
\newcommand*\acs@manuscript@suppinfo{suppinfo}
```

`\acs@niib@create` `bibnote` `\thebibnote` `\bibnote` `\bibnotemark` `\bibnotetext` `\printbibnotes`

To avoid needing to load the notes2bib package, especially as the plan is to move that package to LaTeX3 internal syntax, achemso provides a minimal version here. The first step is to create macros which will need a guard against notes2bib already having been loaded. To allow the package and class to behave differently these are actually applied later.

```
\newcommand*\acs@niib@create{%
  \@namedef{ver@notes2bib.sty}{%
    2009/04/20 v1.6a Integrating notes into the bibliography (achemso
    version)
  }%
  \@ifundefined{c@bibnote}{\newcounter{bibnote}}{}
  \def\thebibnote{%
    Note-\the\value{bibnote}%
  }%
  \DeclareRobustCommand*{\bibnote}[1][\thebibnote]{%
    \stepcounter{bibnote}%
    \def\acs@niib@after@text{\cite{##1}}%
    \acs@niib@text{##1}%
  }%
  \DeclareRobustCommand*{\bibnotemark}[1][\thebibnote]{%
    \stepcounter{bibnote}%
    \cite{##1}%
```

```
  }%
  \DeclareRobustCommand*{\bibnotetext}[1][\thebibnote]{%
    \let\acs@niib@after@text\relax
    \acs@niib@text{##1}%
  }%
  \newcommand*\printbibnotes{%
    \ifnum\the\value{bibnote}>\z@\relax
      \nocite{achemso-control}%
      \acs@bibliography{acs-\jobname}%
    \fi
  }%
}
```

`\acs@niib@after@text` After the text.

```
\newcommand*\acs@niib@after@text{}
```

`\acs@niib@text` The `\acs@niib@text` macro is the outer part of the writing system. It does not absorb the text of note, as without ε-TeX this is bad news. The same file is used for notes and the control entry for the bibliography style.

```
\newcommand*\acs@niib@text{%
  \@bsphack
  \if@filesw
    \expandafter\acs@niib@write
  \else
    \expandafter\acs@niib@no@write
  \fi
}
```

`\acs@niib@write` `\acs@niib@write@aux@i` `\acs@niib@write@aux@ii` Writing verbatim without ε-TeX.

```
\newcommand*\acs@niib@write[1]{%
  \begingroup
    \let\do\@makeother
    \dospecials
    \catcode`\{ 1\relax
    \catcode`\} 2\relax
    \acs@niib@write@aux@i{#1}%
}
\newcommand*\acs@niib@write@aux@i[1]{%
  \long\def\@tempa##1{%
      \def\@tempa{##1}%
      \@onelevel@sanitize\@tempa
    \expandafter\endgroup
    \expandafter\def\expandafter\@tempa\expandafter{\@tempa}%
    \acs@niib@write@aux@ii{#1}%
  }%
  \catcode`\^^M 10\relax
  \@tempa
}
\newcommand*\acs@niib@write@aux@ii[1]{%
  \immediate\write\acs@bib@file{%
    @Misc\string{#1,^^J%
    \space\space note = \string{\@tempa\string},^^J%
    \string}^^J%
  }%
```

```
  \@esphack
  \acs@niib@after@text
}
```

\acs@niib@no@write If no files are to be written, a bit of tidying up.

```
\newcommand\acs@niib@no@write[2]{%
  \@esphack
  \acs@niib@after@text
}
```

\nmv@natbib@detect \acs@nmv@activate \acs@autonote The functionality of notes2bib is combined with the standard `\cite` macro, to give automatic note-like data in the bibliography.

```
\AtBeginDocument{
  \def\nmv@natbib@detect{%
    \ifNAT@super
      \expandafter\acs@nmv@activate
    \else
      \expandafter\acs@autonote
    \fi
  }
}
\newcommand*\acs@nmv@activate{%
  \let\nmv@citex@nat\@citex
  \let\@citex\nmv@citex
  \let\nmv@cite\cite
  \renewcommand*{\cite}[2][]{%
    \nmv@ifmtarg{##1}{%
      \nmv@citetrue
      \nmv@cite{##2}%
    }{%
      \nocite{##2}%
      \bibnote{Ref.~\citenum{##2}, ##1}%
    }%
  }%
}
\newcommand*\acs@autonote{%
  \let\nmv@cite\cite
  \renewcommand*{\cite}[2][]{%
    \nmv@ifmtarg{##1}{%
      \nmv@cite{##2}%
    }{%
      \nocite{##2}%
      \bibnote{Ref.~\citenum{##2}, ##1}%
    }%
  }%
}
```

\acs@bib@file \acs@bib@message \acs@bib@name Some information or creating the control file for BibTeX is set up.

```
\newwrite\acs@bib@file
\newcommand*\acs@bib@message{%
  This is an auxiliary file used by the ‘achemso’ bundle.^^J%
  This file may safely be deleted. It will be recreated as required.^^J
}
\newcommand*\acs@bib@name{acs-\jobname.bib}
```

\acs@bib@write
\acs@bib@write@aux

The control information for BibTeX needs to be written to a special file. The main writing macro is quite simple. Actually writing the information is left to the code for `\bibliography`, so that this only happens if needed.

```
\newcommand*\acs@bib@write{%
  \if@filesw
    \expandafter\acs@bib@write@aux
  \fi
}
\AtBeginDocument{\acs@bib@write}
\newcommand*\acs@bib@write@aux{%
  \immediate\openout\acs@bib@file\acs@bib@name\relax
  \immediate\write\acs@bib@file{\acs@bib@message}%
  \edef\@tempa##1##2{%
    \space\space##1\space = "##2",^^J%
  }%
  \immediate\write\acs@bib@file{%
    @Control\string{%
      achemso-control,^^J%
      \@tempa{ctrl-article-title\space}{%
        \ifacs@articletitle yes\else no\fi
      }%
      \@tempa{ctrl-chapter-title\space}{%
        \ifacs@chaptertitle yes\else no\fi
      }%
      \@tempa{ctrl-doi\space\space\space\space\space\space\space
        \space\space\space\space}{%
        \ifacs@doi yes\else no\fi
      }%
      \@tempa{ctrl-etal-number\space\space\space}{\acs@maxauthors}%
      \@tempa{ctrl-etal-firstonly}{%
        \ifacs@etal@truncate no\else yes\fi
      }%
    \string}^^J%
  }%
  \immediate\write\@auxout{%
    \string\citation\string{achemso-control\string}%
  }%
  \AtEndDocument{%
    \immediate\closeout\acs@bib@file\relax
  }%
}
```

\acs@bibliography
\bibliography

The `\bibliography` macro is now patched so that everything works correctly.

```
\AtBeginDocument{%
  \let\acs@bibliography\bibliography
  \def\bibliography#1{%
    \acs@bibliography{acs-\jobname,#1}%
  }%
}
```

\latin

The journal *ACS Nano* formats Latin phrases differently from every other ACS journal: we provide a `\latin` command to cover this.

```
\AtBeginDocument{
```

```
  \providecommand{\latin}[1]{#1}
}
⟨/package | class⟩
```

8.4 Late class-only code

Most of the power of the class is now created. First, a few options are reset so that any given by the user are effectively ignored.

```
⟨*class⟩
\setkeys{acs}{
  abbreviations = false,
  articletitle  = true,
  biblabel      = brackets,
  biochem       = false,
  doi           = false,
  etalmode      = firstonly,
  keywords      = false,
  maxauthors    = 15,
  super         = true
}
```

When using the class, notes2bib is always emulated. Other standard support packages can now be loaded.

```
\acs@niib@create
\RequirePackage[margin=2.54cm]{geometry}
\RequirePackage{
  caption,
  float,
  graphicx,
  setspace,
  url
}
\ifacs@hyperref
  \expandafter\RequirePackage
\else
  \expandafter\@gobble
\fi
  {hyperref}
\AtBeginDocument{\doublespacing}
```

`\title` `\@title` `\acs@title@short`

For the meta-data, the REVTEX bundle provides a good model for the commands to give the author. `\gdef` is used here to avoid any odd grouping issues.

```
\renewcommand*{\title}[2][]{%
  \gdef\acs@title@short{#1}%
  \gdef\@title{#2}%
  \ifx\acs@title@short\@empty
    \global\let\acs@title@short\@title
  \fi
}
\@onlypreamble\title
```

`\acs@author@cnt` `\acs@affil@cnt` `\acs@affil@alt@cnt`

achemso tracks the number authors, affiliations and alternative affiliations.

```
\newcount\acs@author@cnt
```

```
\newcount\acs@affil@cnt
\newcount\acs@affil@alt@cnt
```

\acs@footnote@cnt
\acs@affil@marker@cnt

Two counts for getting affiliation footnotes correct.

```
\newcount\acs@footnote@cnt
\newcount\acs@affil@marker@cnt
```

\author

The author macro stores the current author details and sets the affiliation of the author to the current one. Everything is `\global` so that there is no possibility of begin trapped inside a group. The affiliation counter is always one behind, and so it is locally incremented to keep the logic of the code clear elsewhere.

```
\def\author#1{%
  \global\advance\acs@author@cnt\@ne\relax
  \expandafter\gdef\csname @author@\@roman\acs@author@cnt\endcsname{#1}%
  \begingroup
    \advance\acs@affil@cnt\@ne
    \expandafter\xdef\csname @author@affil@\@roman
      \acs@author@cnt\endcsname
      {\the\acs@affil@cnt}%
  \endgroup
}
\@onlypreamble\author
```

\and
\thanks

Neither `\and` nor `\thanks` are used by the document class.

```
\def\and{%
  \acs@warning{%
    \string\and\space not used by the achemso class: please see
    the\MessageBreak package documentation for details%
  }%
}
\def\thanks{%
  \acs@warning{%
    \string\thanks\space not used by the achemso class: please see
    the\MessageBreak the package documentation for details%
  }%
}
```

\affiliation

As with `\author`, everything is `\global` just in case. The system insists that affiliations come after authors. Before anything is committed, a check is made that the affiliation has not already been seen.

```
\newcommand*\affiliation[2][\relax]{%
  \ifnum\acs@author@cnt>\z@\relax
    \acs@affil@ifdup{#2}{%
      \acs@affil@swap{#2}%
    }{%
      \global\advance\acs@affil@cnt\@ne\relax
      \expandafter\gdef\csname @address@\@roman\acs@affil@cnt\endcsname
        {#2}%
      \ifx\relax#1\relax
        \expandafter\gdef\csname @affil@\@roman\acs@affil@cnt\endcsname
          {#2}%
      \else
        \expandafter\gdef\csname @affil@\@roman\acs@affil@cnt\endcsname
```

```
        {#1}%
      \fi
    }%
  \else
    \acs@warning{Affiliation with no author}%
  \fi
}
\@onlypreamble\affiliation
```

\acs@affil@ifdup
\acs@affil@ifdup@aux

A short test for two addresses being identical.

```
\newcommand*\acs@affil@ifdup[1]{%
  \begingroup
    \def\@tempa{#1}%
    \@tempswafalse
    \@tempcnta\z@\relax
    \acs@affil@ifdup@aux
    \if@tempswa
      \aftergroup\@firstoftwo
    \else
      \aftergroup\@secondoftwo
    \fi
  \endgroup
}
\newcommand*\acs@affil@ifdup@aux{%
  \advance\@tempcnta\@ne\relax
  \expandafter\expandafter\expandafter\def\expandafter\expandafter
    \expandafter\@tempb\expandafter\expandafter\expandafter
    {\csname @address@\@roman\@tempcnta\endcsname}%
  \ifx\@tempa\@tempb
    \expandafter\@tempswatrue
  \else
    \ifnum\@tempcnta<\acs@affil@cnt\relax
      \expandafter\expandafter\expandafter\acs@affil@ifdup@aux
    \fi
  \fi
}
```

\acs@affil@swap
\acs@affil@swap@aux

If the affiliation has already been given, then all of the authors need to be checked to make sure that the correct affiliation is used. First, the loop from above is used to find the correct number for the duplicate.

```
\newcommand*\acs@affil@swap[1]{%
  \begingroup
    \def\@tempa{#1}%
    \@tempcnta\z@\relax
    \@tempcntb\z@\relax
    \acs@affil@ifdup@aux
    \advance\acs@affil@cnt\@ne\relax
    \acs@affil@swap@aux
  \endgroup
}
\newcommand*\acs@affil@swap@aux{%
  \advance\@tempcntb\@ne\relax
  \expandafter\ifnum\csname @author@affil@\@roman\@tempcntb\endcsname
    = \acs@affil@cnt\relax
```

```
    \expandafter\xdef\csname @author@affil@\@roman\@tempcntb\endcsname{%
      \the\@tempcnta
    }%
  \fi
  \ifnum\@tempcntb<\acs@author@cnt\relax
    \expandafter\acs@affil@swap@aux
  \fi
}
```

`\alsoaffiliation` `\acs@alsoaffil@find` To allow complex affiliations , two commands are needed. The first deals with affiliations that are in some way shared by several authors. This is tracked on a per author basis.

```
\newcommand*\alsoaffiliation[2][\relax]{%
  \ifnum\acs@author@cnt>\z@\relax
    \acs@affil@ifdup{#2}{%
      \acs@alsoaffil@find{#2}%
    }{%
      \global\advance\acs@affil@cnt\@ne\relax
      \@tempcnta\acs@affil@cnt\relax
      \expandafter\gdef\csname @address@\@roman\acs@affil@cnt\endcsname
        {#2}%
      \ifx\relax#1\relax
        \expandafter\gdef\csname @affil@\@roman\acs@affil@cnt\endcsname
          {#2}%
      \else
        \expandafter\gdef\csname @affil@\@roman\acs@affil@cnt\endcsname
          {#1}%
      \fi
    }%
    \@ifundefined{@author@alsoaffil@\@roman\acs@author@cnt}{%
      \expandafter\xdef\csname @author@alsoaffil@\@roman\acs@author@cnt
        \endcsname{\the\@tempcnta}%
    }{%
      \expandafter\xdef\csname @author@alsoaffil@\@roman\acs@author@cnt
        \endcsname{%
        \csname @author@alsoaffil@\@roman\acs@author@cnt\endcsname
        ,\the\@tempcnta
      }%
    }%
  \else
    \acs@warning{Affiliation with no author}%
  \fi
}
\newcommand*\acs@alsoaffil@find[1]{%
  \begingroup
    \def\@tempa{#1}%
    \@tempcnta\z@\relax
    \@tempcntb\z@\relax
    \acs@affil@ifdup@aux
  \expandafter\endgroup
  \expandafter\@tempcnta\the\@tempcnta\relax
}
```

`\altaffiliation` For the alternative affiliation, a second count is kept, and the affiliation is "at-

tached" to the author. The way these are stored means that the appropriate affiliation number can be recovered later, and so printed correctly when things get complex.

```
\newcommand*\altaffiliation[1]{%
  \ifnum\acs@author@cnt>\z@\relax
    \begingroup
      \acs@altaffil@ifdup{#1}{%
        \expandafter\xdef\csname @author@altaffil@\@roman\acs@author@cnt
          \endcsname{\the\@tempcnta}%
      }{%
        \global\advance\acs@affil@alt@cnt\@ne\relax
        \expandafter\gdef\csname @altaffil@\@roman\acs@affil@alt@cnt
          \endcsname{#1}%
        \expandafter\xdef\csname @author@altaffil@\@roman\acs@author@cnt
          \endcsname{\the\acs@affil@alt@cnt}%
      }%
    \endgroup
  \else
    \acs@warning{Affiliation with no author}%
  \fi
}
\@onlypreamble\altaffiliation
```

`\acs@altaffil@ifdup` `\acs@altaffil@ifdup@aux` This is very similar to the same routine for normal affiliations but with the appropriate name changes.

```
\newcommand*\acs@altaffil@ifdup[1]{%
  \def\@tempa{#1}%
  \@tempswafalse
  \@tempcnta\z@\relax
  \ifnum\acs@affil@alt@cnt>\z@\relax
    \expandafter\acs@altaffil@ifdup@aux
  \fi
  \if@tempswa
    \expandafter\@firstoftwo
  \else
    \expandafter\@secondoftwo
  \fi
}
\newcommand*\acs@altaffil@ifdup@aux{%
  \advance\@tempcnta\@ne\relax
  \expandafter\expandafter\expandafter\def\expandafter\expandafter
    \expandafter\@tempb\expandafter\expandafter\expandafter
    {\csname @altaffil@\@roman\@tempcnta\endcsname}%
  \ifx\@tempa\@tempb
    \expandafter\@tempswatrue
  \else
    \ifnum\@tempcnta<\acs@affil@alt@cnt\relax
      \expandafter\expandafter\expandafter\acs@altaffil@ifdup@aux
    \fi
  \fi
}
```

`\email` E-mail addresses are attached to authors as well.

```
\newcommand*\email[1]{%
  \ifnum\acs@author@cnt>\z@\relax
    \expandafter\gdef\csname @email@\@roman\acs@author@cnt\endcsname
      {#1}%
  \else
    \acs@warning{E-mail with no author}%
  \fi
}
\@onlypreamble\email
```

\fax \phone Fax and phone numbers are similar.

```
\newcommand*\fax[1]{%
  \ifnum\acs@author@cnt>\z@\relax
    \expandafter\gdef\csname @fax@\@roman\acs@author@cnt\endcsname
      {#1}%
  \else
    \acs@warning{Fax number with no author}%
  \fi
}
\@onlypreamble\fax
\newcommand*\phone[1]{%
  \ifnum\acs@author@cnt>\z@\relax
    \expandafter\gdef\csname @phone@\@roman\acs@author@cnt\endcsname
      {#1}%
  \else
    \acs@warning{Phone number with no author}%
  \fi
}
\@onlypreamble\phone
```

\abbreviations \@abbreviations \keywords \@keywords Some journals use these.

```
\newcommand*\abbreviations[1]{%
  \gdef\@abbreviations{#1}%
}
\newcommand*\@abbreviations{}
\@onlypreamble\abbreviations
\newcommand*\keywords[1]{%
  \gdef\@keywords{#1}%
}
\newcommand*\@keywords{}
\@onlypreamble\keywords
```

\acs@abbreviations@print \acs@keywords@print \acs@title@short@print For printing the key simple meta-data.

```
\newcommand*\acs@abbreviations@print{%
  \ifx\@abbreviations\@empty\else
    \section*{Abbreviations}
    \@abbreviations
    \par
  \fi
}
\newcommand*\acs@keywords@print{%
  \ifx\@keywords\@empty\else
    \section*{Keywords}
    \@keywords
```

```
    \par
  \fi
}
\newcommand*\acs@title@short@print{%
  \section*{Running header}
  \acs@title@short
  \par
}
```

\acs@space@pre@title
\acs@space@post@title
\acs@space@post@author
\acs@space@post@address
\acs@space@post@email
\acs@maketitle@width

Lengths for \@maketitle.

```
\newlength\acs@space@pre@title
\setlength\acs@space@pre@title{2em}
\newlength\acs@space@post@title
\setlength\acs@space@post@title{1.5em}
\newlength\acs@space@post@author
\setlength\acs@space@post@author{1em}
\newlength\acs@space@post@address
\setlength\acs@space@post@address{1em}
\newlength\acs@space@post@email
\setlength\acs@space@post@email{1.5em}
\newlength\acs@maketitle@width
\setlength\acs@maketitle@width{\textwidth}
```

\affilsize
\authorsize
\emailsize
\titlesize

Some simple size commands.

```
\newcommand*\affilsize{\normalsize}
\newcommand*\authorsize{\large}
\newcommand*\emailsize{\normalsize}
\newcommand*\titlesize{\LARGE}
```

\authorfont
\authorfont
\emailfont
\titlefont

Font settings for \@maketitle.

```
\newcommand*\affilfont{\itshape}
\newcommand*\authorfont{\sffamily}
\newcommand*\emailfont{}
\newcommand*\titlefont{\bfseries\sffamily}
```

\ps@acs

A shortcut to make page styles.

```
\newcommand*{\ps@acs}{}
\let\ps@acs\ps@plain
```

\@maketitle
\@maketitle@title@hook

With the changes outlined above in place, a new \@maketitle macro is needed. This is partially a copy of the existing, but rather heavily modified.

```
\def\@maketitle{%
  \pagestyle{acs}%
  \ifnum\acs@author@cnt<\z@\relax
    \acs@warning{No authors defined: At least one author is required}%
  \fi
  \newpage
  \null
  \vspace*{\acs@space@pre@title}%
  \begin{center}
    \begin{minipage}{\acs@maketitle@width}
      \begin{center}
        {%
```

```
          \titlefont
          \titlesize
          \let\@fnsymbol\acs@author@fnsymbol
          \let\footnote\acs@title@footnote
          \acs@maketitle@suppinfo \@title
          \acs@title@footnote@check
          \global\acs@footnote@cnt\c@footnote
          \@maketitle@title@hook
          \par
        }%
        \vspace*{\acs@space@post@title}%
        {%
          \authorsize
          \authorfont
          \frenchspacing
          \acs@author@list
          \par
        }%
        \vspace*{\acs@space@post@author}%
        {%
          \affilsize
          \affilfont
          \acs@address@list
          \par
        }%
        \vspace*{\acs@space@post@address}%
        {%
          \emailsize
          \emailfont
          \ifacs@email
            \expandafter\acs@contact@details
          \fi
        }%
        \vspace*{\acs@space@post@email}%
      \end{center}
    \end{minipage}
  \end{center}%
}
\newcommand*\@maketitle@title@hook{}
```

`\acs@maketitle@suppinfo` This is spun out so that it can be avoided if necessary: this is done on the sly.

```
\newcommand*\acs@maketitle@suppinfo{%
  \ifx\acs@manuscript\acs@manuscript@suppinfo
    Supporting Information:\\
  \fi
}
```

`\acs@title@footnote` `\acs@title@footnote@check` Footnotes need to be created so that they appear correctly.

```
\newcommand*\acs@title@footnote[1]{%
  \footnotemark
  \g@addto@macro\@thanks{\footnotetext{#1}}%
}
\newcommand\acs@title@footnote@check{%
  \ifx\@thanks\@empty
```

```
  \else
    \begingroup
      \toks@=\expandafter{\@thanks}%
      \xdef\@thanks{%
        \begingroup
          \let\noexpand\@fnsymbol\noexpand\acs@author@fnsymbol
          \the\toks@
        \endgroup
      }%
    \endgroup
  \fi
}
```

\acs@contact@details A general contact details macro.

```
\newcommand*\acs@contact@details{%
  { \sffamily E-mail: \acs@email@list }%
  \acs@number@list
}
```

\@thanks The \@thanks macro is used as a hook to generate the footnotes if needed.

```
\let\@thanks\@empty
```

\acs@author@list Printing the author list needs to do several things. The appropriate separators between authors are created and the author names themselves are printed.

```
\newcommand*\acs@author@list{%
  \@tempcnta\z@\relax
  \ifnum\acs@author@cnt=\z@\relax\else
    \expandafter\acs@author@list@main
  \fi
}
```

\acs@author@footnotes
\acs@author@list@main The main control macro for producing the author list iterates over each author on the list. The result is stored as \acs@author@listing.

```
\newcommand*\acs@author@footnotes{}
\newcommand*\acs@author@list@main{%
  \advance\@tempcnta\@ne\relax
  \def\acs@author@footnotes{}%
  \acs@author@list@and
  \space
  \@nameuse{@author@\@roman\@tempcnta}%
  \acs@author@list@comma
  \acs@author@star
  \acs@author@affil
  \acs@author@affil@also
  \acs@author@affil@alt
  \ifx\@empty\acs@author@footnotes\else
    \textsuperscript{\acs@author@footnotes}%
  \fi
  \ifnum\@tempcnta<\acs@author@cnt\relax
    \expandafter\acs@author@list@main
  \fi
}
```

\acs@author@list@and
\acs@author@list@comma

Simple checks to add an "and" and a comma.

```
\newcommand*\acs@author@list@and{%
  \ifnum\acs@author@cnt=\@ne\relax\else
    \ifnum\@tempcnta=\acs@author@cnt\relax
      \space and%
    \fi
  \fi
}
\newcommand*\acs@author@list@comma{%
  \ifnum\acs@author@cnt>\tw@\relax
    \ifnum\@tempcnta<\acs@author@cnt\relax
      ,%
    \fi
  \fi
}
```

\acs@author@star
\acs@author@star@aux

A check for an e-mail for an author: if so, add a star.

```
\newcommand*\acs@author@star{%
  \acs@ifundefined{@email@\@roman\@tempcnta}{}{%
    \acs@author@star@aux
  }%
}
\newcommand*\acs@author@star@aux{%
  \protected@edef\acs@author@footnotes{%
    \acs@author@fnsymbol{\z@}%
    \ifnum\acs@affil@cnt>\@ne\relax
      ,%
    \else
      \ifnum\acs@affil@alt@cnt>\z@\relax
        ,%
      \fi
    \fi
  }%
}
```

\acs@author@affil
\acs@author@affil@aux

The main affiliation of the author is checked for, and assuming one is found the appropriate symbol is added to the list.

```
\newcommand*\acs@author@affil{%
  \acs@ifundefined{%
    @affil@\@roman\csname @author@affil@\@roman\@tempcnta\endcsname
  }{%
    \acs@warning{%
      No affiliation given for author\MessageBreak
      \@nameuse{@author@\@roman\@tempcnta}%
    }%
  }{%
    \acs@author@affil@aux
  }%
}
\newcommand*\acs@author@affil@aux{%
  \ifnum\acs@affil@cnt>\@ne\relax
    \expandafter\acs@affil@marker@cnt\csname @author@affil@\@roman
      \@tempcnta\endcsname\relax
    \advance\acs@affil@marker@cnt\acs@footnote@cnt\relax
```

```
    \protected@edef\acs@author@footnotes{%
      \acs@author@footnotes
      \acs@author@fnsymbol{\acs@affil@marker@cnt}%
    }%
  \else
    \ifnum\acs@affil@alt@cnt>\z@\relax
      \acs@affil@marker@cnt\@ne\relax
      \advance\acs@affil@marker@cnt\acs@footnote@cnt\relax
      \protected@edef\acs@author@footnotes{%
        \acs@author@footnotes
        \acs@author@fnsymbol{\acs@affil@marker@cnt}%
      }%
    \fi
  \fi
}
```

\acs@author@affil@also
\acs@author@affil@also@aux

The "also" affiliations are generated by a loop as there may be more than one.

```
\newcommand*\acs@author@affil@also{%
  \acs@ifundefined{@author@alsoaffil@\@roman\@tempcnta}{}{%
    \acs@author@affil@also@aux
  }%
}
\newcommand*\acs@author@affil@also@aux{%
  \expandafter\@for\expandafter\@tempa\expandafter:\expandafter
    =\csname @author@alsoaffil@\@roman\@tempcnta\endcsname\do{%
    \acs@affil@marker@cnt\@tempa\relax
    \advance\acs@affil@marker@cnt\acs@footnote@cnt\relax
    \protected@edef\acs@author@footnotes{%
      \acs@author@footnotes
      ,%
      \acs@author@fnsymbol{\acs@affil@marker@cnt}%
    }%
  }%
}
```

\acs@author@affil@alt
\acs@author@affil@alt@aux

Alternative affiliations get the correct affiliation number back out from the stored data. There are then two corrections: one for the total number of main affiliations and a second in case there is a footnote to the title.

```
\newcommand*\acs@author@affil@alt{%
  \acs@ifundefined{@author@altaffil@\@roman\@tempcnta}{}{%
    \acs@author@affil@alt@aux
  }%
}
\newcommand*\acs@author@affil@alt@aux{%
  \expandafter\acs@affil@marker@cnt
    \csname @author@altaffil@\@roman\@tempcnta\endcsname\relax
  \advance\acs@affil@marker@cnt\acs@affil@cnt\relax
  \advance\acs@affil@marker@cnt\acs@footnote@cnt\relax
  \protected@edef\acs@author@footnotes{%
    \acs@author@footnotes
    ,%
    \acs@author@fnsymbol{\acs@affil@marker@cnt}%
  }%
}
```

\acs@author@fnsymbol
\acs@author@fnsymbol@aux
\acs@author@fnsymbol@loop
acs@author@fnsymbol@loop@aux@i
cs@author@fnsymbol@loop@aux@ii
acs@author@fnsymbol@loop@aux@m
acs@author@fnsymbol@loop@aux@Q
\acs@author@fnsymbol@symbol

The ACS have an extended list of symbols. The star appears at the special position zero.

```
\newcommand*{\acs@author@fnsymbol}[1]{%
  \ensuremath{%
    \expandafter\acs@author@fnsymbol@aux\expandafter{\number#1 }%
  }%
}
\newcommand*{\acs@author@fnsymbol@aux}[1]{%
  \ifnum#1>10 %
    \expandafter\acs@author@fnsymbol@loop
  \else
    \expandafter\acs@author@fnsymbol@symbol
  \fi
    {#1}%
}
\newcommand*{\acs@author@fnsymbol@loop}[1]{%
   \acs@author@fnsymbol@loop@aux@i#1%
}
\newcommand*{\acs@author@fnsymbol@loop@aux@i}[2]{%
  \acs@author@fnsymbol@symbol{\ifnum#2=0 10\else #2\fi}%
  \expandafter\acs@author@fnsymbol@loop@aux@ii\romannumeral #1000Q{}%
    {\acs@author@fnsymbol@symbol{\ifnum#2=0 10\else #2\fi}}%
}
\newcommand*{\acs@author@fnsymbol@loop@aux@ii}[1]{%
  \@nameuse{acs@author@fnsymbol@loop@aux@#1}%
}
\def\acs@author@fnsymbol@loop@aux@m#1Q#2#3{%
  \acs@author@fnsymbol@loop@aux@ii#1Q{#2#3}{#3}%
}
\newcommand*{\acs@author@fnsymbol@loop@aux@Q}[2]{#1}
\newcommand*{\acs@author@fnsymbol@symbol}[1]{%
  \ifcase #1 *\or
    \dagger\or
      \ddagger\or
        \P\or
          \S\or
            \|\or
              \bot\or
                \#\or
                  @\or
                    \triangle\or
                      \nabla
    \fi
}
```

\acs@address@list
\acs@address@list@auxi
\acs@address@list@auxii

Loop over the addresses and any extra affiliations and print them all: if there is only one, omit the marker entirely. There is also a need to watch out for any footnotes from the title.

```
\newcommand*\acs@address@list{%
  \ifnum\acs@affil@cnt>\z@
    \expandafter\acs@address@list@auxi
  \else
    \acs@warning{No affiliations: at least one affiliation is needed}%
  \fi
```

```
}
\newcommand*\acs@address@list@auxi{%
  \ifnum0%
    \ifnum\acs@affil@cnt>\@ne    1\fi
    \ifnum\acs@affil@alt@cnt>\z@ 1\fi
      >\z@
    \expandafter\acs@address@list@auxii
  \else
    \@address@i\par
  \fi
}
\newcommand*\acs@address@list@auxii{%
  \@tempcnta\z@
  \acs@affil@marker@cnt\acs@footnote@cnt
  \loop\ifnum\@tempcnta<\acs@affil@cnt
    \advance\@tempcnta\@ne
    \advance\acs@affil@marker@cnt\@ne
    \acs@author@fnsymbol{\acs@affil@marker@cnt}%
    \@nameuse{@address@\@roman\@tempcnta}\par
  \repeat
  \@tempcnta\z@
  \loop\ifnum\@tempcnta<\acs@affil@cnt
    \advance\@tempcnta\@ne
    \advance\acs@affil@marker@cnt\@ne
    \acs@ifundefined{@altaffil@\@roman\@tempcnta}
      {}
      {%
        \acs@author@fnsymbol{\acs@affil@marker@cnt}%
        \@nameuse{@altaffil@\@roman\@tempcnta}\par
      }%
  \repeat
}
```

\acs@fnsymbol@org
\acs@affil@list
\acs@affil@list@aux

Footnotes are done in two stages. First the main affiliation is handled, then the possible alternative. There is a need to check for the possibility that there is only one main affiliation but one or more alternative ones.

```
\newcommand*\acs@fnsymbol@org{}
\newcommand*\acs@affil@list{%
  \let\acs@fnsymbol@org\@fnsymbol
  \let\@fnsymbol\acs@author@fnsymbol
  \@tempcnta\z@\relax
  \@tempcntb\z@\relax
  \ifnum\acs@affil@cnt>\@ne\relax
    \expandafter\acs@affil@list@aux
  \else
    \ifnum\acs@affil@alt@cnt>\z@\relax
      \acs@affil@marker@cnt\@ne\relax
      \advance\acs@affil@marker@cnt\acs@footnote@cnt\relax
      \footnotetext[\acs@affil@marker@cnt]{\@affil@i}%
      \@tempcnta\@ne\relax
    \fi
  \fi
  \ifnum\acs@affil@alt@cnt>\z@\relax
    \expandafter\acs@affil@alt@list
```

```
  \fi
  \let\@fnsymbol\acs@fnsymbol@org
}
\newcommand*\acs@affil@list@aux{%
  \advance\@tempcnta\@ne\relax
  \acs@affil@marker@cnt\@tempcnta\relax
  \advance\acs@affil@marker@cnt\acs@footnote@cnt\relax
  \footnotetext[\acs@affil@marker@cnt]{%
    \@nameuse{@affil@\@roman\@tempcnta}%
  }%
  \ifnum\@tempcnta<\acs@affil@cnt\relax
    \expandafter\acs@affil@list@aux
  \fi
}
```

\acs@affil@alt@list
\acs@affil@alt@lista@aux

The secondary loop for alternative affiliations is similar.

```
\newcommand*\acs@affil@alt@list{%
  \advance\@tempcntb\@ne\relax
  \acs@ifundefined{@altaffil@\@roman\@tempcntb}{}{%
    \acs@altaffil@foot@aux
  }
  \ifnum\@tempcntb<\acs@author@cnt\relax
    \expandafter\acs@affil@alt@list
  \fi
}
\newcommand*\acs@altaffil@foot@aux{%
  \advance\@tempcnta\@ne\relax
  \acs@affil@marker@cnt\@tempcnta\relax
  \advance\acs@affil@marker@cnt\acs@footnote@cnt\relax
  \footnotetext[\acs@affil@marker@cnt]{%
    \@nameuse{@altaffil@\@roman\@tempcntb}%
  }%
}
```

\acs@email@list@font
\acs@email@list
\acs@email@list@aux

The final piece of meta-data to print is the e-mail address list. The total number of e-mail addresses given it counted in \@tempcntb, which means a warning can be given if there are none. The group is used so that \UrlFont can be set correctly.

```
\newcommand*\acs@email@list@font{\sf}
\newcommand*\acs@email@list{%
  \begingroup
    \def\UrlFont{\acs@email@list@font}%
    \@tempcnta\z@\relax
    \@tempcntb\z@\relax
    \acs@email@list@aux
    \ifnum\@tempcntb=\z@\relax
      \acs@warning{%
        No e-mail given:\MessageBreak
        at lest one author must have a contact e-mail%
      }%
    \fi
  \endgroup
}
\newcommand*\acs@email@list@aux{%
```

```
  \advance\@tempcnta\@ne\relax
  \ifnum\@tempcnta>\acs@author@cnt\relax\else
    \acs@ifundefined{@email@\@roman\@tempcnta}{}{%
      \advance\@tempcntb\@ne\relax
      \ifnum\@tempcntb>\@ne\relax
        ;
      \fi
      \expandafter\expandafter\expandafter\url\expandafter
        \expandafter\expandafter{%
           \csname @email@\@roman\@tempcnta\endcsname
      }%
    }%
    \expandafter\acs@email@list@aux
  \fi
}
```

\acs@number@list
\acs@number@list@aux@i
\acs@number@list@aux@ii

Listing phone and fax numbers is easier as they don't have to be given. Everything is done in one block so that it is possible to know whether to add a new line and also to keep everything together.

```
\newcommand*\acs@number@list{%
  \begingroup
    \acs@number@list@aux@i{phone}%
    \let\@tempb\@tempa
    \acs@number@list@aux@i{fax}%
    \ifx\@tempa\@empty
      \let\@tempa\@tempb
    \else
      \ifx\@tempb\@empty\else
        \protected@edef\@tempa{%
          \@tempb.\space\@tempa
        }%
      \fi
    \fi
    \ifx\@tempa\@empty\else
      \par
      \@tempa
    \fi
  \endgroup
}
\newcommand*\acs@number@list@aux@i[1]{%
  \def\@tempa{}%
  \@tempcnta\z@\relax
  \def\acs@number@list@aux@ii{%
    \advance\@tempcnta\@ne\relax
    \ifnum\@tempcnta>\acs@author@cnt\relax\else
      \acs@ifundefined{@#1@\@roman\@tempcnta}{}{%
        \acs@ifundefined{@email@\@roman\@tempcnta}{}{%
          \ifx\@tempa\@empty
            \edef\@tempa{%
              \@nameuse{@#1@\@roman\@tempcnta}%
            }%
          \else
            \edef\@tempa{%
              \@tempa
```

```
              ;
              \@nameuse{@#1@\@roman\@tempcnta}%
            }%
          \fi
        }%
      }%
      \expandafter\acs@number@list@aux@ii
    \fi
  }%
  \acs@number@list@aux@ii
  \ifx\@tempa\@empty\else
    \protected@edef\@tempa{%
      \MakeUppercase#1: \@tempa
    }%
  \fi
}
\newcommand*\acs@number@list@aux@ii{}
```

`\endabstract`
`\acs@abstract@extras`

```
\g@addto@macro\endabstract{%
  \aftergroup\acs@abstract@extras
}
\newcommand*{\acs@abstract@extras}{%
  \ifacs@abbreviations
    \acs@abbreviations@print
    \par
  \fi
  \ifacs@keywords
    \acs@keywords@print
    \par
  \fi
}
```

`\acs@maketitle@extras`
`\acs@maketitle@extras@hook`

A couple of things might need to be added to `\maketitle`.

```
\newcommand*\acs@maketitle@extras{%
  \acs@maketitle@extras@hook
}
\newcommand*\acs@maketitle@extras@hook{}
\g@addto@macro{\maketitle}{\acs@maketitle@extras}
```

`\maketitle` is required by the document class, and must start the document. No variation is allowed, and so it is done automatically.

```
\g@addto@macro{\document}{\maketitle}
```

`scheme` (*env.*)
`chart` (*env.*)
`graph` (*env.*)

Three new float types are provided, `scheme`, `chart` and `graph`. These are the most obvious types; for graphs, a slight problem arises with the file extension.

```
\newfloat{scheme}{htbp}{los}
\floatname{scheme}{Scheme}
\newfloat{chart}{htbp}{loc}
\floatname{chart}{Chart}
\newfloat{graph}{htbp}{loh}
\floatname{graph}{Graph}
```

\schemename \chartname \graphname

Naming is set up in the same way as the kernel floats.

```
\newcommand*\schemename{Scheme}
\newcommand*\chartname{Chart}
\newcommand*\graphname{Graph}
```

The standard floats should appear "here" by default.

```
\floatplacement{table}{htbp}
\floatplacement{figure}{htbp}
\floatstyle{plaintop}
\restylefloat{table}
```

\acs@floatboxreset

Floats are all centred.

```
\let\acs@floatboxreset\@floatboxreset
\def\@floatboxreset{%
  \centering
  \acs@floatboxreset
}
```

\plainref \ref

For legacy support.

```
\newcommand*\plainref{}
\AtBeginDocument{\let\plainref\ref}
```

\acs@section \acs@subsection \acs@subsubsection \acs@startsection@orig

Both the numbering and existence of section headers may need to be altered. Some generic functions are therefore provided to deal with this cleanly. First, some original definitions are saved.

```
\newcommand*\acs@section{}
\let\acs@section\section
\newcommand*\acs@subsection{}
\let\acs@subsection\subsection
\newcommand*\acs@subsubsection{}
\let\acs@subsubsection\subsubsection
\newcommand*\acs@startsection@orig{}
\let\acs@startsection@orig\@startsection
```

\acs@startsection

A version of `\@startsection` which adds unnumbered sections to the TOC: modelled on amsart. This is active as standard.

```
\newcommand\acs@startsection[6]{%
 \if@noskipsec \leavevmode \fi
 \par \@tempskipa #4\relax
 \@afterindenttrue
 \ifdim \@tempskipa <\z@ \@tempskipa -\@tempskipa \@afterindentfalse\fi
 \if@nobreak \everypar{}\else
     \addpenalty\@secpenalty\addvspace\@tempskipa\fi
 \@ifstar{\@dblarg{\@sect{#1}{\@m}{#3}{#4}{#5}{#6}}}%
         {\@dblarg{\@sect{#1}{#2}{#3}{#4}{#5}{#6}}}%
}
\let\@startsection\acs@startsection
```

\acs@startsection@alt

An alternative version of `\@startsection` which never adds numbers.

```
\newcommand*\acs@startsection@alt[6]{%
  \if@noskipsec \leavevmode \fi
  \par \@tempskipa #4\relax
  \@afterindenttrue
```

```
 \ifdim \@tempskipa <\z@ \@tempskipa -\@tempskipa \@afterindentfalse\fi
 \if@nobreak \everypar{}\else
     \addpenalty\@secpenalty\addvspace\@tempskipa\fi
  \@ifstar{\@ssect{#3}{#4}{#5}{#6}}
          {\@ssect{#3}{#4}{#5}{#6}}%
}
```

`\acs@sections@none` `\acs@sections@none@aux`

When removing sections entirely, a gobble macro is needed.

```
\newcommand*\acs@sections@none{%
  \@ifstar{%
    \acs@sections@none@aux
  }{%
    \acs@sections@none@aux
  }%
}
\newcommand*\acs@sections@none@aux[2][]{%
  \acs@warning{%
    (Sub)section '#2' ignored%
  }%
}
```

`\SectionNumbersOff` `\SectionNumbersOn`

To macros to add or remove the section numbers. The standard setting for the class has them on, but some configurations will turn them off. The names of these functions are both in design space so that users can change the decision easily.

```
\newcommand*\SectionNumbersOff{%
  \let\@startsection\acs@startsection@alt
}
\@onlypreamble\SectionNumbersOff
\newcommand*\SectionNumbersOn{%
  \let\@startsection\acs@startsection
}
\@onlypreamble\SectionNumbersOn
```

`\SectionsOff` `\SectionsOn`

Quite similar for entire sections.

```
\newcommand*\SectionsOff{%
  \let\section\acs@sections@none
  \let\subsection\acs@sections@none
  \let\subsubsection\acs@sections@none
}
\@onlypreamble\SectionsOff
\newcommand*\SectionsOn{%
  \let\section\acs@section
  \let\subsection\acs@subsection
  \let\subsubsection\acs@subsubsection
}
\@onlypreamble\SectionsOn
```

`\tableofcontents`

Never print TOC in itself.

```
\begingroup
  \toks@=\expandafter{\tableofcontents}
  \xdef\tableofcontents{%
    \begingroup
```

```
      \let\noexpand\@startsection\noexpand\acs@startsection@orig
      \the\toks@
    \endgroup
  }
\endgroup
```

acknowledgement (*env.*) suppinfo (*env.*)

Simple named sections.

```
\newenvironment{acknowledgement}{%
  \acs@section*{\acknowledgementname}%
}{}
\newenvironment{suppinfo}{%
  \acs@section*{\suppinfoname}%
}{}
```

\acknowledgementname \bibsection \suppinfoname

A few macros need to get around the changes.

```
\newcommand*\acknowledgementname{Acknowledgement}
\AtEndOfClass{%
  \def\bibsection{%
    \acs@section*{\refname}%
  }%
}
\newcommand*\suppinfoname{Supporting Information Available}
```

\acs@abstract \acs@endabstract \acs@abstract@start \acs@abstract@end \acs@abstract@iffalse

Removing the abstract, if necessary, is done using a trick from the comment package. However, it code is copied here to keep requirements down.

```
\newcommand*\acs@abstract{}
\let\acs@abstract\abstract
\newcommand*\acs@endabstract{}
\let\acs@endabstract\endabstract
\begingroup
  \catcode`{ \active
  \catcode`} 12\relax
  \catcode`( 1\relax
  \catcode`) 2\relax
  \gdef\acs@abstract@start(%
    \acs@warning(%
      Abstract not allowed for this\MessageBreak
       manuscript type
    )%
    \@bsphack
    \catcode`{ \active
    \catcode`} 12\relax
    \let\end\fi
    \let{\acs@abstract@end% }
    \iffalse
  )%{
  \gdef\acs@abstract@end#1}(%
    \def\@tempa(#1)%
    \ifx\@tempa\@currenvir
      \@Esphack\endgroup
      \if@ignore
        \global\@ignorefalse
        \ignorespaces
      \fi
```

```
    \else
      \expandafter\acs@abstract@iffalse
    \fi
  )
\endgroup
\newcommand*\acs@iffalse{\iffalse}
```

\AbstractOff \AbstractOn

A very similar pattern to before.

```
\newcommand*\AbstractOff{%
  \let\abstract\acs@abstract@start
  \let\endabstract\acs@abstract@end
}
\@onlypreamble\AbstractOff
\newcommand*\AbstractOn{%
  \let\abstract\acs@abstract
  \let\endabstract\acs@endabstract
}
\@onlypreamble\AbstractOn
```

\acs@collect@toks \acs@collect@content \acs@collect@content

The content of the graphic TOC entry is processed using a method from amsmath *via* environ. The entire environment is gathered for typesetting in a box. First, some storage is needed.

```
\newtoks\acs@collect@toks
\newtoks\acs@collect@empty@toks
\newcommand*\acs@collect@begins{}
\newcommand*\acs@collect@content{}
```

\acs@collect \acs@collect@aux \acs@collect@begins@ \acs@collect@body

This is a \long version of \collect@body.

```
\newcommand\acs@collect[1]{%
  \acs@collect@toks{%
    \expandafter#1\expandafter{\the\acs@collect@toks}%
  }%
  \edef\acs@collect@content{%
    \the\acs@collect@toks
    \noexpand\end{\@currenvir}%
  }%
  \acs@collect@toks\acs@collect@empty@toks
  \def\acs@collect@begins{b}%
  \begingroup
    \expandafter\let\csname\@currenvir\endcsname\acs@collect@aux
    \edef\acs@collect@content{%
      \expandafter\noexpand\csname\@currenvir\endcsname
    }%
    \acs@collect@content
}
\newcommand*\acs@collect@aux{}
\long\def\acs@collect@aux#1\end#2{%
  \edef\acs@collect@begins{%
    \acs@collect@begins@#1\begin\end
    \expandafter\@gobble\acs@collect@begins
  }%
  \ifx\@empty\acs@collect@begins
    \endgroup
    \@checkend{#2}%
```

```
    \acs@collect@body{#1}%
  \else
    \acs@collect@body{#1\end{#2}}%
  \fi
  \acs@collect@content
}
\newcommand*\acs@collect@begins@{}
\long\def\acs@collect@begins@#1\begin#2{%
  \ifx\end#2\else
    b\expandafter\acs@collect@begins@
  \fi
}
\newcommand\acs@collect@body[1]{%
  \global\acs@collect@toks\expandafter{\the\acs@collect@toks#1}%
}
```

\acs@abstract@print

Delayed abstract printing works in a similar way, but with some formatting 'built-in'.

```
\newcommand\acs@abstract@print[1]{%
  \global\long\def\acs@abstract@text{%
    \if@twocolumn
      \@restonecoltrue\onecolumn
    \else
      \@restonecolfalse\newpage
    \fi
    \acs@section*{Abstract}%
    #1%
    \if@restonecol
      \twocolumn
    \else
      \newpage
    \fi
  }%
  \AtEndDocument{\acs@abstract@text}%
}
```

\acs@tocentry@print
\acs@tocentry@print@aux
\acs@tocentry@text

The same approach is taken for the graphical table of content printing. This is done in a box so that everything has a frame around it.

```
\newcommand{\acs@tocentry@print}[1]{%
  \gdef\acs@tocentry@text{\normalsize#1}%
  \AtEndDocument{%
    \if@twocolumn
      \@restonecoltrue\onecolumn
    \else
      \@restonecolfalse\newpage
    \fi
    \acs@tocentry@print@aux
    \if@restonecol
      \twocolumn
    \else
      \newpage
    \fi
  }%
}
```

```
\newcommand*{\acs@tocentry@print@aux}{%
  \begingroup
    \let\@startsection\acs@startsection@orig
    \acs@section*{\tocentryname}%
    \tocsize
    \sffamily
    \singlespacing
    \begin{center}
      \fbox
        {%
          \begin{minipage}{\acs@tocentry@width}
            \vbox to \acs@tocentry@height{\acs@tocentry@text}%
          \end{minipage}%
        }%
    \end{center}%
  \endgroup
}
\newcommand*\acs@tocentry@text{TOC ENTRY REQUIRED}
\newlength{\acs@tocentry@height}
\newlength{\acs@tocentry@width}
\setlength{\acs@tocentry@height}{1.75in}
\setlength{\acs@tocentry@width}{3.25in}
```

`tocentry` (*env.*) Actually creating the entry is pretty easy.

```
\newenvironment{tocentry}{\acs@collect\acs@tocentry@print}{}
```

`\tocentryname` A simple name macro.

```
\newcommand*\tocentryname{TOC Graphic}
```

`\tocsize` The font size for printing the TOC entry.

```
\newcommand*\tocsize{%
  \@setfontsize\tocsize\@viiipt\@ixpt
}
```

`\acs@type@list` `\acs@type@default` `\acs@type@check` Different journals allow different types of article. A list is set up here: different journals can then alter it. A check function is also provided along with a default.

```
\newcommand*\acs@type@list{article,communication,suppinfo}
\newcommand*\acs@type@default{article}
\newcommand*\acs@type@check{%
  \@tempswafalse
  \@for\@tempa:=\acs@type@list\do{%
    \ifx\@tempa\acs@manuscript
      \expandafter\@tempswatrue
    \fi
  }%
  \if@tempswa\else
    \acs@warning{%
      Invalid manuscript type \acs@manuscript:\MessageBreak
      changed to default type \acs@type@default
    }%
    \let\acs@manuscript\acs@type@default
  \fi
}
```

A few bits for older versions.

```
\newcommand*\acs@setkeys{\setkeys{acs}}
\let\acs@killabstract\AbstractOff
\let\acs@killsecs\SectionsOff
\newcommand*\acs@validtype[2][article]{%
  \def\acs@type@default{#1}%
  \def\acs@type@list{#2}%
}
```

`\acs@par` A saved paragraph.

```
\newcommand*\acs@par{}
\let\acs@par\par
```

`\acs@layout@shared` `acknowledgement` (*env.*) `suppinfo` (*env.*) Some code is used generally when setting up "press ready" layouts. There is quite a bit here, mainly layout related.

```
\newcommand*\acs@layout@shared{%
  \AtBeginDocument{\singlespacing}%
  \twocolumn
  \tolerance=2000\relax
  \emergencystretch=10pt\relax
  \geometry{
    letterpaper,
    top    = 12.7mm,
    bottom = 16.8mm,
    left   = 19.3mm,
    right  = 19.3mm
  }%
  \setlength{\columnsep}{8.1mm}%
  \setlength{\parindent}{3.3mm}%
  \renewenvironment{acknowledgement}{%
    \def\@tempa{acknowledgement}%
    \ifx\@currenvir\@tempa
      \let\par\relax
      \acksize
      \vspace{6pt}%
      \textbf{\acknowledgementname}%
    \else
      \acs@section*{\acknowledgementname}%
    \fi
  }{%
    \acs@par
  }%
}
```

`\acksize` `\suppsize` More sizes.

```
\newcommand*\acksize{\normalsize}
\newcommand*\suppsize{\normalsize}
```

`\acs@layout@nine` `\@xipt` `\acs@layout@ten` The class loads twelve point text. To reset it for print layouts, it is easiest to do things directly.

```
\newcommand*\acs@layout@nine{%
  \def\@xipt{11}%
  \long\def\normalsize{%
```

```
    \@setfontsize\normalsize\@ixpt\@xipt
  }%
  \normalsize
  \let\@listi\@listI
  \abovedisplayskip 5\p@ \@plus2\p@ \@minus 5\p@\relax
  \abovedisplayshortskip \z@ \@plus3\p@\relax
  \belowdisplayshortskip 3\p@ \@plus3\p@ \@minus 3\p@\relax
  \belowdisplayskip\abovedisplayskip\relax
  \abovecaptionskip 5\p@\relax
  \intextsep 7\p@ \@plus 2\p@ \@minus 2\p@\relax
}
\newcommand*\acs@layout@ten{%
  \long\def\normalsize{%
    \@setfontsize\normalsize\@xpt\@xiipt
  }%
  \normalsize
  \@setfontsize\normalsize\@xpt\@xiipt
  \let\@listi\@listI
  \abovedisplayskip 10\p@ \@plus2\p@ \@minus5\p@\relax
  \abovedisplayshortskip \z@ \@plus3\p@\relax
  \belowdisplayshortskip 6\p@ \@plus3\p@ \@minus3\p@\relax
  \belowdisplayskip \abovedisplayskip\relax
}
```

With all of the standard settings done, the journal configuration can be loaded.

```
\InputIfFileExists{achemso-\acs@journal.cfg}{}{%
  \acs@warning{%
    Unknown journal `\acs@journal':\MessageBreak
    using default configuration JACSAT%
  }%
  \input{achemso-jacsat.cfg}%
}
```

Suppress spurious warnings.

```
\let\@unusedoptionlist\@empty
```

\thepage Some changes that can always be applied if the manuscript type is appropriate: this saves some repetition in the configuration files.

```
\acs@type@check
\ifx\acs@manuscript\acs@manuscript@note
  \SectionsOff
\fi
\ifx\acs@manuscript\acs@manuscript@review
  \SectionsOn
  \SectionNumbersOn
\fi
\ifx\acs@manuscript\acs@manuscript@suppinfo
  \setkeys{acs}{maxauthors = 0}
  \def\thepage{S-\arabic{page}}
  \renewcommand*\thefigure{S\@arabic\c@figure}
  \renewcommand*\thescheme{S\@arabic\c@scheme}
  \renewcommand*\thetable{S\@arabic\c@table}
  \AtBeginDocument
    {%
```

```
      \renewcommand*\citenumfont[1]{S#1}%
      \renewcommand*\bibnumfmt[1]{(S#1)}%
    }
\fi
```

Set up two column layout.

```
\begingroup
  \def\@tempa{twocolumn}
  \ifx\acs@layout\@tempa
    \aftergroup\acs@layout@shared
  \fi
  \def\@tempa{two-column}
  \ifx\acs@layout\@tempa
    \aftergroup\acs@layout@shared
  \fi
\endgroup
⟨/class⟩
```

8.5 Late shared code

\citenumfont Changes to citations can now be made. The citation styles supplied here require natbib, which is loaded with the appropriate options. This part applies to the package as well as the class: it is here so that the load order is correct.

```
⟨*class | package⟩
\ifacs@super
  \RequirePackage[sort&compress,numbers,super]{natbib}
\else
  \RequirePackage[sort&compress,numbers,round]{natbib}
  \def\citenumfont{\textit}
\fi
\define@key{acs}{super}[true]{%
  \def\@tempa{#1}%
  \def\@tempb{true}%
  \ifx\@tempa\@tempb
    \setcitestyle{super,open={},close={}}%
    \renewcommand*\citenumfont{}%
  \else
    \setcitestyle{round}%
    \renewcommand*\citenumfont{\textit}
  \fi
}
\RequirePackage{natmove}
```

The mciteplus package allows the construction of lists of references with sub-letters. However, it might not be available, and so it is only loaded if available: the .bst files should work either way. There is also a patch to get cross-references correct with the modified \ref macro used here. The reason for ensuring that \@mciteNatbibCiteCmdList is defined is that it allows for the case where people prevent mciteplus being loaded using \ver@mciteplus.sty.

```
\IfFileExists{mciteplus.sty}{%
  \RequirePackage{mciteplus}
  \providecommand*{\@mciteNatbibCiteCmdList}{}%
  \edef\@tempa{\noexpand\in@{citenum}{\@mciteNatbibCiteCmdList}}%
```

```
  \@tempa
  \ifin@
  \else
    \edef\@mciteNatbibCiteCmdList{\@mciteNatbibCiteCmdList,citenum}%
  \fi
⟨*!package⟩
  \def\@mciteSubRef[##1]##2{\plainref{\@mcitereflabelprefix:##1:##2}}
⟨/!package⟩
}{}
```

`\acs@bibstyle` The next step is to sort out bibliography formatting. With both the package and the class, the bibliography style is determined without user intervention. The style is stored as it may need to be altered later.

```
\newcommand*\acs@bibstyle{achemso}
\ifacs@biochem
  \def\acs@bibstyle{biochem}
  \bibliographystyle{biochem}
\else
  \bibliographystyle{achemso}
\fi
```

`\acs@bibliographystyle` `\bibliographystyle` The original `\bibliographystyle` macro is now disabled: the underlying command is repeated otherwise problems cop up with chapterbib.

```
\newcommand*\acs@bibliographystyle{}
\let\acs@bibliographystyle\bibliographystyle
\def\bibliographystyle#1{%
  \acs@warning{\string\bibliographystyle\space ignored}%
  \expandafter\acs@bibliographystyle\expandafter{\acs@bibstyle}%
}
⟨/class | package⟩
```

8.6 Late package-only code

The notes2bib code may or may not be activated.

```
⟨*package⟩
\AtBeginDocument{%
  \@ifpackageloaded{notes2bib}{}{%
    \acs@niib@create
  }%
}
⟨/package⟩
```

8.7 Moving citations with natbib

The code for moving citations is created as a separate package, as the code needed is the same in both cases.

```
⟨*natmove⟩
\ProvidesPackage{natmove}
  [2010/01/15 v1.1a Automatic citation moving with natbib]
\RequirePackage{natbib}
```

\nmv@ifmtarg
\nmv@xifmtarg

To keep down dependence on other packages, the very short code block from ifmtarg is copied here with an internal name.

```
\newcommand*\nmv@ifmtarg{}
\newcommand*\nmv@xifmtarg{}
\begingroup
  \catcode`\Q 3\relax
  \long\gdef\nmv@ifmtarg#1{%
    \nmv@xifmtarg#1QQ\@secondoftwo\@firstoftwo\@nil
  }
  \long\gdef\nmv@xifmtarg#1#2Q#3#4#5\@nil{#4}
\endgroup
```

\ifnmv@cite

A flag is need to watch whether `\cite` or another macro is in use.

```
\newif\ifnmv@cite
```

\nmv@citex

Using the flag, either the new internal macro, or the natbib original, can be called.

```
\newcommand*\nmv@citex{%
  \ifnmv@cite
    \expandafter\nmv@citex@moving
  \else
    \expandafter\nmv@citex@nat
  \fi
}
```

\nmv@after

Later, the argument for `\cite` will need to be saved.

```
\newcommand*\nmv@after{}
```

\nmv@citex@moving

The new version of `\@citex` is needed that looks ahead of the citation using `\futurelet`. There are three arguments to `\@citex` when using natbib. Other than that, the trick used here is similar to that in cite.

```
\newcommand*\nmv@citex@moving{}
\def\nmv@citex@moving[#1][#2]#3{%
  \leavevmode
  \skip@\lastskip\relax
  \unskip
  \begingroup
    \def\nmv@after{\nmv@citex@nat[#1][#2]{#3}}%
    \global\nmv@citefalse
    \nmv@citex@get@next\relax
}
```

\nmv@citex@end

To get things right at the end.

```
\newcommand*\nmv@citex@end{%
    \nmv@after
  \endgroup
}
```

\nmv@citex@get@next
\nmv@citex@next

The next token on the input stack is saved into `\nmv@citex@next`, after gobbling up one token.

```
\newcommand*\nmv@citex@get@next[1]{%
  \futurelet\nmv@citex@next\nmv@citex@punct
}
```

`\nmv@citex@punct` `\nmv@citex@punct@undouble` `\nmv@citex@loop`

The working macro for moving the punctuation. This is very much like `\@citey` in the cite package. The initial assumption is that the loop will terminate, and so the recursion call will simply do the finalisation.

```
\newcommand*\nmv@citex@punct{%
  \let\nmv@citex@loop\nmv@citex@end
  \ifx\nmv@citex@next.\relax
    \ifnum\spacefactor<\nmv@citex@sfac\else
      \expandafter\expandafter\expandafter\nmv@citex@punct@undouble
    \fi
  \fi
  \expandafter\nmv@citex@punct@aux\natmovechars\@nil
  \nmv@citex@loop
}
\newcommand*\nmv@citex@punct@undouble{%
  \let\nmv@citex@next\relax
  \let\nmv@citex@loop\nmv@citex@get@next
}
\newcommand*\nmv@citex@loop{}
```

`\nmv@citex@punct@aux`

The final part of the punctuation moving system.

```
\newcommand*\nmv@citex@punct@aux[1]{%
  \ifx\nmv@citex@next#1\@empty
    #1%
    \let\nmv@citex@loop\nmv@citex@get@next
  \fi
  \ifx#1\@nil\else
    \expandafter\nmv@citex@punct@aux
  \fi
}
```

`\nmv@citex@sfac`

The value of the spacing factor after a full stop is used to signal doubled punctuation. For French spacing, a bit of patching is needed.

```
\mathchardef\nmv@citex@sfac3000\relax
\expandafter\def\expandafter\frenchspacing\expandafter{%
  \frenchspacing
  \mathchardef\nmv@citex@sfac1001\relax
  \sfcode`\.\nmv@citex@sfac
  \sfcode`\?\nmv@citex@sfac
  \sfcode`\!\nmv@citex@sfac
}
\ifnum\sfcode`\.=\@m
  \frenchspacing
\fi
```

`\nmv@citex@nat` `\nmv@activate` `\cite`

The swap has to be done at the beginning of the document. The internal flag from natbib is used, but under the circumstances we should be safe. `\cite` is also patched to make the system active.

```
\newcommand*\nmv@citex@nat{}
\newcommand*\nmv@activate{%
  \let\nmv@citex@nat\@citex
  \let\@citex\nmv@citex
  \let\nmv@cite\cite
  \renewcommand*{\cite}[2][]{%
```

```
    \nmv@ifmtarg{##1}{%
      \nmv@citetrue
      \nmv@cite{##2}%
    }{%
      \nmv@citefalse
      \nmv@cite[##1]{##2}}%
  }%
}
```

\nmv@natbib@detect So that natbib options can be set without worrying about load order, natmove doesn't require natbib. So a test is needed to see if it is actually loaded. This is done as a macro so that the effect can be changed by achemso.

```
\newcommand*\nmv@natbib@detect{%
  \@ifpackageloaded{natbib}{%
    \ifNAT@super
      \expandafter\nmv@activate
    \fi
  }{%
    \PackageInfo{natmove}{%
      The natbib package is not loaded.\MessageBreak
      Loading natmove will do nothing
    }%
  }%
}
\AtBeginDocument{\nmv@natbib@detect}
```

\natmovechars A user macro is needed for moving characters.

```
\newcommand*\natmovechars{.,;:}
⟨/natmove⟩
```

8.8 The configuration files

The configuration files for different journals are not very complex. Keeping everything separate simply helps with maintenance.

```
⟨*abmcb8⟩
\ProvidesFile{achemso-abmcb8.cfg}
  [2025-04-11 v3.13j achemso configuration: ACS Bio. Med. Chem. Au]
\def\acs@type@list{article,review,letter,perspective,suppinfo}
\setkeys{acs}{keywords = true}
⟨/abmcb8⟩
⟨*aeacb3⟩
\ProvidesFile{achemso-aeacb3.cfg}
  [2025-04-11 v3.13j achemso configuration: ACS Eng. Au]
\def\acs@type@list{article,review,letter,perspective,suppinfo}
\setkeys{acs}{keywords = true}
⟨/aeacb3⟩
⟨*aeacc4⟩
\ProvidesFile{achemso-aeacc4.cfg}
  [2025-04-11 v3.13j achemso configuration: ACS Env. Au]
\def\acs@type@list{article,review,letter,perspective,suppinfo}
\setkeys{acs}{keywords = true}
⟨/aeacc4⟩
⟨*amacgu⟩
```

```
\ProvidesFile{achemso-amacgu.cfg}
  [2025-04-11 v3.13j achemso configuration: ACS Mater. Au]
\def\acs@type@list{article,review,letter,perspective,suppinfo}
\setkeys{acs}{keywords = true}
⟨/amacgu⟩
⟨*amachv⟩
\ProvidesFile{achemso-amachv.cfg}
  [2025-04-11 v3.13j achemso configuration: ACS Meas. Au]
\def\acs@type@list{article,review,letter,perspective,suppinfo}
\setkeys{acs}{keywords = true}
⟨/amachv⟩
⟨*anaccx⟩
\ProvidesFile{achemso-anaccx.cfg}
  [2025-04-11 v3.13j achemso configuration: ACS Nanosci. Au]
\def\acs@type@list{article,review,letter,perspective,suppinfo}
\setkeys{acs}{keywords = true}
⟨/anaccx⟩
⟨*abmcb8⟩
\ProvidesFile{achemso-abmcb8.cfg}
  [2025-04-11 v3.13j achemso configuration: ACS Bio. Med. Chem. Au]
\def\acs@type@list{article,review,letter,perspective,suppinfo}
\setkeys{acs}{keywords = true}
⟨/abmcb8⟩
⟨*aoiab5⟩
\ProvidesFile{achemso-aoiab5.cfg}
  [2025-04-11 v3.13j achemso configuration: ACS Org. Inorg. Au]
\def\acs@type@list{article,review,letter,perspective,suppinfo}
\setkeys{acs}{keywords = true}
⟨/aoiab5⟩
⟨*apcach⟩
\ProvidesFile{achemso-apcach.cfg}
  [2025-04-11 v3.13j achemso configuration: ACS Phys. Bio. Med. Chem. Au]
\def\acs@type@list{article,review,letter,perspective,suppinfo}
\setkeys{acs}{keywords = true}
⟨/apcach⟩
⟨*appccd⟩
\ProvidesFile{achemso-appccd.cfg}
  [2025-04-11 v3.13j achemso configuration: ACS Polym. Au]
\def\acs@type@list{article,review,letter,perspective,suppinfo}
\setkeys{acs}{keywords = true}
⟨/appccd⟩
⟨*achre4⟩
\ProvidesFile{achemso-achre4.cfg}
  [2025-04-11 v3.13j achemso configuration: Acc. Chem. Res.]
\setkeys{acs}{biblabel = plain}
\def\acs@type@list{article,suppinfo}
\def\abstractname{Conspectus}
⟨/achre4⟩
⟨*aaembp⟩
\ProvidesFile{achemso-aaembp.cfg}
  [2025-04-11 v3.13j achemso configuration: ACS Appl. Electron. Mater.]
\def\acs@type@list{article,letter,suppinfo}
\setkeys{acs}{keywords = true}
⟨/aaembp⟩
```

```
⟨*aaemcq⟩
\ProvidesFile{achemso-aaemcq.cfg}
  [2025-04-11 v3.13j achemso configuration: ACS Appl. Energy Mater.]
\def\acs@type@list{article,letter,suppinfo}
\setkeys{acs}{keywords = true}
⟨/aaemcq⟩
⟨*aamick⟩
\ProvidesFile{achemso-aamick.cfg}
  [2025-04-11 v3.13j achemso configuration: ACS Appl. Mater. Interfaces]
\def\acs@type@list{article,letter,suppinfo}
\setkeys{acs}{keywords = true}
⟨/aamick⟩
⟨*aanmf6⟩
\ProvidesFile{achemso-aanmf6.cfg}
  [2025-04-11 v3.13j achemso configuration: ACS Appl. Nano Mater.]
\def\acs@type@list{article,letter,suppinfo}
\setkeys{acs}{keywords = true}
⟨/aanmf6⟩
⟨*aapmcd⟩
\ProvidesFile{achemso-aapmcd.cfg}
  [2025-04-11 v3.13j achemso configuration: ACS Appl. Polym. Mater.]
\def\acs@type@list{article,letter,suppinfo}
\setkeys{acs}{keywords = true}
⟨/aapmcd⟩
⟨*aastgj⟩
\ProvidesFile{achemso-aastgj.cfg}
  [2025-04-11 v3.13j achemso configuration: ACS Agri. Sci. Tech.]
\def\acs@type@list{account,article,letter,retraction,review,viewpoint,suppinfo}
\setkeys{acs}{keywords = true}
⟨/aastgj⟩
⟨*abseba⟩
\ProvidesFile{achemso-abseba.cfg}
  [2025-04-11 v3.13j achemso configuration: ACS Biomater. Sci. Eng.]
\setkeys{acs}{keywords = true}
\def\acs@type@list{article,review,suppinfo}
⟨/abseba⟩
⟨*accacs⟩
\ProvidesFile{achemso-accacs.cfg}
  [2025-04-11 v3.13j achemso configuration: ACS Catal.]
\setkeys{acs}{keywords = true}
\def\acs@type@list{article,letter,perspective,review,viewpoints,suppinfo}
\SectionNumbersOff
⟨/accacs⟩
⟨*acscii⟩
\ProvidesFile{achemso-acscii.cfg}
  [2025-04-11 v3.13j achemso configuration: ACS Central Sci.]
\def\acs@type@list{article,review,suppinfo}
\setkeys{acs}{doi = true}
\SectionNumbersOff
⟨/acscii⟩
⟨*acbcct⟩
\ProvidesFile{achemso-acbcct.cfg}
  [2025-04-11 v3.13j achemso configuration: ACS Chem. Biol.]
\setkeys{acs}{
```

```
  biblabel = fullstop,
  biochem  = true,
  super    = false
}
\def\acs@type@list{article,letter,review,suppinfo}
\SectionNumbersOff
⟨/acbcct⟩
⟨*acncdm⟩
\ProvidesFile{achemso-acncdm.cfg}
  [2025-04-11 v3.13j achemso configuration: ACS Chem. Neurosci.]
\def\acs@type@list{article,review,letter,suppinfo,viewpoint}
\setkeys{acs}{
  biblabel = fullstop,
  biochem  = true,
  keywords = true,
  super    = false
}
⟨/acncdm⟩
⟨*acsccc⟩
\ProvidesFile{achemso-acsccc.cfg}
  [2025-04-11 v3.13j achemso configuration: ACS Combinatorial Sci.]
\def\acs@type@list{article,letter,review,perspective,account,note,suppinfo}
\setkeys{acs}{keywords = true}
\SectionNumbersOff
⟨/acsccc⟩
⟨*aesccq⟩
\ProvidesFile{achemso-aesccq.cfg}
  [2025-04-11 v3.13j achemso configuration: ACS Earth Space Chem.]
\def\acs@type@list{article,suppinfo}
\setkeys{acs}{keywords = true}
⟨/aesccq⟩
⟨*aelccp⟩
\ProvidesFile{achemso-aelccp.cfg}
  [2025-04-11 v3.13j achemso configuration: ACS Energy Lett.]
\def\acs@type@list{letter,perspective,review,viewpoint,focus}
\SectionsOff
⟨/aelccp⟩
⟨*aeecco⟩
\ProvidesFile{achemso-aeecco.cfg}
  [2025-04-11 v3.13j achemso configuration: ACS ES&T Eng.]
\def\acs@type@list{article,feature,perspective,review,viewpoint}
\setkeys{acs}{keywords = true}
⟨/aeecco⟩
⟨*aewcaa⟩
\ProvidesFile{achemso-aewcaa.cfg}
  [2025-04-11 v3.13j achemso configuration: ACS ES&T Water]
\def\acs@type@list{article,feature,perspective,review,viewpoint}
\setkeys{acs}{keywords = true}
⟨/aewcaa⟩
⟨*afsthl⟩
\ProvidesFile{achemso-afsthl.cfg}
  [2025-04-11 v3.13j achemso configuration: ACS Food Sci. Tech.]
\def\acs@type@list{account,article,perspective,retraction,review,viewpoint}
\setkeys{acs}{keywords = true}
```

```
⟨/afsthl⟩
⟨*ascefj⟩
\ProvidesFile{achemso-ascefj.cfg}
  [2025-04-11 v3.13j achemso configuration: ACS Infect. Dis.]
\def\acs@type@list{article,letter,perspective,review,viewpoint,suppinfo}
\setkeys{acs}{keywords = true}
\SectionsOff
⟨/ascefj⟩
⟨*amlccd⟩
\ProvidesFile{achemso-amlccd.cfg}
  [2025-04-11 v3.13j achemso configuration: ACS Macro Lett.]
\def\acs@type@list{letter,suppinfo}
\SectionsOff
\setlength{\acs@tocentry@height}{4cm}
\setlength{\acs@tocentry@width}{8cm}
⟨/amlccd⟩
⟨*amlcef⟩
\ProvidesFile{achemso-amlcef.cfg}
  [2025-04-11 v3.13j achemso configuration: ACS Mater. Lett.]
\def\acs@type@list{letter,perspective,review,viewpoint,focus}
\SectionsOff
⟨/amlcef⟩
⟨*amclct⟩
\ProvidesFile{achemso-amclct.cfg}
  [2025-04-11 v3.13j achemso configuration: ACS Med. Chem. Lett.]
\def\acs@type@list{article,letter,perspective,suppinfo}
\SectionNumbersOff
⟨/amclct⟩
⟨*amrcda⟩
\ProvidesFile{achemso-amrcda.cfg}
  [2025-04-11 v3.13j achemso configuration: Acc. Mater. Res.]
\def\acs@type@list{account,article,editorial,retraction,suppinfo}
⟨/amrcda⟩
⟨*ancac3⟩
\ProvidesFile{achemso-ancac3.cfg}
  [2025-04-11 v3.13j achemso configuration: ACS Nano]
\setkeys{acs}{
  abbreviations = true,
  biblabel      = fullstop,
  etalmode      = truncate,
  maxauthors    = 20,
  keywords      = true
}
\def\acs@type@list{article,perspective,suppinfo}
\newcommand*{\latin}[1]{\emph{#1}}
\SectionNumbersOff
⟨/ancac3⟩
⟨*anmafm⟩
\ProvidesFile{achemso-anmafm.cfg}
  [2025-04-11 v3.13j achemso configuration: ACS Nano Med.]
\setkeys{acs}{
  abbreviations = true,
  articletitle  = true,
  biblabel      = fullstop,
```

```
  etalmode      = truncate,
  maxauthors    = 20,
  keywords      = true
}
\def\acs@type@list{editorial,viewpoint,perspective,review,articlen,suppinfo}
⟨/anmafm⟩
⟨*acsodf⟩
\ProvidesFile{achemso-acsodf.cfg}
  [2025-04-11 v3.13j achemso configuration: ACS Omega.]
\def\acs@type@list{article}
⟨/acsodf⟩
⟨*apchd5⟩
\ProvidesFile{achemso-apchd5.cfg}
  [2025-04-11 v3.13j achemso configuration: ACS Photon.]
\def\acs@type@list{article,letter,perspective,review,suppinfo}
\setkeys{acs}{keywords = true}
\ifx\acs@manuscript\acs@manuscript@letter
  \SectionNumbersOff
\fi
⟨/apchd5⟩
⟨*aidcbc⟩
\ProvidesFile{achemso-aidcbc.cfg}
  [2025-04-11 v3.13j achemso configuration: ACS Sensors]
\def\acs@type@list{article,letter,perspective,review,suppinfo}
\setkeys{acs}{keywords = true}
\SectionNumbersOff
⟨/aidcbc⟩
⟨*ascecg⟩
\ProvidesFile{achemso-ascecg.cfg}
  [2025-04-11 v3.13j achemso configuration: ACS Sustainable Chem Eng.]
\setkeys{acs}{
  biblabel = fullstop,
  keywords = true
}
\SectionNumbersOff
\def\acs@type@list{article,feature,letter,perspective,review,suppinfo}
⟨/ascecg⟩
⟨*asbcd6⟩
\ProvidesFile{achemso-asbcd6.cfg}
  [2025-04-11 v3.13j achemso configuration: ACS Synth. Biol.]
\setkeys{acs}{
  abbreviations = true,
  biblabel      = fullstop,
  biochem       = true,
  keywords      = true,
  super         = false
}
\def\acs@type@list{article,letter,note,tutorial,review,suppinfo}
⟨/asbcd6⟩
⟨*ancham⟩
\ProvidesFile{achemso-ancham.cfg}
  [2025-04-11 v3.13j achemso configuration: Anal. Chem.]
\def\acs@type@list{article,note,suppinfo}
\SectionNumbersOff
```

```
⟨/ancham⟩
⟨*bichaw⟩
\ProvidesFile{achemso-bichaw.cfg}
  [2025-04-11 v3.13j achemso configuration: Biochemistry]
\setkeys{acs}{
  abbreviations = true,
  biblabel      = brackets,
  biochem       = true,
}
\SectionNumbersOff
\def\acs@maketitle@extras@hook{%
  \par
  \acs@title@short@print
}
\g@addto@macro{\maketitle}{\newpage}
```

`\acs@author@fnsymbol` Some changes to do with footnotes: symbols are different and symbol number one can only be used for the title.

```
\def\acs@author@fnsymbol#1{%
  \ensuremath{%
    \ifcase #1 *\or
      \dagger\or
        \ddagger\or
          \S\or
            \parallel\or
              \perp\or
                \P\or
                  \|\or
                    \bot\or
                      \#\or
                        @\or
                          \triangle\or
                            \nabla\else
                              #1%
    \fi
  }%
}
\def\@maketitle@title@hook{%
  \ifnum\acs@footnote@cnt>\@ne
  \else
    \global\acs@footnote@cnt\@ne
  \fi
}
```

```
⟨/bichaw⟩
⟨*bcches⟩
\ProvidesFile{achemso-bcches.cfg}
  [2025-04-11 v3.13j achemso configuration: Bioconjugate Chem.]
\setkeys{acs}{
  biochem = true,
  super   = false
}
\SectionNumbersOff
⟨/bcches⟩
```

```
⟨*bomaf6⟩
\ProvidesFile{achemso-bomaf6.cfg}
  [2025-04-11 v3.13j achemso configuration: Biomacromolecules]
\def\acs@type@list{article,comment,communication,note,review,suppinfo}
\SectionNumbersOff
\AtEndOfClass{\SectionsOn}
⟨/bomaf6⟩
⟨*crtoec⟩
\ProvidesFile{achemso-crtoec.cfg}
  [2025-04-11 v3.13j achemso configuration: Chem. Res. Toxicol.]
\setkeys{acs}{
  abbreviations = true,
  biochem       = true,
  keywords      = true
}
\def\acs@maketitle@extras@hook{%
  \par
  \acs@title@short@print
}
\def\acs@type@list{%
  article,perspective,profile,review,suppinfo%
}
\SectionNumbersOff
⟨/crtoec⟩
⟨*chreay⟩
\ProvidesFile{achemso-chreay.cfg}
  [2025-04-11 v3.13j achemso configuration: Chem. Rev.]
  \setkeys{acs}{
    etalmode      = truncate,
    maxauthors    = 10
  }
\def\acs@type@default{review}
\def\acs@type@list{review}
```

The references section is numbered in *Chem. Rev.*

```
\floatstyle{plaintop}
\restylefloat{scheme}
\floatstyle{plain}
\def\bibsection{\acs@section{\refname}}
⟨/chreay⟩
⟨*cmatex⟩
%    \begin{macrocode}
\ProvidesFile{achemso-cmatex.cfg}
  [2025-04-11 v3.13j achemso configuration: Chem. Mater.]
\setkeys{acs}{keywords = true}
\SectionNumbersOff
\ifx\acs@manuscript\acs@manuscript@communication
  \AbstractOff
  \SectionsOff
\fi
⟨/cmatex⟩
⟨*cgdefu⟩
\ProvidesFile{achemso-cgdefu.cfg}
  [2025-04-11 v3.13j achemso configuration: Cryst. Growth Des.]
```

```
\def\acs@type@list{%
  article,communication,perspective,suppinfo%
}
\SectionNumbersOff
\ifx\acs@manuscript\acs@manuscript@communication
  \SectionsOff
\fi
\setlength{\acs@tocentry@height}{8.9 cm}
\setlength{\acs@tocentry@width}{4.6 cm}
⟨/cgdefu⟩
⟨*enfuem⟩
\ProvidesFile{achemso-enfuem.cfg}
  [2025-04-11 v3.13j achemso configuration: Energy Fuels]
\def\acs@type@list{article,review,suppinfo}
\SectionNumbersOff
⟨/enfuem⟩
⟨*esthag⟩
\ProvidesFile{achemso-esthag.cfg}
  [2025-04-11 v3.13j achemso configuration: Environ. Sci. Technol.]
\def\acs@type@list{article,feature,perspective,review,suppinfo}
\SectionNumbersOff
\setkeys{acs}{maxauthors = 0, keywords = true}
⟨/esthag⟩
⟨*estlcu⟩
\ProvidesFile{achemso-estlcu.cfg}
  [2025-04-11 v3.13j achemso configuration: Environ. Sci. Technol. Lett.]
\def\acs@type@list{letter,review,suppinfo}
\SectionNumbersOff
⟨/estlcu⟩
⟨*iecred⟩
\ProvidesFile{achemso-iecred.cfg}
  [2025-04-11 v3.13j achemso configuration: Ind. Eng. Chem. Res.]
\setkeys{acs}{biblabel = brackets}
\SectionNumbersOff
⟨/iecred⟩
⟨*inoraj⟩
\ProvidesFile{achemso-inoraj.cfg}
  [2025-04-11 v3.13j achemso configuration: Inorg. Chem.]
\SectionNumbersOff
\ifx\acs@manuscript\acs@manuscript@communication
  \AbstractOff
  \SectionsOff
\fi
\setkeys{acs}{doi = true}
⟨/inoraj⟩
⟨*jaaucr⟩
\ProvidesFile{achemso-jaaucr.cfg}
  [2025-04-11 v3.13j achemso configuration: JACS Au]
\def\acs@type@list{spotlight,account,article,communication,letter,perspective,suppinfo}
⟨/jaaucr⟩
⟨*jafcau⟩
\ProvidesFile{achemso-jafcau.cfg}
  [2025-04-11 v3.13j achemso configuration: J. Agric. Food Chem.]
\setkeys{acs}{keywords = true}
```

```
\def\acs@type@list{article,suppinfo}
\SectionNumbersOff
⟨/jafcau⟩
⟨*jceda8⟩
\ProvidesFile{achemso-jceda8.cfg}
  [2025-04-11 v3.13j achemso configuration: J. Chem. Ed.]
\setkeys{acs}{keywords = true}
\SectionNumbersOff
\def\acs@type@list{article,suppinfo}
\SectionNumbersOff
⟨/jceda8⟩
⟨*jceaax⟩
\ProvidesFile{achemso-jceaax.cfg}
  [2025-04-11 v3.13j achemso configuration: J. Chem. Eng. Data]
\def\acs@type@list{article,suppinfo}
\def\refname{Literature Cited}
\SectionNumbersOff
⟨/jceaax⟩
⟨*jcisd8⟩
\ProvidesFile{achemso-jcisd8.cfg}
  [2025-04-11 v3.13j achemso configuration: J. Chem. Inf. Model.]
\def\acs@type@list{article,suppinfo}
\SectionNumbersOff
⟨/jcisd8⟩
⟨*jctcce⟩
\ProvidesFile{achemso-jctcce.cfg}
  [2025-04-11 v3.13j achemso configuration: J. Chem. Theory Comput.]
\def\acs@type@list{article,suppinfo}
\setkeys{acs}{maxauthors = 0}
⟨/jctcce⟩
⟨*jmcmar⟩
\ProvidesFile{achemso-jmcmar.cfg}
  [2025-04-11 v3.13j achemso configuration: J. Med. Chem.]
\def\acs@type@list{article,letter,perspective,suppinfo}
\SectionNumbersOff
\setlength{\acs@tocentry@height}{4.45cm}
\setlength{\acs@tocentry@width}{8.25cm}
⟨/jmcmar⟩
⟨*jnprdf⟩
\ProvidesFile{achemso-jnprdf.cfg}
  [2025-04-11 v3.13j achemso configuration: J. Nat. Prod.]
\renewcommand*{\abstractname}{ABSTRACT}
\renewcommand*{\acknowledgementname}{ACKNOWLEDGEMENT}
\renewcommand*{\suppinfoname}{ASSOCIATED CONTENT}
\SectionNumbersOff
\renewcommand*{\acs@abstract}{%
  \quotation
  \textbf{\abstractname :}%
}
\AbstractOn
\ifx\acs@manuscript\acs@manuscript@communication
  \AbstractOff
  \SectionsOff
\fi
```

```
\def\refname{REFERENCES}
\captionsetup{
  labelfont = bf,
  labelsep  = period
}
⟨/jnprdf⟩
⟨*joceah⟩
\ProvidesFile{achemso-joceah.cfg}
  [2025-04-11 v3.13j achemso configuration: J. Org. Chem.]
\ifx\acs@manuscript\acs@manuscript@communication
  \AbstractOff
  \SectionsOff
\fi
\floatstyle{plaintop}
\restylefloat{scheme}
\floatstyle{plain}
\renewcommand*\acs@type@list{article,communication,note,perspective,review,suppinfo}
⟨/joceah⟩
```

```
⟨*jpcafh⟩
\ProvidesFile{achemso-jpcafh.cfg}
  [2025-04-11 v3.13j achemso configuration: J. Phys. Chem. A]
\def\acs@type@list{article,suppinfo}
\setkeys{acs}{
  etalmode     = truncate,
  maxauthors   = 10
}
\SectionNumbersOff
\captionsetup[table]{labelfont=bf,textfont=bf}
```

Title

```
\g@addto@macro{\maketitle}{\newpage}
⟨/jpcafh⟩
⟨*jpcbfk⟩
\ProvidesFile{achemso-jpcbfk.cfg}
  [2025-04-11 v3.13j achemso configuration: J. Phys. Chem. B]
\def\acs@type@list{article,suppinfo}
\setkeys{acs}{
  etalmode     = truncate,
  maxauthors   = 10
}
\SectionNumbersOff
\captionsetup[table]{labelfont=bf,textfont=bf}
\g@addto@macro{\maketitle}{\newpage}
⟨/jpcbfk⟩
⟨*jpccck⟩
\ProvidesFile{achemso-jpccck.cfg}
  [2025-04-11 v3.13j achemso configuration: J. Phys. Chem. C]
\def\acs@type@list{article,suppinfo}
\setkeys{acs}{
  etalmode     = truncate,
  maxauthors   = 10
}
\SectionNumbersOff
\captionsetup[table]{labelfont=bf,textfont=bf}
```

```
\g@addto@macro{\maketitle}{\newpage}
⟨/jpccck⟩
⟨*jpclcd⟩
\ProvidesFile{achemso-jpclcd.cfg}
  [2025-04-11 v3.13j achemso configuration: J. Phys. Chem. Lett.]
\setkeys{acs}{
  etalmode   = truncate,
  maxauthors = 10
}
\def\acs@type@default{letter}
\def\acs@type@list{letter}
\SectionNumbersOff
\captionsetup[table]{labelfont=bf,textfont=bf}
\g@addto@macro{\maketitle}{\newpage}
\renewcommand{\acs@tocentry@print}[1]{%
  \gdef\acs@tocentry@text{#1}%
}
\renewcommand*{\acs@abstract@extras}{%
  \begingroup
    \acs@tocentry@print@aux
  \endgroup
  \acs@keywords@print
  \newpage
}
\setlength\acs@tocentry@height{2in}
\setlength\acs@tocentry@width{2in}
⟨/jpclcd⟩
⟨*jprobs⟩
\ProvidesFile{achemso-jprobs.cfg}
  [2025-04-11 v3.13j achemso configuration: J. Proteome Res.]
\setkeys{acs}{keywords = true}
\def\acs@type@list{article,review,suppinfo}
\SectionNumbersOff
⟨/jprobs⟩
```

The *J. Am. Chem. Soc.* configuration is rather more complicated as there is the need to construct a "galley-like" layout for communications.

```
⟨*jacsat⟩
\ProvidesFile{achemso-jacsat.cfg}
  [2025-04-11 v3.13j achemso configuration: J. Am. Chem. Soc.]
\SectionNumbersOff
\ifx\acs@manuscript\acs@manuscript@communication\else
  \expandafter\endinput
\fi
```

Everything from here onward applies only to communications. Some adjustments are now made using the existing tools.

```
\setkeys{acs}{
  email  = true,
  layout = twocolumn
}
\SectionsOff
\acs@layout@nine
```

abstract (*env.*) The abstract appears at the start of the document, with lines around it.s

```
\renewenvironment{abstract}{%
  \hrule
  \vspace{2 mm}%
  \sffamily
  \noindent
  \emph{\textbf{Abstract:}}%
}{%
  \vspace{2 mm}%
  \hrule
  \vspace{6 mm}%
}
```

\acksize \affilsize \authorsize \emailsize \capsize \refsize \suppsize \titlesize

To keep things logical, the size macros are given names related to their function. Some of these are new, some are defined in the class.

```
\def\affilsize{%
  \@setfontsize\affilsize\@ixpt\@xpt
}
\def\acksize{%
  \@setfontsize\acksize\@ixpt\@xipt
}
\def\authorsize{%
  \@setfontsize\authorsize{10.5}{12.5}%
}
\newcommand*\capsize{%
  \@setfontsize\capsize\@viiipt\@ixpt
}
\def\emailsize{%
  \@setfontsize\emailsize\@viiipt{15}%
}
\newcommand*\refsize{%
  \@setfontsize\refsize{7.5}{7.5}%
}
\def\suppsize{%
  \@setfontsize\suppsize{8.5}{10.5}%
}
\def\titlesize{%
  \@setfontsize\titlesize\@xiipt{13}%
}
\let\footnotesize\refsize
\let\captionfont\capsize
```

\emailfont A slight font change.

```
\def\emailfont{\sffamily}
```

\ps@plain \ps@jacs

The header styles are done the hard way, to keep down the number of packages loaded.

```
\def\ps@plain{%
  \let\@mkboth\@gobbletwo
  \let\@oddhead\@empty
  \def\@oddfoot{%
    \reset@font
    \sffamily
```

```
    \textbf{\thepage}%
    \hfil
  }%
  \let\@evenhead\@empty
  \let\@evenfoot\@oddfoot
}
\def\ps@acs{%
  \def\@oddfoot{%
    \reset@font
    \sffamily
    \textbf{\thepage}%
    \hfil
  }%
  \def\@evenfoot{%
    \reset@font
    \hfil
    \sffamily
    \textbf{\thepage}%
  }%
  \def\@oddhead{}%
  \let\@evenhead\@oddhead
}
\pagestyle{acs}
```

`\acs@space@pre@title` `\acs@space@post@title` `\acs@space@post@author` `\acs@space@post@address` `\acs@space@post@email`

Length adjustments for the title.

```
\setlength\acs@space@pre@title{16mm}
\setlength\acs@space@post@title{0mm}
\setlength\acs@space@post@author{0mm}
\setlength\acs@space@post@address{0mm}
\setlength\acs@space@post@email{-1mm}
\setlength\acs@maketitle@width{152.4mm}
```

`\acs@contact@details`

Contact details are different here.

```
\def\acs@contact@details{%
  Received \today; E-mail: \acs@email@list
}
```

`\acs@maketitle@extras`

No keywords or abbreviations for *J. Am. Chem. Soc.*.

```
\let\acs@maketitle@extras\relax
```

Getting the floats correct is a difficult task "by hand"; using the caption package makes this a lot easier.

```
\floatstyle{plaintop}
\restylefloat{scheme}
\floatstyle{plain}
\DeclareCaptionLabelSeparator{perquad}{.\quad}
\captionsetup{
  singlelinecheck = off,
  labelfont       = {bf,it,sf},
  textfont        = sf,
  labelsep        = perquad
}
\captionsetup[figure]{textfont=rm}
```

\acs@table \table A patch or \table.

```
\newcommand*\acs@table{}
\let\acs@table\table
\def\table{%
  \capsize
  \acs@table
}
```

The bibliography has to be adjusted.

```
\AtBeginDocument{%
  \def\bibsection{%
    \@startsection
      {section}
      {1}
      {\z@}{\z@}{2.5mm}%
      {\normalfont\acksize\bfseries}
      {\hrule\nobreak\vspace{1.2mm}\noindent\refname}%
  }%
  \let\bibfont\refsize
  \setlength{\bibhang}{0.61cm}%
  \setlength{\bibsep}{0mm}%
}
⟨/jacsat⟩
⟨*langd5⟩
\ProvidesFile{achemso-langd5.cfg}
  [2025-04-11 v3.13j achemso configuration: Langmuir]
\def\acs@type@list{article,letter,suppinfo}
\SectionNumbersOff
⟨/langd5⟩
⟨*mamobx⟩
\ProvidesFile{achemso-mamobx.cfg}
  [2025-04-11 v3.13j achemso configuration: Macromolecules]
\SectionNumbersOff
\setkeys{acs}{doi = true}
⟨/mamobx⟩
⟨*mpohbp⟩
\ProvidesFile{achemso-mpohbp.cfg}
  [2025-04-11 v3.13j achemso configuration: Mol. Pharm.]
\setkeys{acs}{keywords = true}
\def\acs@type@list{article,suppinfo}
\SectionNumbersOff
⟨/mpohbp⟩
⟨*nalefd⟩
\ProvidesFile{achemso-nalefd.cfg}
  [2025-04-11 v3.13j achemso configuration: Nano Lett.]
\setkeys{acs}{keywords = true}
\def\acs@type@default{letter}
\def\acs@type@list{letter}
\SectionNumbersOff
⟨/nalefd⟩
⟨*orlef7⟩
\ProvidesFile{achemso-orlef7.cfg}
  [2025-04-11 v3.13j achemso configuration: Org. Lett.]
\def\acs@type@default{communication}
```

```
\def\acs@type@list{communication}
\SectionNumbersOff
\setkeys{acs}{layout = twocolumn}
\RequirePackage{xcolor}
\definecolor{orglett}{RGB}{128,0,0}
⟨/orlef7⟩
⟨*oprdfk⟩
\ProvidesFile{achemso-oprdfk.cfg}
  [2025-04-11 v3.13j achemso configuration: Org. Proc. Res. Dev.]
\def\acs@type@list{article,highlight,review,suppinfo}
\SectionNumbersOff
⟨/oprdfk⟩
⟨*orgnd7⟩
\ProvidesFile{achemso-orgnd7.cfg}
  [2025-04-11 v3.13j achemso configuration: Organometallics]
\SectionNumbersOff
⟨/orgnd7⟩
```

9 Index

Numbers written in italic refer to the page where the corresponding entry is described; numbers underlined refer to the code line of the definition; numbers in roman refer to the code lines where the entry is used.

Symbols

\! 1524
\# 833, 1873
\. 1522, 1526
\? 1523
\@Esphack 1155
\@abbreviations 546, 557, 559
\@address@i 853
\@affil@i 889
\@afterindentfalse 1060, 1071
\@afterindenttrue 1059, 1070
\@arabic 1382, 1383, 1384
\@auxout 284
\@bsphack 174, 1145
\@checkend 1205
\@citex 223, 224, 1531, 1532
\@currenvir 1154, 1186, 1191, 1193, 1323
\@dblarg 1063, 1064
\@empty 332, 557, 564, 658, 675, 694, 962, 965, 971, 985, 1002, 1203, 1370, 1510, 2196, 2203
\@esphack 206, 210
\@evenfoot . . 2204, 2213
\@evenhead . . 2203, 2220
\@firstofone . . . 123, 127
\@firstoftwo 28, 393, 501, 1464
\@floatboxreset 1041, 1042
\@fnsymbol 611, 664, 879, 880, 896
\@for 770, 1285
\@gobble 325, 1201
\@gobbletwo 2195
\@ifclassloaded 10
\@ifpackageloaded 1450, 1544
\@ifstar 1063, 1074, 1078
\@ifundefined . . 148, 451
\@ignorefalse 1157
\@ixpt . . . 1279, 1340, 2168, 2171, 2177
\@keywords 546, 564, 566
\@listI 1343, 1357
\@listi 1343, 1357
\@m 1063, 1526
\@makeother 183
\@maketitle 597
\@maketitle@title@hook 597, 1881
\@mciteNatbibCiteCmdList . . 1423, 1424, 1428
\@mciteSubRef 1431
\@mcitereflabelprefix 1431
\@minus . . 1344, 1346, 1349, 1358, 1360
\@mkboth 2195
\@namedef 144
\@nameuse 38, 103, 117, 688, 738, 819, 863, 873, 903, 923, 987, 993
\@ne . . . 343, 346, 370, 400, 418, 423, 439, 481, 507, 684, 702, 723, 745, 755, 848, 860, 861, 867, 868, 883, 887, 890, 899, 910, 919, 942, 945, 946, 981, 1882, 1884
\@nil 1464, 1466, 1501, 1514
\@oddfoot 2197, 2204, 2207
\@oddhead 2196, 2219, 2220

\@onelevel@sanitize 192
\@onlypreamble 336, 352, 385, 492, 527, 536, 545, 550, 555, 1092, 1096, 1102, 1108, 1170, 1175
\@plus 1344, 1345, 1346, 1349, 1358, 1359, 1360
\@restonecolfalse 1226, 1244
\@restonecoltrue 1224, 1242
\@roman 344, 347, 371, 374, 377, 403, 424, 426, 441, 444, 447, 451, 452, 455, 457, 478, 482, 484, 510, 521, 530, 539, 688, 716, 734, 738, 746, 765, 771, 782, 788, 863, 869, 873, 903, 911, 923, 944, 951, 983, 984, 987, 993
\@secondoftwo 30, 395, 503, 1464
\@secpenalty . 1062, 1073
\@sect 1063, 1064
\@setfontsize 1279, 1340, 1353, 1356, 2168, 2171, 2174, 2177, 2180, 2183, 2186, 2189
\@ssect 1074, 1075
\@startsection 1055, 1066, 1090, 1094, 1113, 1256, 2252
\@tempa 190, 191, 192, 194, 198, 203, 262, 268, 271, 274, 278, 279, 388, 404, 414, 467, 494, 511, 770, 772, 960, 962, 963, 966, 967, 971, 973, 978, 985, 986, 990, 991, 1002, 1003, 1004, 1153, 1154, 1285, 1286, 1322, 1323, 1392, 1393, 1396, 1397, 1410, 1412, 1424, 1425
\@tempb 402, 404, 509, 511, 960, 963, 965, 967, 1411, 1412
\@tempcnta 390, 400, 403, 407, 415, 427, 440, 453, 458, 468, 472, 479, 496, 507, 510, 514, 677, 684, 688, 697, 703, 710, 716, 734, 738, 747, 765, 771, 782, 788, 857, 859, 860, 863, 865, 866, 867, 869, 873, 881, 890, 899, 900, 903, 905, 919, 920, 930, 942, 943, 944, 951, 979, 981, 982, 983, 984, 987, 993
\@tempcntb . 416, 423, 424, 426, 430, 469, 882, 910, 911, 914, 923, 931, 933, 945, 946
\@tempskipa 1058, 1060, 1062, 1069, 1071, 1073
\@tempswafalse 389, 495, 1284
\@tempswatrue 405, 512, 1287
\@thanks 655, 658, 661, 662, 675
\@title 329, 613
\@unusedoptionlist 1370
\@viiipt 1279, 2177, 2180
\@xiipt . 1353, 1356, 2189
\@xipt 1337, 2171
\@xpt . . . 1353, 1356, 2168
\\ 650
\{ 185
\} 186
\^ 197
\| 831, 1871

A

\abbreviations 546
\abovecaptionskip 1348
\abovedisplayshortskip 1345, 1359
\abovedisplayskip 1344, 1347, 1358, 1361
\abstract 1132, 1167, 1172
abstract (env.) . . . 2156
\abstractname 1623, 2044, 2050
\AbstractOff 5, 1166, 1299, 1942, 1989, 2054, 2067
\AbstractOn 5, 1166, 2052
acknowledgement (env.) 5, 1118, 1307
\acknowledgementname 1119, 1124, 1327, 1329, 2045
\acksize 1325, 1335, 2167, 2256
\acs@abbreviations@print 556, 1014
\acs@abstract 1131, 1172, 2048
\acs@abstract@end 1131, 1168
\acs@abstract@extras 1009, 2126
\acs@abstract@iffalse 1131
\acs@abstract@print 1221
\acs@abstract@start 1131, 1167
\acs@abstract@text 1222, 1236
\acs@activate@biblabel 108
\acs@address@list 631, 839
\acs@address@list@auxi 839
\acs@address@list@auxii 839
\acs@affil@alt@cnt 337, 481, 482, 485, 497, 514, 726, 754, 849, 886, 893
\acs@affil@alt@list 894, 909

\acs@affil@alt@lista@aux 909
\acs@affil@cnt 337, 346, 349, 370, 371, 374, 377, 407, 418, 425, 439, 440, 441, 444, 447, 723, 745, 789, 840, 848, 859, 866, 883, 905
\acs@affil@ifdup 367, 386, 436
\acs@affil@ifdup@aux 386, 417, 470
\acs@affil@list . . . 877
\acs@affil@list@aux 877
\acs@affil@marker@cnt . . 340, 746, 748, 751, 755, 756, 759, 772, 773, 777, 787, 789, 790, 794, 858, 861, 862, 868, 872, 887, 888, 889, 900, 901, 902, 920, 921, 922
\acs@affil@swap 368, 412
\acs@affil@swap@aux 412
\acs@alsoaffil@find 434
\acs@altaffil@foot@aux 912, 918
\acs@altaffil@ifdup 477, 493
\acs@altaffil@ifdup@aux 493
\acs@author@affil 691, 732
\acs@author@affil@also 692, 764
\acs@author@affil@also@aux 764
\acs@author@affil@alt 693, 781
\acs@author@affil@alt@aux 781
\acs@author@affil@aux 732
\acs@author@cnt 337, 343, 344, 348, 366, 430, 435, 451, 452, 455, 457, 475, 478, 484, 520, 521, 529, 530, 538, 539, 599, 678, 697, 702, 703, 709, 710, 914, 943, 982
\acs@author@fnsymbol 611, 664, 722, 751, 759, 777, 794, 797, 862, 872, 880, 1862
\acs@author@fnsymbol@aux 797
\acs@author@fnsymbol@loop 797
\acs@author@fnsymbol@loop@aux@i 797
\acs@author@fnsymbol@loop@aux@ii 797
\acs@author@fnsymbol@loop@aux@m 797
\acs@author@fnsymbol@loop@aux@@ 797
\acs@author@fnsymbol@symbol 797
\acs@author@footnotes . . 682, 721, 749, 750, 757, 758, 774, 775, 791, 792
\acs@author@list 624, 676
\acs@author@list@and 686, 701
\acs@author@list@comma 689, 701
\acs@author@list@main 679, 682
\acs@author@star 690, 715
\acs@author@star@aux 715
\acs@autonote 213
\acs@bib@file 201, 247, 260, 261, 265, 288
\acs@bib@message 247, 261
\acs@bib@name . . 247, 260
\acs@bib@write 253
\acs@bib@write@aux 253
\acs@biblabel@brackets 129
\acs@biblabel@fullstop 129
\acs@biblabel@period 129
\acs@biblabel@plain 129
\acs@bibliography 168, 291
\acs@bibliographystyle 1441
\acs@bibstyle 1434, 1445
\acs@collect . 1180, 1276
\acs@collect@aux . 1180
\acs@collect@begins 1178, 1189, 1199, 1201, 1203
\acs@collect@begins@ 1180
\acs@collect@body 1180
\acs@collect@content 1176, 1176, 1184, 1192, 1195, 1210
\acs@collect@empty@toks 1177, 1188
\acs@collect@toks . 1176, 1181, 1182, 1185, 1188, 1219
\acs@contact@details 639, 671, 2229
\acs@email@list 672, 926, 2230
\acs@email@list@aux 926
\acs@email@list@font 926
\acs@endabstract 1131, 1173
\acs@etal@firstonly 96
\acs@etal@truncate . 96
\acs@etal@truncatefalse 106
\acs@etal@truncatetrue 107
\acs@floatboxreset 1041
\acs@fnsymbol@org . 877
\acs@footnote@cnt 340, 615, 748, 756, 773, 790, 858, 888, 901, 921, 1882, 1884
\acs@iffalse 1165
\acs@ifundefined 24, 35, 98, 112, 716, 733, 765, 782, 869, 911, 944, 983, 984
\acs@journal 80, 1363, 1365
\acs@keyval@bool . . 34, 51, 54, 57, 60, 63, 66, 69, 72, 75, 78

\acs@keywords@print . . . 556, 1018, 2130
\acs@killabstract 1299
\acs@killsecs 1300
\acs@layout 80, 1393, 1397
\acs@layout@nine 1337, 2155
\acs@layout@shared . . 1307, 1394, 1398
\acs@layout@ten . . 1337
\acs@maketitle@extras 1022, 2232
\acs@maketitle@extras@hook . . 1022, 1857, 1912
\acs@maketitle@suppinfo 613, 648
\acs@maketitle@width 575, 606, 2228
\acs@manuscript 80, 649, 1286, 1292, 1295, 1372, 1375, 1379, 1809, 1941, 1953, 1988, 2053, 2066, 2147
\acs@manuscript@communication 138, 1941, 1953, 1988, 2053, 2066, 2147
\acs@manuscript@letter 138, 1809
\acs@manuscript@note 138, 1372
\acs@manuscript@review 138, 1375
\acs@manuscript@suppinfo 138, 649, 1379
\acs@maxauthors . 80, 278
\acs@niib@after@text 154, 162, 172, 207, 211
\acs@niib@create 143, 313, 1451
\acs@niib@no@write 178, 209
\acs@niib@text 155, 163, 173
\acs@niib@write 176, 181
\acs@niib@write@aux@i 181
\acs@niib@write@aux@ii 181
\acs@nmv@activate . 213
\acs@number@list 673, 957
\acs@number@list@aux@i 957
\acs@number@list@aux@ii 957
\acs@par 1305, 1332
\acs@section 1048, 1104, 1119, 1122, 1127, 1228, 1257, 1329, 1933
\acs@sections@none 1077, 1098, 1099, 1100
\acs@sections@none@aux 1077
\acs@setkeys 1298
\acs@space@post@address 575, 634, 2223
\acs@space@post@author 575, 627, 2223
\acs@space@post@email 575, 642, 2223
\acs@space@post@title 575, 619, 2223
\acs@space@pre@title 575, 604, 2223
\acs@startsection 1056, 1094
\acs@startsection@alt 1067, 1090
\acs@startsection@orig . . 1048, 1113, 1256
\acs@subsection 1048, 1105
\acs@subsubsection 1048, 1106
\acs@table 2244
\acs@title@footnote 612, 653
\acs@title@footnote@check 614, 653
\acs@title@short 329, 572
\acs@title@short@print . . . 556, 1859, 1914
\acs@tocentry@height 1265, 1272, 1274, 1752, 1956, 2038, 2133
\acs@tocentry@print . . 1238, 1276, 2123
\acs@tocentry@print@aux 1238, 2128
\acs@tocentry@text 1238, 2124
\acs@tocentry@width 1264, 1273, 1275, 1753, 1957, 2039, 2134
\acs@type@check 1281, 1371
\acs@type@default . 1281, 1302, 1928, 2118, 2287, 2294
\acs@type@list 1281, 1303, 1561, 1567, 1573, 1579, 1585, 1591, 1597, 1603, 1609, 1615, 1622, 1628, 1634, 1640, 1646, 1652, 1658, 1665, 1671, 1677, 1689, 1695, 1706, 1713, 1719, 1725, 1731, 1737, 1743, 1750, 1758, 1764, 1770, 1782, 1797, 1802, 1807, 1816, 1828, 1840, 1845, 1900, 1916, 1929, 1949, 1962, 1968, 1975, 1997, 2003, 2011, 2017, 2024, 2030, 2036, 2073, 2078, 2090, 2102, 2119, 2140, 2267, 2280, 2288, 2295, 2304
\acs@validtype . . . 1301
\acs@warning 5, 22, 36, 99, 113, 354, 360, 382, 462, 489, 524, 533, 542, 600, 736, 843, 934, 1085, 1141, 1291, 1364, 1444
\active 1136, 1146
\addpenalty . 1062, 1073
\addvspace . . 1062, 1073
\advance 343, 346, 370, 400, 418, 423, 439, 481, 507, 684, 748, 756, 773, 789, 790, 860, 861, 867, 868, 888, 899, 901, 910, 919, 921, 942, 945, 981
\affilfont 591, 630

\affiliation 4, 365
\affilsize 587, 629, 2167
\aftergroup . . . 393, 395, 1010, 1394, 1398
\alsoaffiliation 4, 434
\altaffiliation . 4, 474
\and 4, 353
\arabic 1381
articletitle (option) . 6
\AtBeginDocument 213, 258, 291, 297, 328, 1047, 1308, 1385, 1449, 1555, 2250
\AtEndDocument 287, 1236, 1240
\AtEndOfClass 122, 123, 1125, 1902
\AtEndOfPackage 126, 127
\author 4, 342
\authorfont 591, 591, 622
\authorsize 587, 621, 2167

B

\begin 605, 606, 607, 1200, 1213, 1261, 1264, 1936
\begingroup . 26, 182, 345, 387, 413, 466, 476, 660, 663, 928, 958, 1109, 1112, 1135, 1190, 1255, 1391, 1461, 1482, 2127
\belowdisplayshortskip 1346, 1360
\belowdisplayskip 1347, 1361
\bfseries 594, 2256
\bibfont 2259
\bibhang 2260
biblabel (option) 7
\bibliography 291
\bibliographystyle . . 1437, 1439, 1441
\bibnote 7, 143, 143, 232, 243
\bibnotemark 7, 143
\bibnotetext 7, 143
\bibnumfmt 129, 130, 131, 132, 1388
\bibsection 1124, 1933, 2251
\bibsep 2261
biochem (option) 7
biochemistry (option) . 7
\bot 832, 1872

C

\c@figure 1382
\c@footnote 615
\c@scheme 1383
\c@table 1384
\capsize 2167, 2247
\captionfont 2192
\captionsetup . 2058, 2084, 2096, 2108, 2121, 2237, 2243
\catcode 185, 186, 197, 1136, 1137, 1138, 1139, 1146, 1147, 1462
\centering 1043
chaptertitle (option) . 6
chart (env.) 5, 1028
\chartname 1034
\citation 285
\cite . . 154, 159, 225, 226, 237, 238, 1529
\citenum 232, 243
\citenumfont . 1387, 1402
\ClassWarning 5
\closeout 288
\columnsep 1319
\csname 27, 344, 347, 371, 374, 377, 403, 424, 426, 441, 444, 447, 452, 455, 457, 478, 482, 484, 510, 521, 530, 539, 734, 746, 771, 788, 951, 1191, 1193

D

\dagger 827, 1865
\ddagger 828, 1866
\DeclareCaptionLabelSeparator 2236
\DeclareRobustCommand 152, 157, 161
\def . . . 85, 88, 91, 94, 129, 130, 131, 132, 149, 154, 190, 191, 194, 214, 293, 342, 353, 359, 388, 401, 414, 467, 494, 508, 597, 685, 821, 929, 978, 980, 1042, 1126, 1153, 1189, 1198, 1213, 1222, 1302, 1303, 1322, 1338, 1339, 1352, 1381, 1392, 1396, 1407, 1410, 1411, 1431, 1436, 1443, 1478, 1483, 1519, 1561, 1567, 1573, 1579, 1585, 1591, 1597, 1603, 1609, 1615, 1622, 1623, 1628, 1634, 1640, 1646, 1652, 1658, 1665, 1671, 1677, 1689, 1695, 1706, 1713, 1719, 1725, 1731, 1737, 1743, 1750, 1758, 1764, 1770, 1782, 1797, 1802, 1807, 1816, 1828, 1840, 1845, 1857, 1862, 1881, 1900, 1912, 1916, 1928, 1929, 1933, 1949, 1962, 1968, 1975, 1997, 2003, 2011, 2017, 2018, 2024, 2030, 2036, 2057, 2078, 2090, 2102, 2118, 2119, 2140, 2167, 2170, 2173, 2179, 2185, 2188, 2193, 2194, 2197, 2206, 2207, 2213, 2219, 2229, 2246, 2251, 2267, 2280, 2287, 2288, 2294, 2295, 2304
\define@key 50, 53, 56, 59, 62, 65, 68, 71, 74, 77, 84, 87, 90, 93, 97, 108, 111, 1409
\definecolor 2299
\do 183, 771, 1285
\document 1027
\doi 6
doi (option) 6
\dospecials 184
\doublespacing 328

E

\edef 262, 986, 990, 1184, 1192, 1199, 1424, 1428
\else 29, 177, 217, 269, 272, 276, 280, 324, 376, 381, 394, 406, 446, 461, 488, 502, 513, 523, 532, 541, 557, 564, 659, 678, 694, 702, 725, 753, 805, 814, 816, 842, 852, 885, 943, 964, 965, 971, 982, 989, 1002, 1061, 1072, 1160, 1207, 1214, 1225, 1232, 1243, 1249, 1290, 1328, 1405, 1415, 1427, 1438, 1472, 1497, 1514, 1876, 1883, 2147
\email 4, 519
email (option) 3
\emailfont 591, 637, 2193
\emailsize 587, 636, 2167
\emergencystretch 1311
\emph 1783, 2161
\end 643, 644, 645, 1148, 1186, 1198, 1200, 1208, 1214, 1266, 1268
\endabstract . . 1009, 1134, 1168, 1173
\endcsname 27, 344, 348, 371, 374, 377, 403, 424, 426, 441, 444, 447, 453, 456, 457, 479, 483, 485, 510, 521, 530, 539, 734, 747, 771, 788, 951, 1191, 1193
\endgroup 26, 193, 350, 397, 420, 471, 487, 666, 668, 939, 975, 1115, 1117, 1155, 1164, 1204, 1269, 1400, 1467, 1489, 2129
\endinput 15, 2148
\ensuremath . . 798, 1863
environments:
  abstract 2156
  acknowledgement 5, 1118, 1307
  chart 5, 1028
  graph 5, 1028
  scheme 5, 1028
  suppinfo 5, 1118, 1307
  tocentry 5, 1276
etalmode (option) 6
\everypar . . . 1061, 1072
\expandafter . 26, 27, 28, 30, 176, 178, 193, 194, 216, 218, 255, 323, 325, 344, 347, 371, 374, 377, 401, 402, 405, 408, 424, 426, 431, 441, 444, 447, 452, 455, 471, 472, 478, 482, 484, 498, 501, 503, 508, 509, 512, 515, 521, 530, 539, 639, 661, 679, 698, 746, 770, 787, 799, 804, 806, 815, 841, 851, 884, 894, 906, 915, 949, 950, 954, 998, 1110, 1161, 1182, 1191, 1193, 1201, 1215, 1219, 1287, 1445, 1471, 1473, 1498, 1501, 1515, 1519, 1546, 2148

F

\fax 4, 528
\fbox 1262
\fi . 31, 169, 179, 219, 256, 269, 272, 276, 280, 326, 334, 379, 383, 396, 409, 410, 429, 432, 449, 463, 490, 499, 504, 516, 517, 525, 534, 543, 561, 568, 601, 640, 651, 669, 680, 696, 699, 705, 706, 712, 713, 728, 729, 761, 762, 807, 814, 816, 837, 844, 848, 849, 854, 891, 892, 895, 907, 916, 938, 948, 955, 969, 970, 974, 995, 999, 1006, 1016, 1020, 1057, 1060, 1062, 1068, 1071, 1073, 1148, 1159, 1162, 1209, 1216, 1227, 1234, 1245, 1251, 1288, 1296, 1330, 1374, 1378, 1390, 1395, 1399, 1408, 1418, 1429, 1440, 1474, 1499, 1500, 1513, 1516, 1528, 1547, 1811, 1878, 1885, 1944, 1955, 1991, 2056, 2069, 2149
\floatname 1029, 1031, 1033
\floatplacement 1037, 1038
\floatstyle . . 1039, 1930, 1932, 2070, 2072, 2233, 2235
\footnote 612
\footnotemark 654
\footnotesize 2191
\footnotetext 655, 889, 902, 922
\frenchspacing 623, 1519, 1520, 1527
\futurelet 1492

G

\g@addto@macro 655, 1009, 1026, 1027, 1861, 2085, 2097, 2109, 2122
\gdef . . 330, 331, 344, 371, 374, 377, 441, 444, 447, 482, 521, 530, 539, 547, 552,

1140, 1152, 1239, 1463, 1466, 2124
\geometry 1312
\global 333, 343, 370, 439, 481, 615, 1157, 1219, 1222, 1484, 1884
graph (env.) 5, 1028
\graphname 1034

H

\hfil . . . 2201, 2211, 2215
\hrule . . 2157, 2164, 2257

I

\if@filesw 175, 254
\if@ignore 1156
\if@nobreak . 1061, 1072
\if@noskipsec 1057, 1068
\if@restonecol 1230, 1247
\if@tempswa 392, 500, 1290
\if@twocolumn 1223, 1241
\ifacs@abbreviations 41, 1013
\ifacs@articletitle 41, 269
\ifacs@biochem . 41, 1435
\ifacs@chaptertitle 41, 272
\ifacs@doi 41, 276
\ifacs@email 41, 638
\ifacs@etal@truncate 96, 280
\ifacs@hyperref . 41, 322
\ifacs@keywords 41, 1017
\ifacs@super . . . 41, 1403
\ifcase 826, 1864
\ifdim 1060, 1071
\iffalse 1150, 1165
\IfFileExists 1421
\ifin@ 1426
\ifNAT@super . . 215, 1545
\ifnmv@cite . 1468, 1470
\ifnum 166, 366, 407, 424, 430, 435, 475, 497, 514, 520, 529, 538, 599, 678, 697, 702, 703, 709, 710, 723, 726, 745, 754, 803, 814, 816, 840, 847, 848, 849, 859, 866, 883, 886, 893, 905, 914, 933, 943, 946, 982, 1497, 1526, 1882
\ifx . 27, 332, 373, 404, 443, 511, 557, 564, 649, 658, 694, 962, 965, 971, 985, 1002, 1154, 1203, 1214, 1286, 1323, 1372, 1375, 1379, 1393, 1397, 1412, 1496, 1510, 1514, 1809, 1941, 1953, 1988, 2053, 2066, 2147
\ignorespaces 1158
\immediate . 201, 260, 261, 265, 284, 288
\in@ 1424
\input 1368
\InputIfFileExists 1363
\intextsep 1349
\itshape 591

J

\jobname . . 168, 252, 294
journal (option) 3

K

\keywords 546

L

\LARGE 590
\large 588
\lastskip 1480
\latin 6, 297, 1783
layout (option) 3
\leavevmode 1057, 1068, 1479
\let 122, 123, 126, 127, 162, 183, 223, 224, 225, 237, 292, 333, 596, 611, 612, 664, 675, 879, 880, 896, 960, 963, 1041, 1047, 1049, 1051, 1053, 1055, 1066, 1090, 1094, 1098, 1099, 1100, 1104, 1105, 1106, 1113, 1132, 1134, 1148, 1149, 1167, 1168, 1172, 1173, 1191, 1256, 1295, 1299, 1300, 1306, 1324, 1343, 1357, 1370, 1442, 1495, 1505, 1506, 1512, 1531, 1532, 1533, 2191, 2192, 2195, 2196, 2203, 2204, 2220, 2232, 2245, 2259
\LoadClass 4
\long 190, 1198, 1213, 1222, 1339, 1352, 1463, 1466
\loop 859, 866

M

\maketitle . . . 1026, 1027, 1861, 2085, 2097, 2109, 2122
\MakeUppercase . . . 1004
manuscript (option) . . . 3
\mathchardef . 1518, 1521
maxauthors (option) . . . 6
\MessageBreak 12, 18, 100, 114, 356, 362, 737, 935, 1142, 1292, 1365, 1550

N

\nabla 836, 1876
\natmovechars 8, 1501, 1556
\newcommand 5, 22, 25, 34, 80, 81, 82, 83, 106, 107, 120, 129, 130, 131, 132, 138, 139, 140, 141, 142, 143, 165, 172, 173, 181, 189, 200, 209, 222, 236, 248, 252, 253, 259, 365, 386, 399, 412, 422, 434, 465, 474, 493, 506, 519, 528, 537, 546, 549, 551, 554, 556, 563, 570, 587, 588, 589, 590, 591, 592, 593, 594, 595, 647, 648, 653, 657,

671, 676, 682, 683, 701, 708, 715, 720, 732, 744, 764, 769, 781, 786, 797, 802, 810, 813, 818, 824, 825, 839, 846, 856, 877, 878, 898, 909, 918, 926, 927, 941, 957, 977, 1008, 1012, 1022, 1025, 1034, 1035, 1036, 1046, 1048, 1050, 1052, 1054, 1056, 1067, 1077, 1084, 1089, 1093, 1097, 1103, 1124, 1130, 1131, 1133, 1165, 1166, 1171, 1178, 1179, 1180, 1197, 1212, 1218, 1221, 1238, 1254, 1271, 1277, 1278, 1281, 1282, 1283, 1298, 1301, 1305, 1307, 1335, 1336, 1337, 1351, 1434, 1441, 1459, 1460, 1469, 1476, 1477, 1487, 1491, 1494, 1504, 1508, 1509, 1529, 1530, 1543, 1556, 1783, 2176, 2182, 2244
\newcount 337, 338, 339, 340, 341
\newcounter 148
\newenvironment 1118, 1121, 1276
\newfloat 1028, 1030, 1032
\newif 41, 42, 43, 44, 45, 46, 47, 48, 49, 96, 1468
\newlength 575, 577, 579, 581, 583, 585, 1272, 1273
\newpage . . 602, 1226, 1233, 1244, 1250, 1861, 2085, 2097, 2109, 2122, 2131
\newtoks 1176, 1177
\newwrite 247
\nmv@activate 1529, 1546
\nmv@after 1476, 1483, 1488
\nmv@cite 225, 229, 237, 240, 1533, 1537, 1540
\nmv@citefalse 1484, 1539
\nmv@citetrue . 228, 1536
\nmv@citex 224, 1469, 1532
\nmv@citex@end 1487, 1495
\nmv@citex@get@next 1485, 1491, 1506, 1512
\nmv@citex@loop 1494, 1512
\nmv@citex@moving 1471, 1477
\nmv@citex@nat 223, 1473, 1483, 1529
\nmv@citex@next 1491, 1496, 1505, 1510
\nmv@citex@punct 1492, 1494
\nmv@citex@punct@aux 1501, 1509
\nmv@citex@punct@undouble 1494
\nmv@citex@sfac 1497, 1518
\nmv@ifmtarg 227, 239, 1459, 1535
\nmv@natbib@detect 213, 1543
\nmv@xifmtarg 1459
\nobreak 2257
\nocite . . . 167, 231, 242
\noexpand . 664, 1113, 1186, 1193, 1424
\noindent . . . 2160, 2257
\normalfont 2256
\normalsize 587, 589, 1239, 1335, 1336, 1339, 1340, 1342, 1352, 1353, 1355, 1356
\null 603
\number 799

O

\onecolumn . . 1224, 1242
\openout 260
options:
articletitle 6
biblabel 7
biochem 7
biochemistry 7
chaptertitle 6
doi 6
email 3
etalmode 6
journal 3
layout 3
manuscript 3
maxauthors 6
super 6
\or 826, 827, 828, 829, 830, 831, 832, 833, 834, 835, 1864, 1865, 1866, 1867, 1868, 1869, 1870, 1871, 1872, 1873, 1874, 1875

P

\P 829, 1870
\p@ 1344, 1345, 1346, 1348, 1349, 1358, 1359, 1360
\PackageInfo 11, 17, 1549
\PackageWarning 22
\pagestyle . . . 598, 2222
\par . . . 560, 567, 573, 617, 625, 632, 853, 863, 873, 972, 1015, 1019, 1058, 1069, 1306, 1324, 1858, 1913
\parallel 1868
\parindent 1320
\perp 1869
\phone 4, 528
\plainref . . . 1046, 1431
\printbibnotes 143
\ProcessOptionsX . . 137
\protected@edef 721, 749, 757, 774, 791, 966, 1003
\providecommand 298, 1423
\ProvidesClass 2
\ProvidesFile 1559, 1565, 1571, 1577, 1583, 1589, 1595, 1601, 1607, 1613, 1619, 1626, 1632, 1638, 1644, 1650, 1656, 1662, 1668, 1675, 1682, 1693, 1704, 1711, 1717, 1723, 1729,

1735, 1741, 1748, 1756, 1762, 1768, 1773, 1787, 1800, 1805, 1814, 1821, 1831, 1843, 1849, 1889, 1898, 1905, 1922, 1937, 1947, 1960, 1966, 1973, 1979, 1985, 1995, 2000, 2007, 2015, 2022, 2028, 2034, 2042, 2064, 2076, 2088, 2100, 2112, 2137, 2144, 2265, 2271, 2277, 2284, 2292, 2302, 2308

\ProvidesPackage 8, 1456

\ps@acs 595, 2206

\ps@jacs 2194

\ps@plain 596, 2194

Q

\Q 1462

\quad 2236

\quotation 2049

R

\ref 1046

\refname . 1127, 1933, 2018, 2057, 2257

\refsize 2167, 2259

\relax 27, 162, 166, 185, 186, 197, 260, 288, 343, 365, 366, 370, 373, 390, 400, 407, 415, 416, 418, 423, 425, 430, 434, 435, 439, 440, 443, 468, 469, 472, 475, 481, 496, 497, 507, 514, 520, 529, 538, 599, 677, 678, 684, 697, 702, 703, 709, 710, 723, 726, 745, 747, 748, 754, 755, 756, 772, 773, 788, 789, 790, 881, 882, 883, 886, 887, 888, 890, 893, 899, 900, 901, 905, 910, 914, 919, 920, 921, 930, 931, 933, 942, 943, 945, 946, 979, 981, 982, 1058, 1069, 1137, 1138, 1139, 1147, 1310, 1311, 1324, 1344, 1345, 1346, 1347, 1348, 1349, 1358, 1359, 1360, 1361, 1462, 1480, 1485, 1496, 1505, 1518, 1521, 2232

\renewcommand 226, 238, 329, 1382, 1383, 1384, 1387, 1388, 1414, 1417, 1534, 2044, 2045, 2046, 2048, 2073, 2123, 2126

\renewenvironment 1321, 2156

\repeat 864, 875

\RequirePackage . 33, 314, 315, 323, 1404, 1406, 1420, 1422, 1458, 2298

\reset@font 2198, 2208, 2214

\restylefloat . 1040, 1931, 2071, 2234

\romannumeral 815

S

\S 830, 1867

scheme (env.) 5, 1028

\schemename 1034

\section . . . 558, 565, 571, 1049, 1098, 1104

\SectionNumbersOff 5, 1089, 1672, 1679, 1690, 1708, 1765, 1784, 1810, 1818, 1827, 1846, 1856, 1895, 1901, 1919, 1940, 1952, 1963, 1969, 1976, 1982, 1987, 2004, 2010, 2012, 2019, 2025, 2037, 2047, 2083, 2095, 2107, 2120, 2141, 2146, 2268, 2273, 2281, 2289, 2296, 2305, 2310

\SectionNumbersOn 5, 1089, 1377

\SectionsOff . 5, 1097, 1300, 1373, 1720, 1745, 1751, 1759, 1943, 1954, 1990, 2055, 2068, 2154

\SectionsOn 5, 1097, 1376, 1902

\setcitestyle 1413, 1416

\setkeys 109, 133, 302, 1298, 1380, 1562, 1568, 1574, 1580, 1586, 1592, 1598, 1604, 1610, 1616, 1621, 1629, 1635, 1641, 1647, 1653, 1659, 1664, 1670, 1678, 1684, 1696, 1707, 1714, 1726, 1732, 1738, 1744, 1775, 1789, 1808, 1817, 1823, 1833, 1851, 1891, 1907, 1924, 1939, 1970, 1981, 1992, 2002, 2009, 2031, 2079, 2091, 2103, 2114, 2139, 2150, 2274, 2279, 2286, 2297

\setlength . 576, 578, 580, 582, 584, 586, 1274, 1275, 1319, 1320, 1752, 1753, 1956, 1957, 2038, 2039, 2133, 2134, 2223, 2224, 2225, 2226, 2227, 2228, 2260, 2261

\sf 926

\sfcode 1522, 1523, 1524, 1526

\sffamily 592, 594, 672, 1259, 2159, 2193, 2199, 2209, 2216

\singlespacing 1260, 1308

\skip@ 1480

\space . 203, 263, 268, 271, 274, 275, 278, 355, 361, 687, 704, 967, 1444

\spacefactor 1497

\stepcounter . . . 153, 158
\string 202, 203, 204, 266, 282, 285, 355, 361, 1444
\subsection 1051, 1099, 1105
\subsubsection 1053, 1100, 1106
super (option) 6
suppinfo (env.) 5, 1118, 1307
\suppinfoname 1122, 1124, 2046
\suppsize . . . 1335, 2167

T

\table 2244
\tableofcontents . 1109
\textbf 1327, 2050, 2161, 2200, 2210, 2217
\textit 1407, 1417
\textsuperscript . . 695
\textwidth 586
\thanks 4, 353
\the 150, 166, 349, 427, 453, 458, 472, 479, 485, 665, 1114, 1182, 1185, 1219
\thebibnote 143
\thefigure 1382
\thepage 1371, 2200, 2210, 2217
\thescheme 1383
\thetable 1384
\title 4, 329
\titlefont 591, 609
\titlesize 587, 610, 2167
tocentry (env.) . . 5, 1276
\tocentryname 1257, 1277
\tocsize 1258, 1278
\today 2230
\toks@ 661, 665, 1110, 1114
\tolerance 1310
\triangle 835, 1875
\tw@ 709
\twocolumn 1231, 1248, 1309

U

\unskip 1481
\url 949
\UrlFont 929

V

\value 150, 166
\vbox 1265
\vspace 604, 619, 627, 634, 642, 1326, 2158, 2163, 2165, 2257

W

\write . 201, 261, 265, 284

X

\xdef 347, 426, 452, 455, 478, 484, 662, 1111

Z

\z@ 166, 366, 390, 415, 416, 435, 468, 469, 475, 496, 497, 520, 529, 538, 599, 677, 678, 722, 726, 754, 840, 849, 850, 857, 865, 881, 882, 886, 893, 930, 931, 933, 979, 1060, 1071, 1345, 1359, 2255

10 References

[1] This note text will be in the bibliography.

[2] Coghill, A. M., Garson, L. R., Eds. *The ACS Style Guide*, 3rd ed.; Oxford University Press, Inc. and The American Chemical Society: New York, 2006.
