# Supplementary figures and images for: Identification of Orbital Pumping from Spin Pumping and Rectification Effects
Source: Nano Lett. 2025 Aug 26;25(36):13462–7. doi: 10.1021/acs.nanolett.5c02641 (PMC12426985; doi:10.1021/acs.nanolett.5c02641)

(a)

Nb(4)/Ni(10)

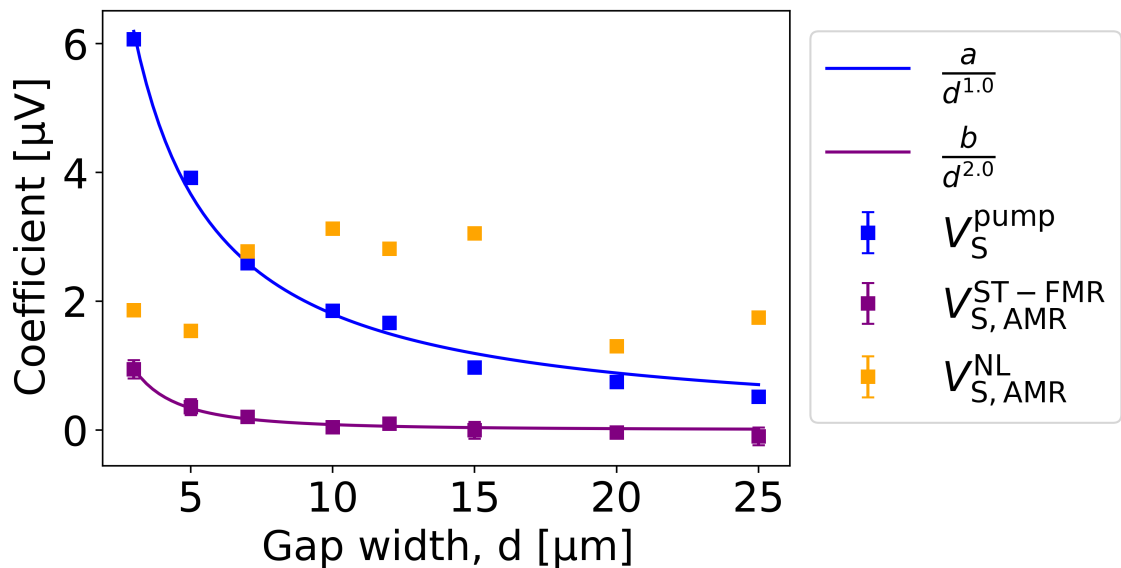

(b)

Ru(4)/Ni(10)

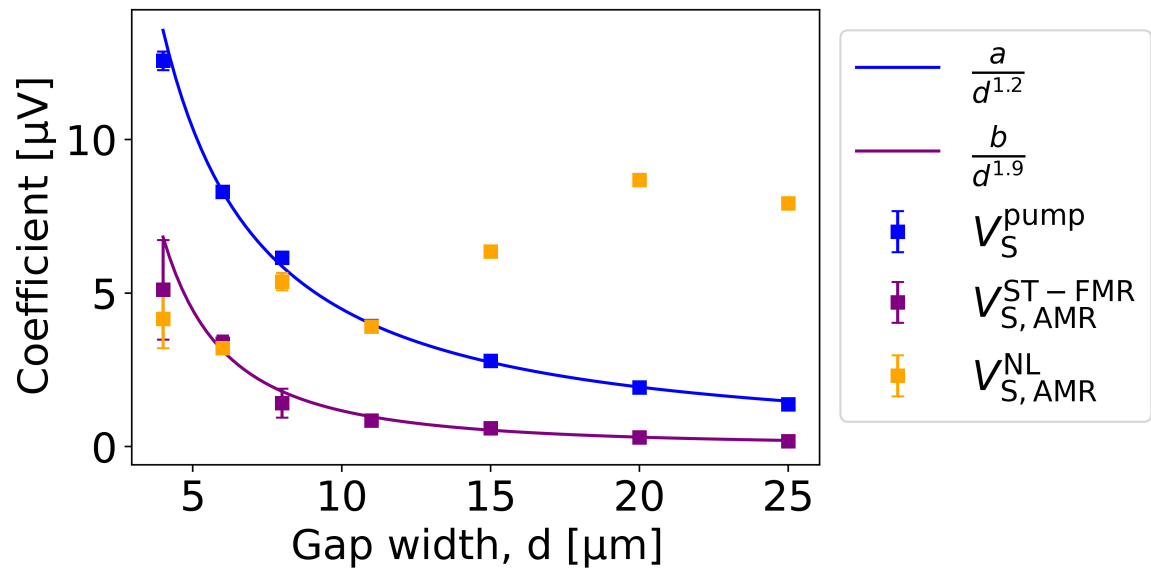

Supplement: Supplementary file 2 [file nl5c02641_si_002.zip › SMGapdependence.pdf]

(a) Ru(4)/Ni(10)

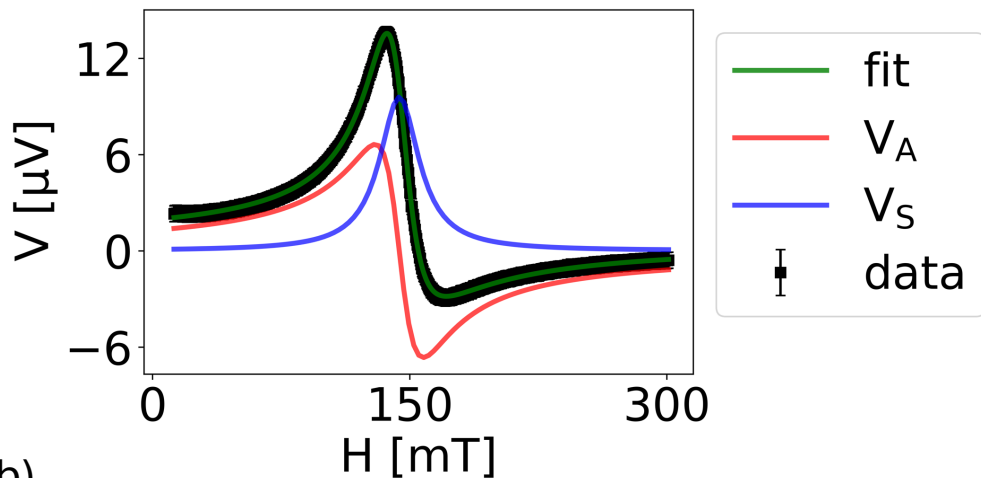

(b)

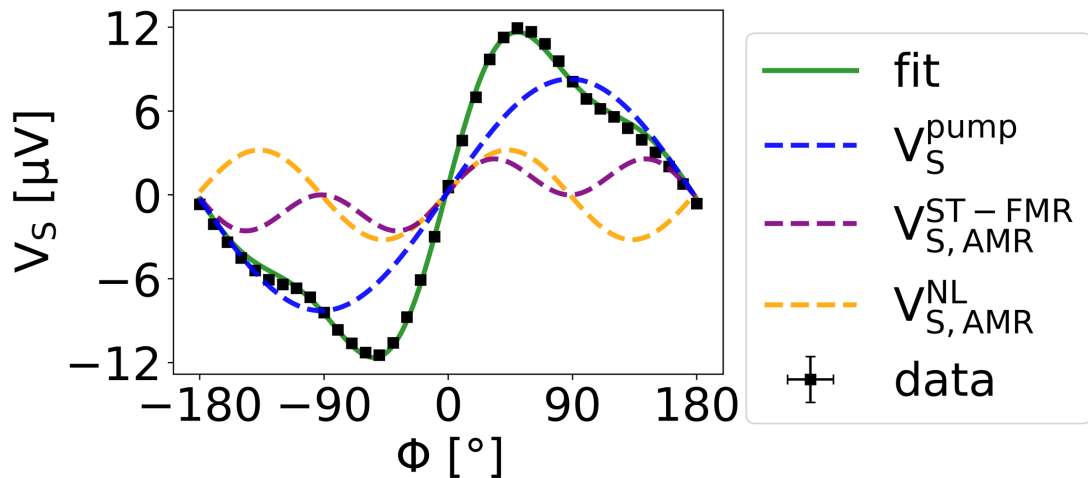

Supplement: Supplementary file 2 [file nl5c02641_si_002.zip › RutheniumData.pdf]

Nb(4)/Ni(6)

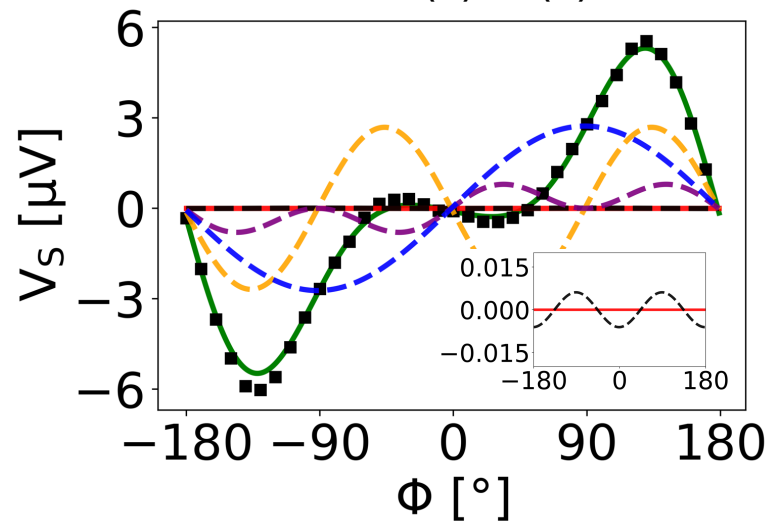

Nb(4)/FeCoB(8)

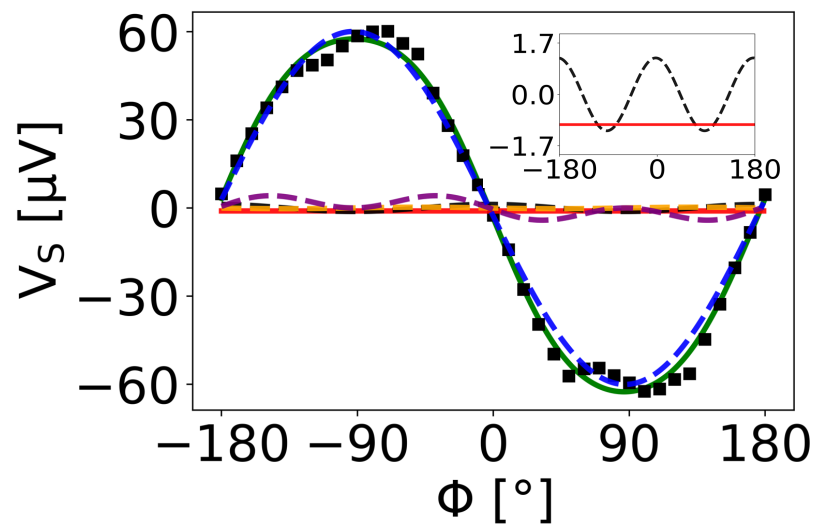

Pt(4)/Ni(6)

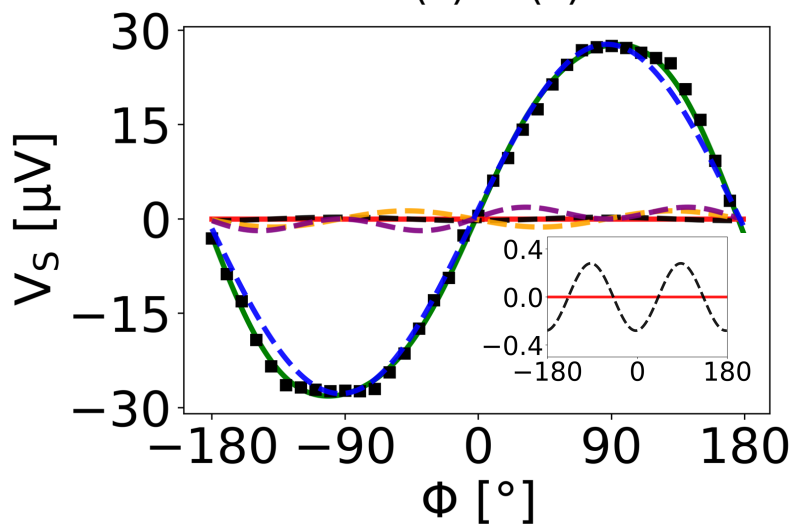

Pt(4)/FeCoB(8)

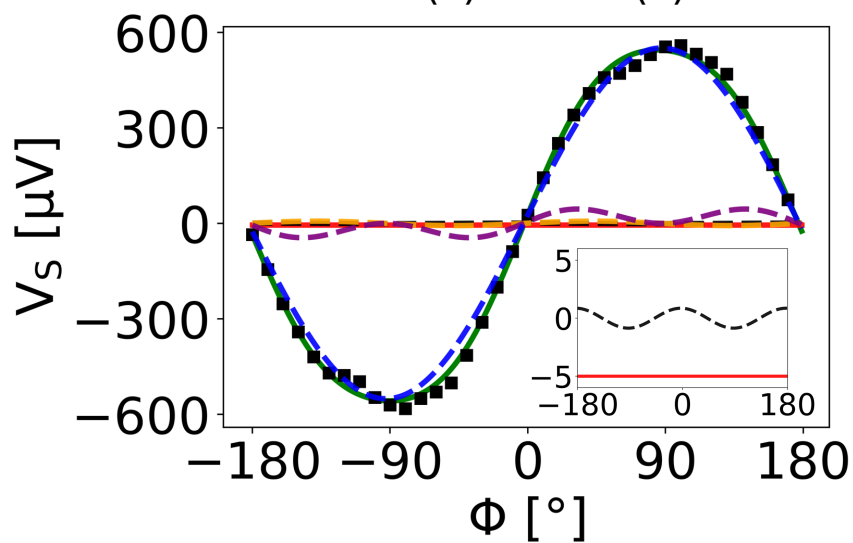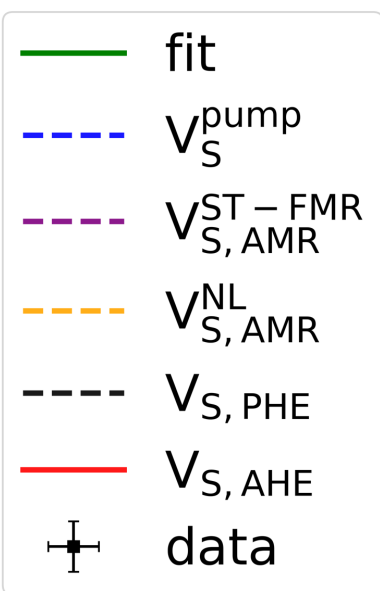

Supplement: Supplementary file 2 [file nl5c02641_si_002.zip › 5components_noitalic.pdf]

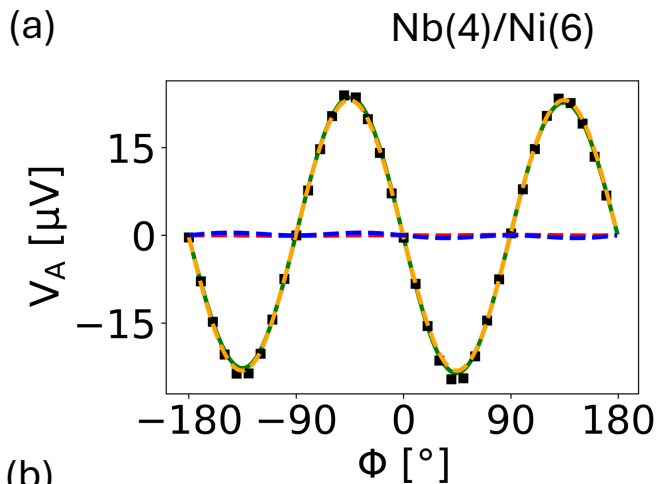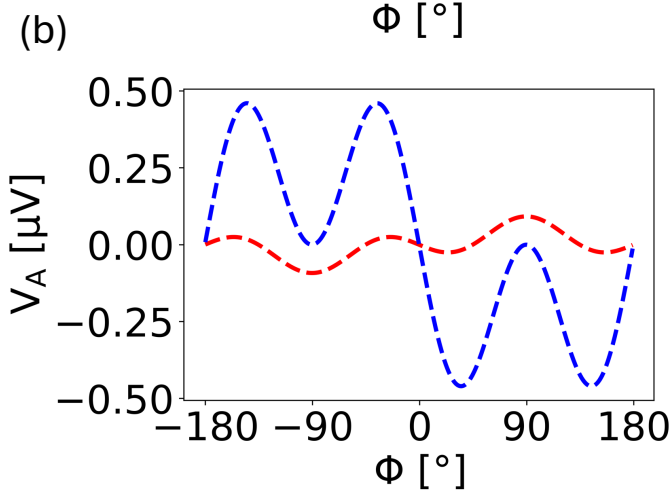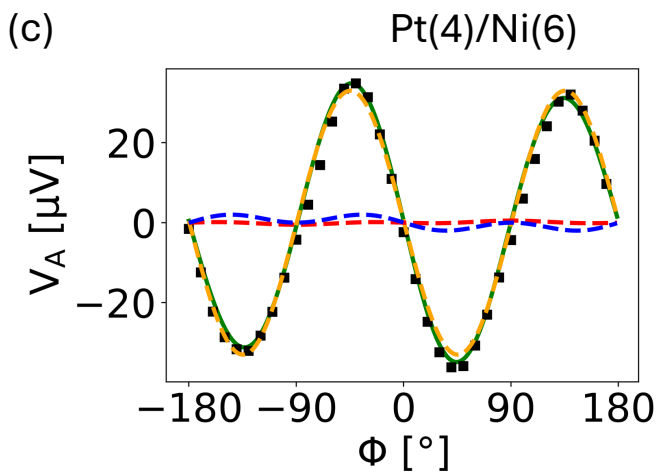

Supplement: Supplementary file 2 [file nl5c02641_si_002.zip › Asym_changedlabels.pdf]

Nb(4)/Ni(6)

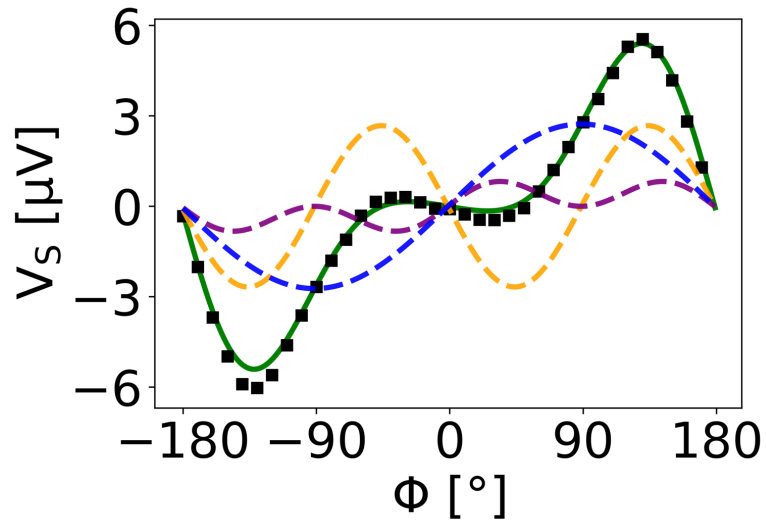

Nb(4)/FeCoB(8)

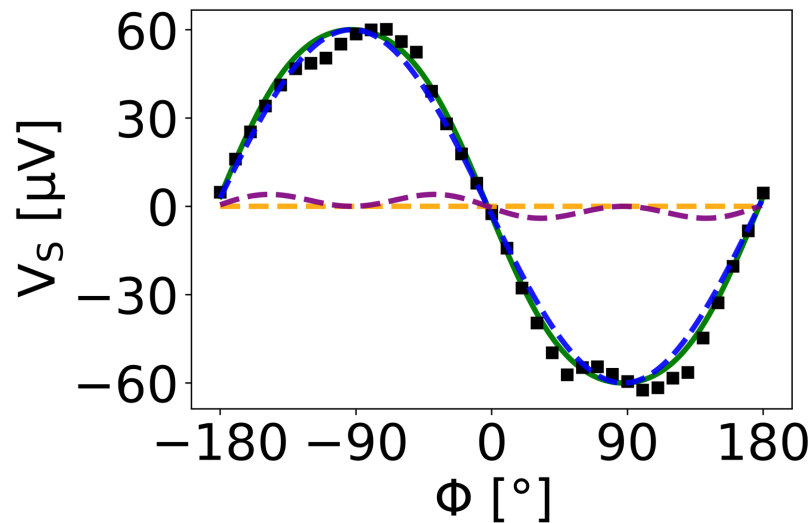

Pt(4)/Ni(6)

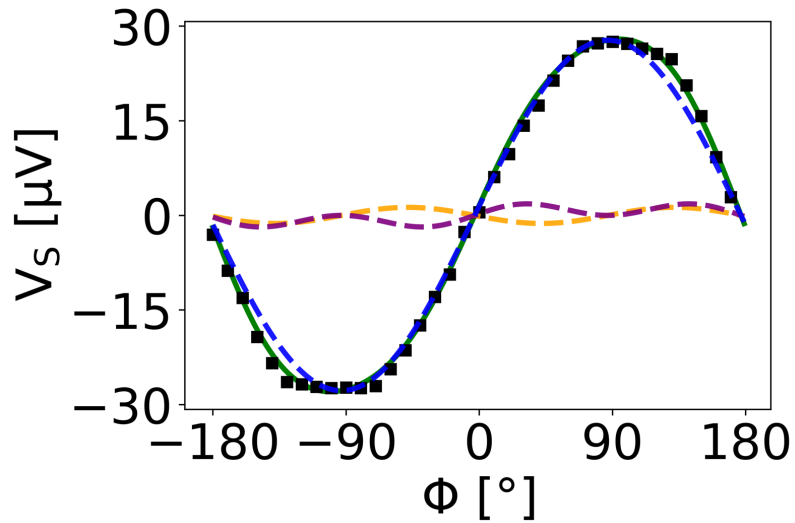

Pt(4)/FeCoB(8)

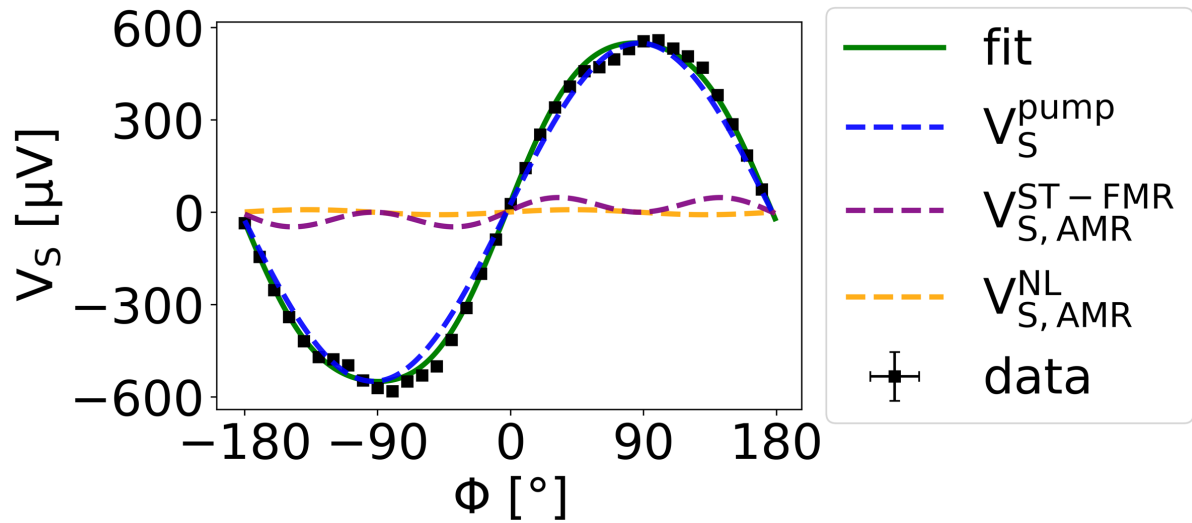

Supplement: Supplementary file 2 [file nl5c02641_si_002.zip › 3components_noitalic.pdf]
